# MetaQTL: a package of new computational methods for the meta-analysis of QTL mapping experiments

**DOI:** 10.1186/1471-2105-8-49

**Published:** 2007-02-08

**Authors:** Jean-Baptiste Veyrieras, Bruno Goffinet, Alain Charcosset

**Affiliations:** 1 UMR, INRA UPS-XI INAPG CNRS Génétique Végétale, Ferme du Moulon, 91190 Gif-sur-Yvette, France; 2 BIA, Chemin de Borde Rouge BP27 31326, Castanet Tolosan Cedex, France

## Abstract

**Background:**

Integration of multiple results from Quantitative Trait Loci (QTL) studies is a key point to understand the genetic determinism of complex traits. Up to now many efforts have been made by public database developers to facilitate the storage, compilation and visualization of multiple QTL mapping experiment results. However, studying the congruency between these results still remains a complex task. Presently, the few computational and statistical frameworks to do so are mainly based on empirical methods (e.g. consensus genetic maps are generally built by iterative projection).

**Results:**

In this article, we present a new computational and statistical package, called MetaQTL, for carrying out whole-genome meta-analysis of QTL mapping experiments. Contrary to existing methods, MetaQTL offers a complete statistical process to establish a consensus model for both the marker and the QTL positions on the whole genome. First, MetaQTL implements a new statistical approach to merge multiple distinct genetic maps into a single consensus map which is optimal in terms of weighted least squares and can be used to investigate recombination rate heterogeneity between studies. Secondly, assuming that QTL can be projected on the consensus map, MetaQTL offers a new clustering approach based on a Gaussian mixture model to decide how many QTL underly the distribution of the observed QTL.

**Conclusion:**

We demonstrate using simulations that the usual model choice criteria from mixture model literature perform relatively well in this context. As expected, simulations also show that this new clustering algorithm leads to a reduction in the length of the confidence interval of QTL location provided that across studies there are enough observed QTL for each underlying true QTL location. The usefulness of our approach is illustrated on published QTL detection results of flowering time in maize. Finally, MetaQTL is freely available at .

## Background

In the last two decades, the advent of molecular markers and their use in linkage mapping experiments has tremendously increased the potential of quantitative genetics. Linkage mapping experiments now provide an efficient tool to identify regions of the genome where polymorphism affects the variation of quantitative traits, called Quantitative Trait Loci (QTL). Although a large number of advanced statistical methods have been developed to improve the localization of QTL, the limited number of recombination events available in routinely used pedigree designs for QTL mapping lead essentially to an approximate mapping of the QTL (see for instance [1]). This is mainly due to both a few mating generations and a restricted number of sampled individuals (generally a few hundreds). From results of QTL experiments gathered over a wide range of plant species, [2] have shown that confidence intervals around most likely QTL positions are, on average, approximately 10 cM, which usually includes several hundreds of genes. More recent advents in the area of molecular biology have allowed researchers to carry out positional cloning of QTL (see for instance the review of [3]) but this approach still remains extremely expensive both in terms of time and resources. Also several authors [2,4] have pointed out that QTL detection is statistically biased both in the true number of QTL, which is underestimated since only QTL with large effects are detected, and in the QTL effects which are over estimated as only significant effects are reported (a phenomenon has commonly referred to as the Beavis effect [5]). Even though QTL mapping experiments must be considered with an awareness of these limitations, they have become commonplace and have greatly improved our knowledge about the genetic component of complex traits.

Since the first publication of a QTL localization using molecular data [6], more and more species and traits have been studied and many of these results has been made available via public databases. One of the main purposes of these databases was to help researchers to compare results from different QTL studies, to study the congruency of QTL locations in order to address the following question: "do QTL identified for a given trait in a population correspond to those detected in other populations ?". In theory one would expect that the variation of a quantitative trait within a species is explained by a finite number of genes. Thus QTL congruency investigation will be a relevant approach to improve knowledge on trait genetics and several publications have pointed out its usefulness [7-12]. Nevertheless, the combination of results from linkage studies can be tedious since, even if several studies focus on the same trait within the same species, family structures, sample sizes, marker maps, or QTL detection methods may differ between studies. Some methods have been recently developed to tackle the issues raised by between QTL studies heterogeneity. Integration of genetic maps and QTL locations by iterative projections on a reference map is now widely used to position both markers and QTL on a single and homogeneous consensus map (see for instance [13]). However this process yields a consensus marker map for which both the statistical properties and biological "reality" can't be clearly assessed, even if a robust ordered marker map was used as reference. [14] proposed an original approach using graph theory to integrate various types of maps (genetic, physical or sequence-based) but it mainly dealt with dissection of marker order inconsistencies between maps. From up to now it seems that there is no efficient methodological framework to build reliable consensus marker maps on which markers and candidate genes from different mapping experiments can be both ordered and positioned (except by merging raw mapping data from multiple populations as proposed by [15] and [16]).

In order to study QTL congruency, [17] proposed an original approach based on a meta-analysis strategy. Meta-analysis, which is mainly used in medical, social, and behavioral sciences, aims to pool results across independent studies in order to combine them in a single result or estimate. The relevance of meta-analysis investigations in genetics and evolution has been discussed and pointed out by several authors in the last decade (see for instance [18-21]). More recently [22] developed another meta-analysis based approach to overcome the between-study heterogeneity and to refine both QTL location and the magnitude of the genetic effects. Yet both the method of [17] and [22] are limited to a small number of underlying QTL positions (from one to four for the former and only one for the later) which is a serious limitation for a whole genome study of QTL congruency. Even if the average number of QTL per experiment is around four in plants [2,12], one would expect that more than four genes can be involved in the trait variation on a single chromosome.

To remove these impediments we have developed a new 2-stage meta-analysis procedure in order to integrate multiple independent QTL mapping experiments. Our aim was to create a global framework to evaluate the homogeneity of both genetic marker and QTL mapping results from literature and public databases. The first part of our meta-analysis procedure consists in building a consensus genetic marker map that takes into account the statistical properties of genetic distance estimates using a Weighted Least Squares (WLS) strategy. Secondly, once the consensus marker map has been built, the QTL locations can be projected on to the map. We also propose a new clustering algorithm based on a Gaussian mixture model in order to identify the number of underlying QTL which best explain the observed distribution of QTL positions in the mapping experiments. As it has been emphasized by [17], the crucial point at this step is to find an unbiased criterion to select the correct number of QTL. In the context of Gaussian mixture, a large variety of model choice criteria have been reported in the literature. We explore, by means of simulations, the properties of some of these criteria for our particular mixture model. These new methods have been implemented into a Java package called **MetaQTL**. Finally, as an example, we applied our new approach to QTL detection results gathered for flowering time in maize.

## Results

### Meta-analysis of genetic maps

#### Input genetic map information

Consider a set of *n *genetic mapping experiments concerning the same linkage group. These different experiments may involve different kinds of population pedigree. We consider that for each experiment *i *= 1, ..., *n *only the estimated distances between ordered markers along the linkage group are available. We use *c*_*i*_, *N*_*i*_, *M*_*i *_to denote the population cross design, the population size and the number or markers on the *i*^th ^genetic map, respectively. Let's suppose that two markers *m*_*j *_and *m*_*k *_have been positioned on the *i*^th ^map, r^
 MathType@MTEF@5@5@+=feaafiart1ev1aaatCvAUfKttLearuWrP9MDH5MBPbIqV92AaeXatLxBI9gBaebbnrfifHhDYfgasaacH8akY=wiFfYdH8Gipec8Eeeu0xXdbba9frFj0=OqFfea0dXdd9vqai=hGuQ8kuc9pgc9s8qqaq=dirpe0xb9q8qiLsFr0=vr0=vr0dc8meaabaqaciaacaGaaeqabaqabeGadaaakeaacuWGYbGCgaqcaaaa@2E29@_*i*,*jk *_stands for the estimated recombination rate between markers *m*_*j *_and *m*_*k *_and d^
 MathType@MTEF@5@5@+=feaafiart1ev1aaatCvAUfKttLearuWrP9MDH5MBPbIqV92AaeXatLxBI9gBaebbnrfifHhDYfgasaacH8akY=wiFfYdH8Gipec8Eeeu0xXdbba9frFj0=OqFfea0dXdd9vqai=hGuQ8kuc9pgc9s8qqaq=dirpe0xb9q8qiLsFr0=vr0=vr0dc8meaabaqaciaacaGaaeqabaqabeGadaaakeaacuWGKbazgaqcaaaa@2E0D@_*i*,*jk *_= *f*[r^
 MathType@MTEF@5@5@+=feaafiart1ev1aaatCvAUfKttLearuWrP9MDH5MBPbIqV92AaeXatLxBI9gBaebbnrfifHhDYfgasaacH8akY=wiFfYdH8Gipec8Eeeu0xXdbba9frFj0=OqFfea0dXdd9vqai=hGuQ8kuc9pgc9s8qqaq=dirpe0xb9q8qiLsFr0=vr0=vr0dc8meaabaqaciaacaGaaeqabaqabeGadaaakeaacuWGYbGCgaqcaaaa@2E29@_*i*,*jk*_] the corresponding estimated distance, where *f *is the mapping function which is assumed to be the same in the *n *mapping experiments (without loss of generality). Applying the classical asymptotic Gaussian distribution of the maximum-likelihood estimation of the parameter, we assume that the r^
 MathType@MTEF@5@5@+=feaafiart1ev1aaatCvAUfKttLearuWrP9MDH5MBPbIqV92AaeXatLxBI9gBaebbnrfifHhDYfgasaacH8akY=wiFfYdH8Gipec8Eeeu0xXdbba9frFj0=OqFfea0dXdd9vqai=hGuQ8kuc9pgc9s8qqaq=dirpe0xb9q8qiLsFr0=vr0=vr0dc8meaabaqaciaacaGaaeqabaqabeGadaaakeaacuWGYbGCgaqcaaaa@2E29@_*i*,*jk *_are normally distributed around the true recombination rate *r*_*i*,*jk *_between markers *m*_*j *_and *m*_*k *_with a variance var(r^
 MathType@MTEF@5@5@+=feaafiart1ev1aaatCvAUfKttLearuWrP9MDH5MBPbIqV92AaeXatLxBI9gBaebbnrfifHhDYfgasaacH8akY=wiFfYdH8Gipec8Eeeu0xXdbba9frFj0=OqFfea0dXdd9vqai=hGuQ8kuc9pgc9s8qqaq=dirpe0xb9q8qiLsFr0=vr0=vr0dc8meaabaqaciaacaGaaeqabaqabeGadaaakeaacuWGYbGCgaqcaaaa@2E29@_*i*,*jk*_) = ηi,jk2
 MathType@MTEF@5@5@+=feaafiart1ev1aaatCvAUfKttLearuWrP9MDH5MBPbIqV92AaeXatLxBI9gBaebbnrfifHhDYfgasaacH8akY=wiFfYdH8Gipec8Eeeu0xXdbba9frFj0=OqFfea0dXdd9vqai=hGuQ8kuc9pgc9s8qqaq=dirpe0xb9q8qiLsFr0=vr0=vr0dc8meaabaqaciaacaGaaeqabaqabeGadaaakeaaiiGacqWF3oaAdaqhaaWcbaGaemyAaKMaeiilaWIaemOAaOMaem4AaSgabaGaeGOmaidaaaaa@3475@. This variance ηi,jk2
 MathType@MTEF@5@5@+=feaafiart1ev1aaatCvAUfKttLearuWrP9MDH5MBPbIqV92AaeXatLxBI9gBaebbnrfifHhDYfgasaacH8akY=wiFfYdH8Gipec8Eeeu0xXdbba9frFj0=OqFfea0dXdd9vqai=hGuQ8kuc9pgc9s8qqaq=dirpe0xb9q8qiLsFr0=vr0=vr0dc8meaabaqaciaacaGaaeqabaqabeGadaaakeaaiiGacqWF3oaAdaqhaaWcbaGaemyAaKMaeiilaWIaemOAaOMaem4AaSgabaGaeGOmaidaaaaa@3475@ depends on the cross design *c*_*i*_, the value of *r*_*i*,*jk*_, the sample size *N*_*i *_and the amount of information supplied by the marker pair *m*_*j *_and *m*_*k *_sampled population (see Additional File 1 for expression of *η*).

Since mapping functions are generally one to one functions, the functional invariance property of the maximum-likelihood estimate can be applied. Thus d^
 MathType@MTEF@5@5@+=feaafiart1ev1aaatCvAUfKttLearuWrP9MDH5MBPbIqV92AaeXatLxBI9gBaebbnrfifHhDYfgasaacH8akY=wiFfYdH8Gipec8Eeeu0xXdbba9frFj0=OqFfea0dXdd9vqai=hGuQ8kuc9pgc9s8qqaq=dirpe0xb9q8qiLsFr0=vr0=vr0dc8meaabaqaciaacaGaaeqabaqabeGadaaakeaacuWGKbazgaqcaaaa@2E0D@_*i*,*jk *_is also normally distributed around the true distance denoted *d*_*i*,*jk *_= *f*[*r*_*i*,*jk*_]. To obtain the variance of d^
 MathType@MTEF@5@5@+=feaafiart1ev1aaatCvAUfKttLearuWrP9MDH5MBPbIqV92AaeXatLxBI9gBaebbnrfifHhDYfgasaacH8akY=wiFfYdH8Gipec8Eeeu0xXdbba9frFj0=OqFfea0dXdd9vqai=hGuQ8kuc9pgc9s8qqaq=dirpe0xb9q8qiLsFr0=vr0=vr0dc8meaabaqaciaacaGaaeqabaqabeGadaaakeaacuWGKbazgaqcaaaa@2E0D@_*i*,*jk*_, denoted γi,jk2
 MathType@MTEF@5@5@+=feaafiart1ev1aaatCvAUfKttLearuWrP9MDH5MBPbIqV92AaeXatLxBI9gBaebbnrfifHhDYfgasaacH8akY=wiFfYdH8Gipec8Eeeu0xXdbba9frFj0=OqFfea0dXdd9vqai=hGuQ8kuc9pgc9s8qqaq=dirpe0xb9q8qiLsFr0=vr0=vr0dc8meaabaqaciaacaGaaeqabaqabeGadaaakeaaiiGacqWFZoWzdaqhaaWcbaGaemyAaKMaeiilaWIaemOAaOMaem4AaSgabaGaeGOmaidaaaaa@3470@, we use the first term of the Taylor expansion of the inverse of the mapping function leading to the approximation:

γi,jk2=var⁡(d^i,jk)≈ηi,jk2×(∂f[r^i,jk]∂r)2
 MathType@MTEF@5@5@+=feaafiart1ev1aaatCvAUfKttLearuWrP9MDH5MBPbIqV92AaeXatLxBI9gBaebbnrfifHhDYfgasaacH8akY=wiFfYdH8Gipec8Eeeu0xXdbba9frFj0=OqFfea0dXdd9vqai=hGuQ8kuc9pgc9s8qqaq=dirpe0xb9q8qiLsFr0=vr0=vr0dc8meaabaqaciaacaGaaeqabaqabeGadaaakeaaiiGacqWFZoWzdaqhaaWcbaGaemyAaKMaeiilaWIaemOAaOMaem4AaSgabaGaeGOmaidaaOGaeyypa0JagiODayNaeiyyaeMaeiOCaiNaeiikaGIafmizaqMbaKaadaWgaaWcbaGaemyAaKMaeiilaWIaemOAaOMaem4AaSgabeaakiabcMcaPiabgIKi7kab=D7aOnaaDaaaleaacqWGPbqAcqGGSaalcqWGQbGAcqWGRbWAaeaacqaIYaGmaaGccqGHxdaTdaqadaqaamaalaaabaGae8NaIyRaemOzayMaei4waSLafmOCaiNbaKaadaWgaaWcbaGaemyAaKMaeiilaWIaemOAaOMaem4AaSgabeaakiabc2faDbqaaiab=jGi2kabdkhaYbaaaiaawIcacaGLPaaadaahaaWcbeqaaiabikdaYaaaaaa@5EDD@

Now suppose the *n *experiments are consistent with the following assumptions:

• Assumption 1 : they come from independent population samples. This implies that cov(r^
 MathType@MTEF@5@5@+=feaafiart1ev1aaatCvAUfKttLearuWrP9MDH5MBPbIqV92AaeXatLxBI9gBaebbnrfifHhDYfgasaacH8akY=wiFfYdH8Gipec8Eeeu0xXdbba9frFj0=OqFfea0dXdd9vqai=hGuQ8kuc9pgc9s8qqaq=dirpe0xb9q8qiLsFr0=vr0=vr0dc8meaabaqaciaacaGaaeqabaqabeGadaaakeaacuWGYbGCgaqcaaaa@2E29@_*i*,*jk*_, r^
 MathType@MTEF@5@5@+=feaafiart1ev1aaatCvAUfKttLearuWrP9MDH5MBPbIqV92AaeXatLxBI9gBaebbnrfifHhDYfgasaacH8akY=wiFfYdH8Gipec8Eeeu0xXdbba9frFj0=OqFfea0dXdd9vqai=hGuQ8kuc9pgc9s8qqaq=dirpe0xb9q8qiLsFr0=vr0=vr0dc8meaabaqaciaacaGaaeqabaqabeGadaaakeaacuWGYbGCgaqcaaaa@2E29@_*i'*,*jk*_) = 0 and cov(d^
 MathType@MTEF@5@5@+=feaafiart1ev1aaatCvAUfKttLearuWrP9MDH5MBPbIqV92AaeXatLxBI9gBaebbnrfifHhDYfgasaacH8akY=wiFfYdH8Gipec8Eeeu0xXdbba9frFj0=OqFfea0dXdd9vqai=hGuQ8kuc9pgc9s8qqaq=dirpe0xb9q8qiLsFr0=vr0=vr0dc8meaabaqaciaacaGaaeqabaqabeGadaaakeaacuWGKbazgaqcaaaa@2E0D@_*i*,*jk*_, d^
 MathType@MTEF@5@5@+=feaafiart1ev1aaatCvAUfKttLearuWrP9MDH5MBPbIqV92AaeXatLxBI9gBaebbnrfifHhDYfgasaacH8akY=wiFfYdH8Gipec8Eeeu0xXdbba9frFj0=OqFfea0dXdd9vqai=hGuQ8kuc9pgc9s8qqaq=dirpe0xb9q8qiLsFr0=vr0=vr0dc8meaabaqaciaacaGaaeqabaqabeGadaaakeaacuWGKbazgaqcaaaa@2E0D@_*i'*,*jk*_) = 0 for any pair of markers *m*_*j *_and *m*_*k *_which have been mapped in population *i *and *i'*, *i *≠ *i' *and (*i*, *i'*) ∈ [1..*n*]^2 ^.

• Assumption 2 : there is no interference, i.e in each mapping experiment the recombination events occur independently in each marker interval. This is surely an idealization, but presently, most of the statistical models used to build genetic marker maps are based on this assumption. Thus for a given mapping experiment *i*, both the ordered marker interval recombination rate and distance estimates are independent, i.e cov(r^
 MathType@MTEF@5@5@+=feaafiart1ev1aaatCvAUfKttLearuWrP9MDH5MBPbIqV92AaeXatLxBI9gBaebbnrfifHhDYfgasaacH8akY=wiFfYdH8Gipec8Eeeu0xXdbba9frFj0=OqFfea0dXdd9vqai=hGuQ8kuc9pgc9s8qqaq=dirpe0xb9q8qiLsFr0=vr0=vr0dc8meaabaqaciaacaGaaeqabaqabeGadaaakeaacuWGYbGCgaqcaaaa@2E29@_*i*,*j*(*j*+1)_, r^
 MathType@MTEF@5@5@+=feaafiart1ev1aaatCvAUfKttLearuWrP9MDH5MBPbIqV92AaeXatLxBI9gBaebbnrfifHhDYfgasaacH8akY=wiFfYdH8Gipec8Eeeu0xXdbba9frFj0=OqFfea0dXdd9vqai=hGuQ8kuc9pgc9s8qqaq=dirpe0xb9q8qiLsFr0=vr0=vr0dc8meaabaqaciaacaGaaeqabaqabeGadaaakeaacuWGYbGCgaqcaaaa@2E29@_*i*,(*j*+1)(*j*+2)_) = 0 and cov(d^
 MathType@MTEF@5@5@+=feaafiart1ev1aaatCvAUfKttLearuWrP9MDH5MBPbIqV92AaeXatLxBI9gBaebbnrfifHhDYfgasaacH8akY=wiFfYdH8Gipec8Eeeu0xXdbba9frFj0=OqFfea0dXdd9vqai=hGuQ8kuc9pgc9s8qqaq=dirpe0xb9q8qiLsFr0=vr0=vr0dc8meaabaqaciaacaGaaeqabaqabeGadaaakeaacuWGKbazgaqcaaaa@2E0D@_*i*,*j*(*j*+1)_, d^
 MathType@MTEF@5@5@+=feaafiart1ev1aaatCvAUfKttLearuWrP9MDH5MBPbIqV92AaeXatLxBI9gBaebbnrfifHhDYfgasaacH8akY=wiFfYdH8Gipec8Eeeu0xXdbba9frFj0=OqFfea0dXdd9vqai=hGuQ8kuc9pgc9s8qqaq=dirpe0xb9q8qiLsFr0=vr0=vr0dc8meaabaqaciaacaGaaeqabaqabeGadaaakeaacuWGKbazgaqcaaaa@2E0D@_*i*(*j*+1)(*j*+2)_) = 0 for *i *∈ [1,..., *n*] and *j *∈ [1,..., *M*_*i *_- 2].

• Assumption 3 : the "true" marker order and recombination rate are supposed to be the same in the different populations, i.e *r*_*i*,*jk *_= *r*_*i'*,*jk *_if markers *m*_*j *_and *m*_*k *_have been mapped in population *i *and *i'*, *i *≠ *i' *and (*i*, *i'*) ∈ [1..*n*]^2^.

• Assumption 4 : all the genetic maps are connected. Mathematically, this means that if we consider maps as vertices and common markers as edges, then the corresponding graph is supposed to be connected.

#### Meta-analysis model

We define D^
 MathType@MTEF@5@5@+=feaafiart1ev1aaatCvAUfKttLearuWrP9MDH5MBPbIqV92AaeXatLxBI9gBaebbnrfifHhDYfgasaacH8akY=wiFfYdH8Gipec8Eeeu0xXdbba9frFj0=OqFfea0dXdd9vqai=hGuQ8kuc9pgc9s8qqaq=dirpe0xb9q8qiLsFr0=vr0=vr0dc8meaabaqaciaacaGaaeqabaqabeGadaaakeaacuWGebargaqcaaaa@2DCD@ = (d^
 MathType@MTEF@5@5@+=feaafiart1ev1aaatCvAUfKttLearuWrP9MDH5MBPbIqV92AaeXatLxBI9gBaebbnrfifHhDYfgasaacH8akY=wiFfYdH8Gipec8Eeeu0xXdbba9frFj0=OqFfea0dXdd9vqai=hGuQ8kuc9pgc9s8qqaq=dirpe0xb9q8qiLsFr0=vr0=vr0dc8meaabaqaciaacaGaaeqabaqabeGadaaakeaacuWGKbazgaqcaaaa@2E0D@_*i*,*jk*_) and Γ = diag(γi,jk2
 MathType@MTEF@5@5@+=feaafiart1ev1aaatCvAUfKttLearuWrP9MDH5MBPbIqV92AaeXatLxBI9gBaebbnrfifHhDYfgasaacH8akY=wiFfYdH8Gipec8Eeeu0xXdbba9frFj0=OqFfea0dXdd9vqai=hGuQ8kuc9pgc9s8qqaq=dirpe0xb9q8qiLsFr0=vr0=vr0dc8meaabaqaciaacaGaaeqabaqabeGadaaakeaaiiGacqWFZoWzdaqhaaWcbaGaemyAaKMaeiilaWIaemOAaOMaem4AaSgabaGaeGOmaidaaaaa@3470@) the vector of ordered marker interval distance estimates and the diagonal terms of the variance covariance matrix of D^
 MathType@MTEF@5@5@+=feaafiart1ev1aaatCvAUfKttLearuWrP9MDH5MBPbIqV92AaeXatLxBI9gBaebbnrfifHhDYfgasaacH8akY=wiFfYdH8Gipec8Eeeu0xXdbba9frFj0=OqFfea0dXdd9vqai=hGuQ8kuc9pgc9s8qqaq=dirpe0xb9q8qiLsFr0=vr0=vr0dc8meaabaqaciaacaGaaeqabaqabeGadaaakeaacuWGebargaqcaaaa@2DCD@. We assume that a total of *M *distinct markers have been mapped in the *n *populations. The aim of the meta-analysis is to combine all the available information on marker order and positions in order to build a consensus linkage group on which the *M *markers are positioned. To do so we introduce *Y *= (*y*_1_,..., *y*_*M*_) the vector of the "true" positions of these *M *markers on the consensus linkage group, where the *y*_*i*_'s can be either positive or negative depending on an arbitrary zero-reference on the chromosome (hereafter we suppose *y*_1 _= 0). If the *n *mapping experiments are consistent with the previous assumptions and assuming that the distances on the linkage group are additive we propose to estimate *Y *by solving the following linear system:

d^
 MathType@MTEF@5@5@+=feaafiart1ev1aaatCvAUfKttLearuWrP9MDH5MBPbIqV92AaeXatLxBI9gBaebbnrfifHhDYfgasaacH8akY=wiFfYdH8Gipec8Eeeu0xXdbba9frFj0=OqFfea0dXdd9vqai=hGuQ8kuc9pgc9s8qqaq=dirpe0xb9q8qiLsFr0=vr0=vr0dc8meaabaqaciaacaGaaeqabaqabeGadaaakeaacuWGKbazgaqcaaaa@2E0D@_*i*,*jk *_= *y*_*k *_- *y*_*j *_+ *ε*_*i*,*jk*_

where (*j*, *k*) ∈ [1,...,*M*]^2^, *i *∈ [1,..., *n*], d^
 MathType@MTEF@5@5@+=feaafiart1ev1aaatCvAUfKttLearuWrP9MDH5MBPbIqV92AaeXatLxBI9gBaebbnrfifHhDYfgasaacH8akY=wiFfYdH8Gipec8Eeeu0xXdbba9frFj0=OqFfea0dXdd9vqai=hGuQ8kuc9pgc9s8qqaq=dirpe0xb9q8qiLsFr0=vr0=vr0dc8meaabaqaciaacaGaaeqabaqabeGadaaakeaacuWGKbazgaqcaaaa@2E0D@_*i*,*jk *_is the distance estimate of the interval between marker *m*_*j *_and *m*_*k *_consecutive on the *i*^th ^experiment, *y*_*k *_- *y*_*j *_is the true distance between these markers, *ε*_*i*,*jk *_~ N
 MathType@MTEF@5@5@+=feaafiart1ev1aaatCvAUfKttLearuWrP9MDH5MBPbIqV92AaeXatLxBI9gBamrtHrhAL1wy0L2yHvtyaeHbnfgDOvwBHrxAJfwnaebbnrfifHhDYfgasaacH8akY=wiFfYdH8Gipec8Eeeu0xXdbba9frFj0=OqFfea0dXdd9vqai=hGuQ8kuc9pgc9s8qqaq=dirpe0xb9q8qiLsFr0=vr0=vr0dc8meaabaqaciaacaGaaeqabaWaaeGaeaaakeaaimaacqWFneVtaaa@383B@(0, γi,jk2
 MathType@MTEF@5@5@+=feaafiart1ev1aaatCvAUfKttLearuWrP9MDH5MBPbIqV92AaeXatLxBI9gBaebbnrfifHhDYfgasaacH8akY=wiFfYdH8Gipec8Eeeu0xXdbba9frFj0=OqFfea0dXdd9vqai=hGuQ8kuc9pgc9s8qqaq=dirpe0xb9q8qiLsFr0=vr0=vr0dc8meaabaqaciaacaGaaeqabaqabeGadaaakeaaiiGacqWFZoWzdaqhaaWcbaGaemyAaKMaeiilaWIaemOAaOMaem4AaSgabaGaeGOmaidaaaaa@3470@) is the error term. If assumption 4 holds we are ensured that this system has at least one solution. Applying a classical weighted least squares (WLS) strategy, the optimal solution is the one which minimizes the target function,

χ=∑i=1n∑jk[d^i,jk−(yk−yj)]2γi,jk2
 MathType@MTEF@5@5@+=feaafiart1ev1aaatCvAUfKttLearuWrP9MDH5MBPbIqV92AaeXatLxBI9gBaebbnrfifHhDYfgasaacH8akY=wiFfYdH8Gipec8Eeeu0xXdbba9frFj0=OqFfea0dXdd9vqai=hGuQ8kuc9pgc9s8qqaq=dirpe0xb9q8qiLsFr0=vr0=vr0dc8meaabaqaciaacaGaaeqabaqabeGadaaakeaaiiGacqWFhpWycqGH9aqpdaaeWbqaamaaqafabaWaaSaaaeaacqGGBbWwcuWGKbazgaqcamaaBaaaleaacqWGPbqAcqGGSaalcqWGQbGAcqWGRbWAaeqaaOGaeyOeI0IaeiikaGIaemyEaK3aaSbaaSqaaiabdUgaRbqabaGccqGHsislcqWG5bqEdaWgaaWcbaGaemOAaOgabeaakiabcMcaPiabc2faDnaaCaaaleqabaGaeGOmaidaaaGcbaGae83SdC2aa0baaSqaaiabdMgaPjabcYcaSiabdQgaQjabdUgaRbqaaiabikdaYaaaaaaabaGaemOAaOMaem4AaSgabeqdcqGHris5aaWcbaGaemyAaKMaeyypa0JaeGymaedabaGaemOBa4ganiabggHiLdaaaa@56E5@

Let's introduce the design matrix *A *such that *χ *= ^T^(D^
 MathType@MTEF@5@5@+=feaafiart1ev1aaatCvAUfKttLearuWrP9MDH5MBPbIqV92AaeXatLxBI9gBaebbnrfifHhDYfgasaacH8akY=wiFfYdH8Gipec8Eeeu0xXdbba9frFj0=OqFfea0dXdd9vqai=hGuQ8kuc9pgc9s8qqaq=dirpe0xb9q8qiLsFr0=vr0=vr0dc8meaabaqaciaacaGaaeqabaqabeGadaaakeaacuWGebargaqcaaaa@2DCD@ - *AY*)Γ^-1^(D^
 MathType@MTEF@5@5@+=feaafiart1ev1aaatCvAUfKttLearuWrP9MDH5MBPbIqV92AaeXatLxBI9gBaebbnrfifHhDYfgasaacH8akY=wiFfYdH8Gipec8Eeeu0xXdbba9frFj0=OqFfea0dXdd9vqai=hGuQ8kuc9pgc9s8qqaq=dirpe0xb9q8qiLsFr0=vr0=vr0dc8meaabaqaciaacaGaaeqabaqabeGadaaakeaacuWGebargaqcaaaa@2DCD@ - *AY*). Then the value of *Y *which minimizes *χ *is given by:

Y^
 MathType@MTEF@5@5@+=feaafiart1ev1aaatCvAUfKttLearuWrP9MDH5MBPbIqV92AaeXatLxBI9gBaebbnrfifHhDYfgasaacH8akY=wiFfYdH8Gipec8Eeeu0xXdbba9frFj0=OqFfea0dXdd9vqai=hGuQ8kuc9pgc9s8qqaq=dirpe0xb9q8qiLsFr0=vr0=vr0dc8meaabaqaciaacaGaaeqabaqabeGadaaakeaacuWGzbqwgaqcaaaa@2DF7@ = (^T^*A*Γ^-1 ^*A*)^-1^(^T^*A*Γ^-1 ^D^
 MathType@MTEF@5@5@+=feaafiart1ev1aaatCvAUfKttLearuWrP9MDH5MBPbIqV92AaeXatLxBI9gBaebbnrfifHhDYfgasaacH8akY=wiFfYdH8Gipec8Eeeu0xXdbba9frFj0=OqFfea0dXdd9vqai=hGuQ8kuc9pgc9s8qqaq=dirpe0xb9q8qiLsFr0=vr0=vr0dc8meaabaqaciaacaGaaeqabaqabeGadaaakeaacuWGebargaqcaaaa@2DCD@)

which is also a maximum-likelihood estimation of *Y *with variance-covariance matrix given by (^T^*A*Γ^-1 ^*A*)^-1^. Thus Y^
 MathType@MTEF@5@5@+=feaafiart1ev1aaatCvAUfKttLearuWrP9MDH5MBPbIqV92AaeXatLxBI9gBaebbnrfifHhDYfgasaacH8akY=wiFfYdH8Gipec8Eeeu0xXdbba9frFj0=OqFfea0dXdd9vqai=hGuQ8kuc9pgc9s8qqaq=dirpe0xb9q8qiLsFr0=vr0=vr0dc8meaabaqaciaacaGaaeqabaqabeGadaaakeaacuWGzbqwgaqcaaaa@2DF7@ gives both the marker positions and the marker order along the consensus linkage group. The goodness-of-fit of the model can be evaluated by the means of a chi-square test as *χ *~ χq−M+12
 MathType@MTEF@5@5@+=feaafiart1ev1aaatCvAUfKttLearuWrP9MDH5MBPbIqV92AaeXatLxBI9gBaebbnrfifHhDYfgasaacH8akY=wiFfYdH8Gipec8Eeeu0xXdbba9frFj0=OqFfea0dXdd9vqai=hGuQ8kuc9pgc9s8qqaq=dirpe0xb9q8qiLsFr0=vr0=vr0dc8meaabaqaciaacaGaaeqabaqabeGadaaakeaaiiGacqWFhpWydaqhaaWcbaGaemyCaeNaeyOeI0Iaemyta0Kaey4kaSIaeGymaedabaGaeGOmaidaaaaa@34D6@ where *q *is the length of the vector D^
 MathType@MTEF@5@5@+=feaafiart1ev1aaatCvAUfKttLearuWrP9MDH5MBPbIqV92AaeXatLxBI9gBaebbnrfifHhDYfgasaacH8akY=wiFfYdH8Gipec8Eeeu0xXdbba9frFj0=OqFfea0dXdd9vqai=hGuQ8kuc9pgc9s8qqaq=dirpe0xb9q8qiLsFr0=vr0=vr0dc8meaabaqaciaacaGaaeqabaqabeGadaaakeaacuWGebargaqcaaaa@2DCD@, i.e the number of marker intervals over the *n *experiments. As an illustration, let's consider the following idealized scenario : suppose that the *n *gathered genetic maps share the same markers, i.e *M*_*i *_= *M *for *i *= 1,..., *n*. In this simple case the computation of Y^
 MathType@MTEF@5@5@+=feaafiart1ev1aaatCvAUfKttLearuWrP9MDH5MBPbIqV92AaeXatLxBI9gBaebbnrfifHhDYfgasaacH8akY=wiFfYdH8Gipec8Eeeu0xXdbba9frFj0=OqFfea0dXdd9vqai=hGuQ8kuc9pgc9s8qqaq=dirpe0xb9q8qiLsFr0=vr0=vr0dc8meaabaqaciaacaGaaeqabaqabeGadaaakeaacuWGzbqwgaqcaaaa@2DF7@ is straightforward:

{y^1=0y^j+1−y^j=∑i=1nγi,j(j+1)−2d^i,j(j+1)∑i=1nγi,j(j+1)−2 j∈[1,...,M−1]
 MathType@MTEF@5@5@+=feaafiart1ev1aaatCvAUfKttLearuWrP9MDH5MBPbIqV92AaeXatLxBI9gBaebbnrfifHhDYfgasaacH8akY=wiFfYdH8Gipec8Eeeu0xXdbba9frFj0=OqFfea0dXdd9vqai=hGuQ8kuc9pgc9s8qqaq=dirpe0xb9q8qiLsFr0=vr0=vr0dc8meaabaqaciaacaGaaeqabaqabeGadaaakeaadaGabeqaauaabaqacmaaaeaacuWG5bqEgaqcamaaBaaaleaacqaIXaqmaeqaaaGcbaGaeyypa0dabaGaeGimaadabaGafmyEaKNbaKaadaWgaaWcbaGaemOAaOMaey4kaSIaeGymaedabeaakiabgkHiTiqbdMha5zaajaWaaSbaaSqaaiabdQgaQbqabaaakeaacqGH9aqpaeaadaWcaaqaamaaqahabaacciGae83SdC2aa0baaSqaaiabdMgaPjabcYcaSiabdQgaQjabcIcaOiabdQgaQjabgUcaRiabigdaXiabcMcaPaqaaiabgkHiTiabikdaYaaaaeaacqWGPbqAcqGH9aqpcqaIXaqmaeaacqWGUbGBa0GaeyyeIuoakiqbdsgaKzaajaWaaSbaaSqaaiabdMgaPjabcYcaSiabdQgaQjabcIcaOiabdQgaQjabgUcaRiabigdaXiabcMcaPaqabaaakeaadaaeWbqaaiab=n7aNnaaDaaaleaacqWGPbqAcqGGSaalcqWGQbGAcqGGOaakcqWGQbGAcqGHRaWkcqaIXaqmcqGGPaqkaeaacqGHsislcqaIYaGmaaaabaGaemyAaKMaeyypa0JaeGymaedabaGaemOBa4ganiabggHiLdaaaOGaeeiiaaIaemOAaOMaeyicI4Saei4waSLaeGymaeJaeiilaWIaeiOla4IaeiOla4IaeiOla4IaeiilaWIaemyta0KaeyOeI0IaeGymaeJaeiyxa0faaaGaay5Eaaaaaa@7B6B@

and *χ *is the sum of *M *- 1 terms each distributed as a chi-square with *n *- 1 degree of freedoms. This is equivalent to for each marker intervals testing if the distances are homogeneous between populations using a classical test of equal means. This trivial example illustrates how our WLS approach can be used to test for the homogeneity of the recombination rate between several mapping experiments. This can be viewed as an alternative to the M-test devised by [23] when raw data are not available.

### Meta-analysis of QTL

#### Input QTL map information

Suppose that for a given trait a QTL detection has been carried out in the *n *mapping experiments. The minimal information supplied by the *i*^th ^QTL experiment consists of a set of estimated positions of the QTL, denoted x^
 MathType@MTEF@5@5@+=feaafiart1ev1aaatCvAUfKttLearuWrP9MDH5MBPbIqV92AaeXatLxBI9gBaebbnrfifHhDYfgasaacH8akY=wiFfYdH8Gipec8Eeeu0xXdbba9frFj0=OqFfea0dXdd9vqai=hGuQ8kuc9pgc9s8qqaq=dirpe0xb9q8qiLsFr0=vr0=vr0dc8meaabaqaciaacaGaaeqabaqabeGadaaakeaacuWG4baEgaqcaaaa@2E35@_*ij*_, and the corresponding proportion of variance explained by each QTL, the r-squares values *λ*_*ij*_. Here *j *∈ [1,..., *q*_*i*_] and *q*_*i *_is the number of QTL detected in the *i*^th ^mapping experiment on the current linkage group (generally *q*_*i *_= 1 or possibly 2). The confidence intervals (CI) of the x^
 MathType@MTEF@5@5@+=feaafiart1ev1aaatCvAUfKttLearuWrP9MDH5MBPbIqV92AaeXatLxBI9gBaebbnrfifHhDYfgasaacH8akY=wiFfYdH8Gipec8Eeeu0xXdbba9frFj0=OqFfea0dXdd9vqai=hGuQ8kuc9pgc9s8qqaq=dirpe0xb9q8qiLsFr0=vr0=vr0dc8meaabaqaciaacaGaaeqabaqabeGadaaakeaacuWG4baEgaqcaaaa@2E35@_*ij*_'s, denoted *v*_*ij*_, can also be reported. The construction of the CI may have been performed by different approaches:

• support interval [24,25].

• likelihood method [26].

• bootstrapping [27,28].

When the CI is not available it is possible to obtain an approximation of the CI by applying the empirical formula proposed by [29]. By means of intensive simulations they showed that for either a backcross or a F_2 _population the expected CI, at 95% level, can be expressed as CI(95) ≈ 530/(*Nλ*) where *N *is the population size and *λ *the proportion of variance explained by the QTL. More recently [30] have derived simple analytical equations which are in good agreement with the formula of [29].

Whatever the method used to estimate the uncertainty on the QTL locations, we assume that the x^
 MathType@MTEF@5@5@+=feaafiart1ev1aaatCvAUfKttLearuWrP9MDH5MBPbIqV92AaeXatLxBI9gBaebbnrfifHhDYfgasaacH8akY=wiFfYdH8Gipec8Eeeu0xXdbba9frFj0=OqFfea0dXdd9vqai=hGuQ8kuc9pgc9s8qqaq=dirpe0xb9q8qiLsFr0=vr0=vr0dc8meaabaqaciaacaGaaeqabaqabeGadaaakeaacuWG4baEgaqcaaaa@2E35@_*ij*_'s are normally distributed around the true position *x*_*ij *_of the *j*^th ^QTL: x^
 MathType@MTEF@5@5@+=feaafiart1ev1aaatCvAUfKttLearuWrP9MDH5MBPbIqV92AaeXatLxBI9gBaebbnrfifHhDYfgasaacH8akY=wiFfYdH8Gipec8Eeeu0xXdbba9frFj0=OqFfea0dXdd9vqai=hGuQ8kuc9pgc9s8qqaq=dirpe0xb9q8qiLsFr0=vr0=vr0dc8meaabaqaciaacaGaaeqabaqabeGadaaakeaacuWG4baEgaqcaaaa@2E35@_*ij *_~ N
 MathType@MTEF@5@5@+=feaafiart1ev1aaatCvAUfKttLearuWrP9MDH5MBPbIqV92AaeXatLxBI9gBamrtHrhAL1wy0L2yHvtyaeHbnfgDOvwBHrxAJfwnaebbnrfifHhDYfgasaacH8akY=wiFfYdH8Gipec8Eeeu0xXdbba9frFj0=OqFfea0dXdd9vqai=hGuQ8kuc9pgc9s8qqaq=dirpe0xb9q8qiLsFr0=vr0=vr0dc8meaabaqaciaacaGaaeqabaWaaeGaeaaakeaaimaacqWFneVtaaa@383B@(*x*_*ij*_, σij2
 MathType@MTEF@5@5@+=feaafiart1ev1aaatCvAUfKttLearuWrP9MDH5MBPbIqV92AaeXatLxBI9gBaebbnrfifHhDYfgasaacH8akY=wiFfYdH8Gipec8Eeeu0xXdbba9frFj0=OqFfea0dXdd9vqai=hGuQ8kuc9pgc9s8qqaq=dirpe0xb9q8qiLsFr0=vr0=vr0dc8meaabaqaciaacaGaaeqabaqabeGadaaakeaaiiGacqWFdpWCdaqhaaWcbaGaemyAaKMaemOAaOgabaGaeGOmaidaaaaa@324D@) where σij2
 MathType@MTEF@5@5@+=feaafiart1ev1aaatCvAUfKttLearuWrP9MDH5MBPbIqV92AaeXatLxBI9gBaebbnrfifHhDYfgasaacH8akY=wiFfYdH8Gipec8Eeeu0xXdbba9frFj0=OqFfea0dXdd9vqai=hGuQ8kuc9pgc9s8qqaq=dirpe0xb9q8qiLsFr0=vr0=vr0dc8meaabaqaciaacaGaaeqabaqabeGadaaakeaaiiGacqWFdpWCdaqhaaWcbaGaemyAaKMaemOAaOgabaGaeGOmaidaaaaa@324D@ is the variance of the estimated position which can be deduced from the confidence interval *v*_*ij*_. For a CI of *β*% (*β *depends on the method used to compute the CI), the standard deviation *σ*_*ij *_can be estimated as *σ*_*ij *_= *v*_*ij*_/(2*u*_*β*_) where *u*_*β *_is the double-sided *β*-percentile of a centered normalized gaussian. This Gaussian approximation based on the classical asymptotic theory has been suggested by [17], even though this is not perfectly correct for QTL with small effects [31].

Furthermore the *n *QTL mapping experiments are assumed to be consistent with the following assumptions:

• Assumption 1 : they are independent. This can be considered as correct when the individuals measured in the different populations have been generated independently. Independence between experiments *i *and *i' *means independence between x^
 MathType@MTEF@5@5@+=feaafiart1ev1aaatCvAUfKttLearuWrP9MDH5MBPbIqV92AaeXatLxBI9gBaebbnrfifHhDYfgasaacH8akY=wiFfYdH8Gipec8Eeeu0xXdbba9frFj0=OqFfea0dXdd9vqai=hGuQ8kuc9pgc9s8qqaq=dirpe0xb9q8qiLsFr0=vr0=vr0dc8meaabaqaciaacaGaaeqabaqabeGadaaakeaacuWG4baEgaqcaaaa@2E35@_*ij *_and x^
 MathType@MTEF@5@5@+=feaafiart1ev1aaatCvAUfKttLearuWrP9MDH5MBPbIqV92AaeXatLxBI9gBaebbnrfifHhDYfgasaacH8akY=wiFfYdH8Gipec8Eeeu0xXdbba9frFj0=OqFfea0dXdd9vqai=hGuQ8kuc9pgc9s8qqaq=dirpe0xb9q8qiLsFr0=vr0=vr0dc8meaabaqaciaacaGaaeqabaqabeGadaaakeaacuWG4baEgaqcaaaa@2E35@_*i'j*_.

• Assumption 2 : for a given trait there is a finite number of underlying QTL which cosegregate in the mapping experiments: this means that the populations share the same trait determinism with potentially different allelic configurations at the QTL. In other word there is a finite number of true QTL positions on the linkage groups, i.e {*x*_*ij*_|(*i*, *j*) ∈ [1,..., *n*] × [1,..., *q*_*i*_]} can potentially contain redundancy.

In addition to the two previous assumptions we also assume that the detected QTL locations are independent within experiments. This is not really true when the QTL detection does not properly take into account linked QTL. But with the advent of composite interval mapping strategy [32,33] multiple-QTL model can now be fitted by adding properly chosen cofactors which limit the impact of linkage between QTL on the position estimates. Therefore we assume that x^
 MathType@MTEF@5@5@+=feaafiart1ev1aaatCvAUfKttLearuWrP9MDH5MBPbIqV92AaeXatLxBI9gBaebbnrfifHhDYfgasaacH8akY=wiFfYdH8Gipec8Eeeu0xXdbba9frFj0=OqFfea0dXdd9vqai=hGuQ8kuc9pgc9s8qqaq=dirpe0xb9q8qiLsFr0=vr0=vr0dc8meaabaqaciaacaGaaeqabaqabeGadaaakeaacuWG4baEgaqcaaaa@2E35@_*ij *_and x^
 MathType@MTEF@5@5@+=feaafiart1ev1aaatCvAUfKttLearuWrP9MDH5MBPbIqV92AaeXatLxBI9gBaebbnrfifHhDYfgasaacH8akY=wiFfYdH8Gipec8Eeeu0xXdbba9frFj0=OqFfea0dXdd9vqai=hGuQ8kuc9pgc9s8qqaq=dirpe0xb9q8qiLsFr0=vr0=vr0dc8meaabaqaciaacaGaaeqabaqabeGadaaakeaacuWG4baEgaqcaaaa@2E35@_*ij' *_are independent for all *j *≠ *j'*.

#### Pre-processing

The first step is to apply our WLS strategy to the *n *mapping experiments in order to build a consensus linkage group. Then the QTL locations are projected on the consensus linkage group using a simple scaling rule between the original QTL flanking marker interval and the corresponding one on the consensus chromosome. For a given QTL location the new confidence interval (if available) on the consensus linkage group is computed by taking into account the average scaling between the original and the consensus chromosome. This is done by computing the sum over the common marker intervals of the ratio of the interval lengths weighted by the probability that the QTL position lies in this interval. There are two possible strategies to approximate this probability. The first one relies on a rough approximation using a Gaussian distribution around the most likely position x^
 MathType@MTEF@5@5@+=feaafiart1ev1aaatCvAUfKttLearuWrP9MDH5MBPbIqV92AaeXatLxBI9gBaebbnrfifHhDYfgasaacH8akY=wiFfYdH8Gipec8Eeeu0xXdbba9frFj0=OqFfea0dXdd9vqai=hGuQ8kuc9pgc9s8qqaq=dirpe0xb9q8qiLsFr0=vr0=vr0dc8meaabaqaciaacaGaaeqabaqabeGadaaakeaacuWG4baEgaqcaaaa@2E35@_*ij *_of the *j*^th ^QTL, namely Pr(QTL *j *in *m*) = ∫umum+1φ[(u−x^ij)/σij]du∫0Lφ[(u−x^ij)/σij]du
 MathType@MTEF@5@5@+=feaafiart1ev1aaatCvAUfKttLearuWrP9MDH5MBPbIqV92AaeXatLxBI9gBaebbnrfifHhDYfgasaacH8akY=wiFfYdH8Gipec8Eeeu0xXdbba9frFj0=OqFfea0dXdd9vqai=hGuQ8kuc9pgc9s8qqaq=dirpe0xb9q8qiLsFr0=vr0=vr0dc8meaabaqaciaacaGaaeqabaqabeGadaaakeaadaWcaaqaamaapedabaacciGae8NXdyMaei4waSLaeiikaGIaemyDauNaeyOeI0IafmiEaGNbaKaadaWgaaWcbaGaemyAaKMaemOAaOgabeaakiabcMcaPiabc+caViab=n8aZnaaBaaaleaacqWGPbqAcqWGQbGAaeqaaOGaeiyxa0LaemizaqMaemyDauhaleaacqWG1bqDdaWgaaadbaGaemyBa0gabeaaaSqaaiabdwha1naaBaaameaacqWGTbqBcqGHRaWkcqaIXaqmaeqaaaqdcqGHRiI8aaGcbaWaa8qmaeaacqWFgpGzcqGGBbWwcqGGOaakcqWG1bqDcqGHsislcuWG4baEgaqcamaaBaaaleaacqWGPbqAcqWGQbGAaeqaaOGaeiykaKIaei4la8Iae83Wdm3aaSbaaSqaaiabdMgaPjabdQgaQbqabaGccqGGDbqxcqWGKbazcqWG1bqDaSqaaiabicdaWaqaaiabdYeambqdcqGHRiI8aaaaaaa@653C@ where *φ*[*u*]is the density function of a centered normalized Gaussian distribution, *m *is the index of the marker interval, *u*_*m *_and *u*_*m*+1 _are the absolute positions of the flanking markers on the original map of total length *L*. If the LOD score profile is available, a more accurate strategy can be applied by substituting *φ *for the density function which best fits the profile.

#### The meta-analysis model

The purpose of the QTL meta-analysis is to evaluate, for a given trait, the degree of congruency of the QTL detected in the *n *mapping experiments. By assuming that there is a finite number of true QTL locations, [17] proposed a clustering based approach to both classify the observed QTL and estimate the positions of the underlying QTL. Their method proceeds by testing all the possible QTL combinations and then choosing the one which maximizes a penalized log-likelihood. Although interesting, this method suffers from a categorical repartition of the QTL in the clusters, which is a limit case of Gaussian mixture models. We propose to adopt a similar clustering strategy but with a more standard Gaussian mixture model which allows QTL to be probabilistically distributed into clusters.

In order to lighten the notation we denote by *q *the total number of observed QTL locations and we ignore the mapping experiment subscripts so that X^
 MathType@MTEF@5@5@+=feaafiart1ev1aaatCvAUfKttLearuWrP9MDH5MBPbIqV92AaeXatLxBI9gBaebbnrfifHhDYfgasaacH8akY=wiFfYdH8Gipec8Eeeu0xXdbba9frFj0=OqFfea0dXdd9vqai=hGuQ8kuc9pgc9s8qqaq=dirpe0xb9q8qiLsFr0=vr0=vr0dc8meaabaqaciaacaGaaeqabaqabeGadaaakeaacuWGybawgaqcaaaa@2DF5@ = (x^
 MathType@MTEF@5@5@+=feaafiart1ev1aaatCvAUfKttLearuWrP9MDH5MBPbIqV92AaeXatLxBI9gBaebbnrfifHhDYfgasaacH8akY=wiFfYdH8Gipec8Eeeu0xXdbba9frFj0=OqFfea0dXdd9vqai=hGuQ8kuc9pgc9s8qqaq=dirpe0xb9q8qiLsFr0=vr0=vr0dc8meaabaqaciaacaGaaeqabaqabeGadaaakeaacuWG4baEgaqcaaaa@2E35@_1_,..., x^
 MathType@MTEF@5@5@+=feaafiart1ev1aaatCvAUfKttLearuWrP9MDH5MBPbIqV92AaeXatLxBI9gBaebbnrfifHhDYfgasaacH8akY=wiFfYdH8Gipec8Eeeu0xXdbba9frFj0=OqFfea0dXdd9vqai=hGuQ8kuc9pgc9s8qqaq=dirpe0xb9q8qiLsFr0=vr0=vr0dc8meaabaqaciaacaGaaeqabaqabeGadaaakeaacuWG4baEgaqcaaaa@2E35@_*q*_) and Σ = (*σ*_1_, ...,*σ*_*q*_). Then, let's suppose there are *K *≥ 1 true QTL located at X[K]=(x1[K],...,xK[K])
 MathType@MTEF@5@5@+=feaafiart1ev1aaatCvAUfKttLearuWrP9MDH5MBPbIqV92AaeXatLxBI9gBaebbnrfifHhDYfgasaacH8akY=wiFfYdH8Gipec8Eeeu0xXdbba9frFj0=OqFfea0dXdd9vqai=hGuQ8kuc9pgc9s8qqaq=dirpe0xb9q8qiLsFr0=vr0=vr0dc8meaabaqaciaacaGaaeqabaqabeGadaaakeaacqWGybawdaahaaWcbeqaaiabcUfaBjabdUealjabc2faDbaakiabg2da9iabcIcaOiabdIha4naaDaaaleaacqaIXaqmaeaacqGGBbWwcqWGlbWscqGGDbqxaaGccqGGSaalcqGGUaGlcqGGUaGlcqGGUaGlcqGGSaalcqWG4baEdaqhaaWcbaGaem4saSeabaGaei4waSLaem4saSKaeiyxa0faaOGaeiykaKcaaa@458C@ which segregate in at least one of the *n *QTL mapping experiments. Since the QTL position estimates X^
 MathType@MTEF@5@5@+=feaafiart1ev1aaatCvAUfKttLearuWrP9MDH5MBPbIqV92AaeXatLxBI9gBaebbnrfifHhDYfgasaacH8akY=wiFfYdH8Gipec8Eeeu0xXdbba9frFj0=OqFfea0dXdd9vqai=hGuQ8kuc9pgc9s8qqaq=dirpe0xb9q8qiLsFr0=vr0=vr0dc8meaabaqaciaacaGaaeqabaqabeGadaaakeaacuWGybawgaqcaaaa@2DF5@ are normally distributed around their true positions, the problem of finding the *K *underlying true positions can be viewed as a Gaussian mixture problem where the variances of each observation are known. Thus the log-likelihood of the observations can be written as follows:

L(X^,Σ;Θ[K])∝∑i=1qlog⁡[∑j=1Kπj[K]φ[(x^i−xj[K])/σi]]     (1)
 MathType@MTEF@5@5@+=feaafiart1ev1aaatCvAUfKttLearuWrP9MDH5MBPbIqV92AaeXatLxBI9gBaebbnrfifHhDYfgasaacH8akY=wiFfYdH8Gipec8Eeeu0xXdbba9frFj0=OqFfea0dXdd9vqai=hGuQ8kuc9pgc9s8qqaq=dirpe0xb9q8qiLsFr0=vr0=vr0dc8meaabaqaciaacaGaaeqabaqabeGadaaakeaacqWGmbatcqGGOaakcuWGybawgaqcaiabcYcaSiabfo6atjabcUda7iabfI5arnaaCaaaleqabaGaei4waSLaem4saSKaeiyxa0faaOGaeiykaKIaeyyhIu7aaabCaeaacyGGSbaBcqGGVbWBcqGGNbWzaSqaaiabdMgaPjabg2da9iabigdaXaqaaiabdghaXbqdcqGHris5aOWaamWaaeaadaaeWbqaaGGaciab=b8aWnaaDaaaleaacqWGQbGAaeaacqGGBbWwcqWGlbWscqGGDbqxaaGccqWFgpGzdaWadaqaaiabcIcaOiqbdIha4zaajaWaaSbaaSqaaiabdMgaPbqabaGccqGHsislcqWG4baEdaqhaaWcbaGaemOAaOgabaGaei4waSLaem4saSKaeiyxa0faaOGaeiykaKIaei4la8Iae83Wdm3aaSbaaSqaaiabdMgaPbqabaaakiaawUfacaGLDbaaaSqaaiabdQgaQjabg2da9iabigdaXaqaaiabdUealbqdcqGHris5aaGccaGLBbGaayzxaaGaaCzcaiaaxMaadaqadaqaaiabigdaXaGaayjkaiaawMcaaaaa@6DC1@

where Θ^[*K*] ^= (*X*^[*K*]^, Π^[*K*]^) denotes the parameters of the model, ∏[K]=(π1[K],...,πK[K])
 MathType@MTEF@5@5@+=feaafiart1ev1aaatCvAUfKttLearuWrP9MDH5MBPbIqV92AaeXatLxBI9gBaebbnrfifHhDYfgasaacH8akY=wiFfYdH8Gipec8Eeeu0xXdbba9frFj0=OqFfea0dXdd9vqai=hGuQ8kuc9pgc9s8qqaq=dirpe0xb9q8qiLsFr0=vr0=vr0dc8meaabaqaciaacaGaaeqabaqabeGadaaakeaacqGHpis1daahaaWcbeqaaiabcUfaBjabdUealjabc2faDbaakiabg2da9iabcIcaOGGaciab=b8aWnaaDaaaleaacqaIXaqmaeaacqGGBbWwcqWGlbWscqGGDbqxaaGccqGGSaalcqGGUaGlcqGGUaGlcqGGUaGlcqGGSaalcqWFapaCdaqhaaWcbaGaem4saSeabaGaei4waSLaem4saSKaeiyxa0faaOGaeiykaKcaaa@466F@ are the mixing proportions, which sum to one, and *φ*[*x*] is the density function of a centered normalized Gaussian distribution. We assume without loss of generality that x1[K]<x2[K]<...<xK[K]
 MathType@MTEF@5@5@+=feaafiart1ev1aaatCvAUfKttLearuWrP9MDH5MBPbIqV92AaeXatLxBI9gBaebbnrfifHhDYfgasaacH8akY=wiFfYdH8Gipec8Eeeu0xXdbba9frFj0=OqFfea0dXdd9vqai=hGuQ8kuc9pgc9s8qqaq=dirpe0xb9q8qiLsFr0=vr0=vr0dc8meaabaqaciaacaGaaeqabaqabeGadaaakeaacqWG4baEdaqhaaWcbaGaeGymaedabaGaei4waSLaem4saSKaeiyxa0faaOGaeyipaWJaemiEaG3aa0baaSqaaiabikdaYaqaaiabcUfaBjabdUealjabc2faDbaakiabgYda8iabc6caUiabc6caUiabc6caUiabgYda8iabdIha4naaDaaaleaacqWGlbWsaeaacqGGBbWwcqWGlbWscqGGDbqxaaaaaa@4548@ and that πj[K]
 MathType@MTEF@5@5@+=feaafiart1ev1aaatCvAUfKttLearuWrP9MDH5MBPbIqV92AaeXatLxBI9gBaebbnrfifHhDYfgasaacH8akY=wiFfYdH8Gipec8Eeeu0xXdbba9frFj0=OqFfea0dXdd9vqai=hGuQ8kuc9pgc9s8qqaq=dirpe0xb9q8qiLsFr0=vr0=vr0dc8meaabaqaciaacaGaaeqabaqabeGadaaakeaaiiGacqWFapaCdaqhaaWcbaGaemOAaOgabaGaei4waSLaem4saSKaeiyxa0faaaaa@3399@ ≠ 0, for *j *∈ [1,..., *K*]. In other word the distribution of the observed QTL locations is shaped by a mixture density where the components xj[K]
 MathType@MTEF@5@5@+=feaafiart1ev1aaatCvAUfKttLearuWrP9MDH5MBPbIqV92AaeXatLxBI9gBaebbnrfifHhDYfgasaacH8akY=wiFfYdH8Gipec8Eeeu0xXdbba9frFj0=OqFfea0dXdd9vqai=hGuQ8kuc9pgc9s8qqaq=dirpe0xb9q8qiLsFr0=vr0=vr0dc8meaabaqaciaacaGaaeqabaqabeGadaaakeaacqWG4baEdaqhaaWcbaGaemOAaOgabaGaei4waSLaem4saSKaeiyxa0faaaaa@334E@ are the positions of the true QTL on the linkage group and the mixing proportions *π*_*j *_represent the proportion of QTL related to the *j*^th ^true QTL which have been detected in the *n *mapping experiments.

Maximizing 1 can be achieved via a standard EM algorithm [34] by using the following parameter updates (M-step):

xj[K]=∑i=1qtij[K]x^iσi−2∑i=1qtij[K]σi−2 and πj[K]=1q∑i=1qtij[K]
 MathType@MTEF@5@5@+=feaafiart1ev1aaatCvAUfKttLearuWrP9MDH5MBPbIqV92AaeXatLxBI9gBaebbnrfifHhDYfgasaacH8akY=wiFfYdH8Gipec8Eeeu0xXdbba9frFj0=OqFfea0dXdd9vqai=hGuQ8kuc9pgc9s8qqaq=dirpe0xb9q8qiLsFr0=vr0=vr0dc8meaabaqaciaacaGaaeqabaqabeGadaaakeaacqWG4baEdaqhaaWcbaGaemOAaOgabaGaei4waSLaem4saSKaeiyxa0faaOGaeyypa0ZaaSaaaeaadaaeWbqaaiabdsha0naaDaaaleaacqWGPbqAcqWGQbGAaeaacqGGBbWwcqWGlbWscqGGDbqxaaGccuWG4baEgaqcamaaBaaaleaacqWGPbqAaeqaaGGacOGae83Wdm3aa0baaSqaaiabdMgaPbqaaiabgkHiTiabikdaYaaaaeaacqWGPbqAcqGH9aqpcqaIXaqmaeaacqWGXbqCa0GaeyyeIuoaaOqaamaaqahabaGaemiDaq3aa0baaSqaaiabdMgaPjabdQgaQbqaaiabcUfaBjabdUealjabc2faDbaakiab=n8aZnaaDaaaleaacqWGPbqAaeaacqGHsislcqaIYaGmaaaabaGaemyAaKMaeyypa0JaeGymaedabaGaemyCaehaniabggHiLdaaaOGaeeiiaaccbaGae4xyaeMae4NBa4Mae4hzaqMaeeiiaaIae8hWda3aa0baaSqaaiabdQgaQbqaaiabcUfaBjabdUealjabc2faDbaakiabg2da9maalaaabaGaeGymaedabaGaemyCaehaamaaqahabaGaemiDaq3aa0baaSqaaiabdMgaPjabdQgaQbqaaiabcUfaBjabdUealjabc2faDbaaaeaacqWGPbqAcqGH9aqpcqaIXaqmaeaacqWGXbqCa0GaeyyeIuoaaaa@7EA3@

where tij[K]
 MathType@MTEF@5@5@+=feaafiart1ev1aaatCvAUfKttLearuWrP9MDH5MBPbIqV92AaeXatLxBI9gBaebbnrfifHhDYfgasaacH8akY=wiFfYdH8Gipec8Eeeu0xXdbba9frFj0=OqFfea0dXdd9vqai=hGuQ8kuc9pgc9s8qqaq=dirpe0xb9q8qiLsFr0=vr0=vr0dc8meaabaqaciaacaGaaeqabaqabeGadaaakeaacqWG0baDdaqhaaWcbaGaemyAaKMaemOAaOgabaGaei4waSLaem4saSKaeiyxa0faaaaa@34A1@ is the conditional probability that x^
 MathType@MTEF@5@5@+=feaafiart1ev1aaatCvAUfKttLearuWrP9MDH5MBPbIqV92AaeXatLxBI9gBaebbnrfifHhDYfgasaacH8akY=wiFfYdH8Gipec8Eeeu0xXdbba9frFj0=OqFfea0dXdd9vqai=hGuQ8kuc9pgc9s8qqaq=dirpe0xb9q8qiLsFr0=vr0=vr0dc8meaabaqaciaacaGaaeqabaqabeGadaaakeaacuWG4baEgaqcaaaa@2E35@_*i *_belongs to the *j*^th ^meta-QTL. This conditional probability tij[K]
 MathType@MTEF@5@5@+=feaafiart1ev1aaatCvAUfKttLearuWrP9MDH5MBPbIqV92AaeXatLxBI9gBaebbnrfifHhDYfgasaacH8akY=wiFfYdH8Gipec8Eeeu0xXdbba9frFj0=OqFfea0dXdd9vqai=hGuQ8kuc9pgc9s8qqaq=dirpe0xb9q8qiLsFr0=vr0=vr0dc8meaabaqaciaacaGaaeqabaqabeGadaaakeaacqWG0baDdaqhaaWcbaGaemyAaKMaemOAaOgabaGaei4waSLaem4saSKaeiyxa0faaaaa@34A1@ is obtained by applying a simple Bayes' rule evaluated at the current parameter estimates (E-step):

tij[K]=πj[K]φ[(x^i−xj[K])/σi]∑j′=1Kπj′[K]φ[(x^i−xj′[K])/σi]
 MathType@MTEF@5@5@+=feaafiart1ev1aaatCvAUfKttLearuWrP9MDH5MBPbIqV92AaeXatLxBI9gBaebbnrfifHhDYfgasaacH8akY=wiFfYdH8Gipec8Eeeu0xXdbba9frFj0=OqFfea0dXdd9vqai=hGuQ8kuc9pgc9s8qqaq=dirpe0xb9q8qiLsFr0=vr0=vr0dc8meaabaqaciaacaGaaeqabaqabeGadaaakeaacqWG0baDdaqhaaWcbaGaemyAaKMaemOAaOgabaGaei4waSLaem4saSKaeiyxa0faaOGaeyypa0ZaaSaaaeaaiiGacqWFapaCdaqhaaWcbaGaemOAaOgabaGaei4waSLaem4saSKaeiyxa0faaOGae8NXdy2aamWaaeaacqGGOaakcuWG4baEgaqcamaaBaaaleaacqWGPbqAaeqaaOGaeyOeI0IaemiEaG3aa0baaSqaaiabdQgaQbqaaiabcUfaBjabdUealjabc2faDbaakiabcMcaPiabc+caViab=n8aZnaaBaaaleaacqWGPbqAaeqaaaGccaGLBbGaayzxaaaabaWaaabCaeaacqWFapaCdaqhaaWcbaGafmOAaOMbauaaaeaacqGGBbWwcqWGlbWscqGGDbqxaaGccqWFgpGzdaWadaqaaiabcIcaOiqbdIha4zaajaWaaSbaaSqaaiabdMgaPbqabaGccqGHsislcqWG4baEdaqhaaWcbaGafmOAaOMbauaaaeaacqGGBbWwcqWGlbWscqGGDbqxaaGccqGGPaqkcqGGVaWlcqWFdpWCdaWgaaWcbaGaemyAaKgabeaaaOGaay5waiaaw2faaaWcbaGafmOAaOMbauaacqGH9aqpcqaIXaqmaeaacqWGlbWsa0GaeyyeIuoaaaaaaa@72FB@

The EM-algorithm is run until reaching convergence: this yields the maximum-likelihood estimate denoted Θ˜
 MathType@MTEF@5@5@+=feaafiart1ev1aaatCvAUfKttLearuWrP9MDH5MBPbIqV92AaeXatLxBI9gBaebbnrfifHhDYfgasaacH8akY=wiFfYdH8Gipec8Eeeu0xXdbba9frFj0=OqFfea0dXdd9vqai=hGuQ8kuc9pgc9s8qqaq=dirpe0xb9q8qiLsFr0=vr0=vr0dc8meaabaqaciaacaGaaeqabaqabeGadaaakeaacuqHyoqugaacaaaa@2E32@^[*K*] ^= (X˜
 MathType@MTEF@5@5@+=feaafiart1ev1aaatCvAUfKttLearuWrP9MDH5MBPbIqV92AaeXatLxBI9gBaebbnrfifHhDYfgasaacH8akY=wiFfYdH8Gipec8Eeeu0xXdbba9frFj0=OqFfea0dXdd9vqai=hGuQ8kuc9pgc9s8qqaq=dirpe0xb9q8qiLsFr0=vr0=vr0dc8meaabaqaciaacaGaaeqabaqabeGadaaakeaacuWGybawgaacaaaa@2DF4@^[*K*]^, ∏˜
 MathType@MTEF@5@5@+=feaafiart1ev1aaatCvAUfKttLearuWrP9MDH5MBPbIqV92AaeXatLxBI9gBaebbnrfifHhDYfgasaacH8akY=wiFfYdH8Gipec8Eeeu0xXdbba9frFj0=OqFfea0dXdd9vqai=hGuQ8kuc9pgc9s8qqaq=dirpe0xb9q8qiLsFr0=vr0=vr0dc8meaabaqaciaacaGaaeqabaqabeGadaaakeaacuGHpis1gaacaaaa@2E4D@^[*K*]^). Finally, once Θ˜
 MathType@MTEF@5@5@+=feaafiart1ev1aaatCvAUfKttLearuWrP9MDH5MBPbIqV92AaeXatLxBI9gBaebbnrfifHhDYfgasaacH8akY=wiFfYdH8Gipec8Eeeu0xXdbba9frFj0=OqFfea0dXdd9vqai=hGuQ8kuc9pgc9s8qqaq=dirpe0xb9q8qiLsFr0=vr0=vr0dc8meaabaqaciaacaGaaeqabaqabeGadaaakeaacuqHyoqugaacaaaa@2E32@^[*K*] ^has been obtained the variance-covariance matrix of the parameter estimates, conditionally to the current model, can be computed by applying the Supplemental EM (SEM) strategy proposed by [35].

The problem is that we do not know *K*, i.e the number of true QTL positions. Since the mixture model of *K *components is nested into the model with *K *+ 1 components, the likelihood ratio test (LRT) should be suitable. However, as discussed by many authors (see for instance [36,37]) the LRT statistic does not follow the usual *χ*^2 ^distribution due to testing a null hypothesis on the boundary of the parameter space (i.e the regularity conditions on the loglikelihood do not hold). Another strategy is to use the Kullback-Leibler information in order to derive the information criterion which is widely used to select a statistical model. In particular, the Kullback-Leibler information can be viewed as a measure of goodness-of-fit of a statistical model. Here for a given value of *K*, minimizing the Kullback-Leibler information is equivalent to maximizing the negentropy KL,

KL=−∫X^g(X^,Σ)L(X^,Σ;Θ[K])dX^
 MathType@MTEF@5@5@+=feaafiart1ev1aaatCvAUfKttLearuWrP9MDH5MBPbIqV92AaeXatLxBI9gBaebbnrfifHhDYfgasaacH8akY=wiFfYdH8Gipec8Eeeu0xXdbba9frFj0=OqFfea0dXdd9vqai=hGuQ8kuc9pgc9s8qqaq=dirpe0xb9q8qiLsFr0=vr0=vr0dc8meaabaqaciaacaGaaeqabaqabeGadaaakeaaieaacqWFlbWscqWFmbatcqGH9aqpcqGHsisldaWdraqaaiabdEgaNjabcIcaOaWcbaGafmiwaGLbaKaaaeqaniabgUIiYdGccuWGybawgaqcaiabcYcaSiabfo6atjabcMcaPiabdYeamjabcIcaOiqbdIfayzaajaGaeiilaWIaeu4OdmLaei4oaSJaeuiMde1aaWbaaSqabeaacqGGBbWwcqWGlbWscqGGDbqxaaGccqGGPaqkcqWGKbazcuWGybawgaqcaaaa@4A58@

where *g*(X^
 MathType@MTEF@5@5@+=feaafiart1ev1aaatCvAUfKttLearuWrP9MDH5MBPbIqV92AaeXatLxBI9gBaebbnrfifHhDYfgasaacH8akY=wiFfYdH8Gipec8Eeeu0xXdbba9frFj0=OqFfea0dXdd9vqai=hGuQ8kuc9pgc9s8qqaq=dirpe0xb9q8qiLsFr0=vr0=vr0dc8meaabaqaciaacaGaaeqabaqabeGadaaakeaacuWGybawgaqcaaaa@2DF5@, Σ) is the the true underlying density function. Thus, from the point of view of the negentropy maximization principle, the goodness of the model can be evaluated by the expected log-likelihood. Note that the negentropy maximization principle naturally leads to the maximization of the log-likelihood. However, the maximized log-likelihood is a naive estimate of the expected loglikelihood: since the same data set X^
 MathType@MTEF@5@5@+=feaafiart1ev1aaatCvAUfKttLearuWrP9MDH5MBPbIqV92AaeXatLxBI9gBaebbnrfifHhDYfgasaacH8akY=wiFfYdH8Gipec8Eeeu0xXdbba9frFj0=OqFfea0dXdd9vqai=hGuQ8kuc9pgc9s8qqaq=dirpe0xb9q8qiLsFr0=vr0=vr0dc8meaabaqaciaacaGaaeqabaqabeGadaaakeaacuWGybawgaqcaaaa@2DF5@ is used for both the estimation of the parameter and the estimation of the expected log-likelihood, *L*(X^
 MathType@MTEF@5@5@+=feaafiart1ev1aaatCvAUfKttLearuWrP9MDH5MBPbIqV92AaeXatLxBI9gBaebbnrfifHhDYfgasaacH8akY=wiFfYdH8Gipec8Eeeu0xXdbba9frFj0=OqFfea0dXdd9vqai=hGuQ8kuc9pgc9s8qqaq=dirpe0xb9q8qiLsFr0=vr0=vr0dc8meaabaqaciaacaGaaeqabaqabeGadaaakeaacuWGybawgaqcaaaa@2DF5@, Σ; Θ^[*K*]^) is a biased estimator of the expected loglikelihood. Its bias is defined by,

B=EX^[L(X^,Σ;Θ˜[K])−EY[L(Y,Σ;Θ˜[K])]]
 MathType@MTEF@5@5@+=feaafiart1ev1aaatCvAUfKttLearuWrP9MDH5MBPbIqV92AaeXatLxBI9gBaebbnrfifHhDYfgasaacH8akY=wiFfYdH8Gipec8Eeeu0xXdbba9frFj0=OqFfea0dXdd9vqai=hGuQ8kuc9pgc9s8qqaq=dirpe0xb9q8qiLsFr0=vr0=vr0dc8meaabaqaciaacaGaaeqabaqabeGadaaakeaacqWGcbGqcqGH9aqpcqWGfbqrdaWgaaWcbaGafmiwaGLbaKaaaeqaaOWaamWaaeaacqWGmbatcqGGOaakcuWGybawgaqcaiabcYcaSiabfo6atjabcUda7iqbfI5arzaaiaWaaWbaaSqabeaacqGGBbWwcqWGlbWscqGGDbqxaaGccqGGPaqkcqGHsislcqWGfbqrdaWgaaWcbaGaemywaKfabeaakmaadmaabaGaemitaWKaeiikaGIaemywaKLaeiilaWIaeu4OdmLaei4oaSJafuiMdeLbaGaadaahaaWcbeqaaiabcUfaBjabdUealjabc2faDbaakiabcMcaPaGaay5waiaaw2faaaGaay5waiaaw2faaaaa@524C@

and the use of *L*(X^
 MathType@MTEF@5@5@+=feaafiart1ev1aaatCvAUfKttLearuWrP9MDH5MBPbIqV92AaeXatLxBI9gBaebbnrfifHhDYfgasaacH8akY=wiFfYdH8Gipec8Eeeu0xXdbba9frFj0=OqFfea0dXdd9vqai=hGuQ8kuc9pgc9s8qqaq=dirpe0xb9q8qiLsFr0=vr0=vr0dc8meaabaqaciaacaGaaeqabaqabeGadaaakeaacuWGybawgaqcaaaa@2DF5@, Σ; Θ˜
 MathType@MTEF@5@5@+=feaafiart1ev1aaatCvAUfKttLearuWrP9MDH5MBPbIqV92AaeXatLxBI9gBaebbnrfifHhDYfgasaacH8akY=wiFfYdH8Gipec8Eeeu0xXdbba9frFj0=OqFfea0dXdd9vqai=hGuQ8kuc9pgc9s8qqaq=dirpe0xb9q8qiLsFr0=vr0=vr0dc8meaabaqaciaacaGaaeqabaqabeGadaaakeaacuqHyoqugaacaaaa@2E32@^[*K*]^) - *B *is justified as an estimate of KL. There are different strategies to estimate this bias and several information based criteria have been reported in the mixture model literature in order to tackle the issue raised by choosing the number of components (see Additional File 2). In the next *Simulation section*, we propose to evaluate the ability of some of these information based criteria to determine the optimal number of QTL.

### Simulation study

For the sake of concision, in this section we only present simulations for the QTL meta-analysis (simulations for the meta-analysis of genetic maps are described in Additional File 3). We assume that the complexity which shapes the distribution of the observed QTL along the chromosome can be represented by our mixture model. In order to explore mixture configurations which are realistic we have assumed that the QTL effects have a L-shaped distribution (i.e most of the detected QTL in mapping experiments have a small effect and only a few show a strong effect, or in other words, most of the detected QTL have large confidence intervals). Consequently this implies that Σ^-1^, the inverse of the QTL standard deviations, has also a L-shaped distribution (i.e the smaller the effect of the QTL the larger the confidence interval of the estimated QTL position). Then for a given value of the number of true QTL, *K*, we randomly generated configurations as follows:

1. Draw Σ from a inverse gamma distribution (this simply mimics a L-shaped distribution).

2. Generate the mixing proportions by choosing them over the discrete uniform [0.1, 0.9] distribution subject to constraint ∑k=1Kπk=1
 MathType@MTEF@5@5@+=feaafiart1ev1aaatCvAUfKttLearuWrP9MDH5MBPbIqV92AaeXatLxBI9gBaebbnrfifHhDYfgasaacH8akY=wiFfYdH8Gipec8Eeeu0xXdbba9frFj0=OqFfea0dXdd9vqai=hGuQ8kuc9pgc9s8qqaq=dirpe0xb9q8qiLsFr0=vr0=vr0dc8meaabaqaciaacaGaaeqabaqabeGadaaakeaadaaeWbqaaGGaciab=b8aWnaaBaaaleaacqWGRbWAaeqaaaqaaiabdUgaRjabg2da9iabigdaXaqaaiabdUealbqdcqGHris5aOGaeyypa0JaeGymaedaaa@38A6@.

3. Draw from a multinomial, with frequencies equal to the mixing proportions, the origins of the *q *observed QTL *Z *= (*z*_1_,...,*z*_*q*_) where *z*_*ij *_= 1 if the *i*^th ^observed QTL belongs to the *j*^th ^true QTL, 0 otherwise.

4. Generate the true QTL positions, *X *= (*x*_1_,..., *x*_*k*_), subject to constraint *x*_*k *_+ *τ*_*min *_<*x*_*k*+1 _<*x*_*k *_+ *τ*_*max *_where *τ*_*min *_and *τ*_*max *_are defined so that the distance between *x*_*k *_and *x*_*k*+1 _lies between *δ*_*min *_and *δ*_*max*_. The distance *δ *is defined as the mahalanobis distance between *x*_*k *_and *x*_*k*+1_: δ=(xk−xk+1)2ak2+ak+12
 MathType@MTEF@5@5@+=feaafiart1ev1aaatCvAUfKttLearuWrP9MDH5MBPbIqV92AaeXatLxBI9gBaebbnrfifHhDYfgasaacH8akY=wiFfYdH8Gipec8Eeeu0xXdbba9frFj0=OqFfea0dXdd9vqai=hGuQ8kuc9pgc9s8qqaq=dirpe0xb9q8qiLsFr0=vr0=vr0dc8meaabaqaciaacaGaaeqabaqabeGadaaakeaaiiGacqWF0oazcqGH9aqpdaGcaaqaamaalaaabaGaeiikaGIaemiEaG3aaSbaaSqaaiabdUgaRbqabaGccqGHsislcqWG4baEdaWgaaWcbaGaem4AaSMaey4kaSIaeGymaedabeaakiabcMcaPmaaCaaaleqabaGaeGOmaidaaaGcbaGaemyyae2aa0baaSqaaiabdUgaRbqaaiabikdaYaaakiabgUcaRiabdggaHnaaDaaaleaacqWGRbWAcqGHRaWkcqaIXaqmaeaacqaIYaGmaaaaaaqabaaaaa@4584@ where ak=(∑i=1qzikσi)/(∑i=1qzik)
 MathType@MTEF@5@5@+=feaafiart1ev1aaatCvAUfKttLearuWrP9MDH5MBPbIqV92AaeXatLxBI9gBaebbnrfifHhDYfgasaacH8akY=wiFfYdH8Gipec8Eeeu0xXdbba9frFj0=OqFfea0dXdd9vqai=hGuQ8kuc9pgc9s8qqaq=dirpe0xb9q8qiLsFr0=vr0=vr0dc8meaabaqaciaacaGaaeqabaqabeGadaaakeaacqWGHbqydaWgaaWcbaGaem4AaSgabeaakiabg2da9iabcIcaOmaaqahabaGaemOEaO3aaSbaaSqaaiabdMgaPjabdUgaRbqabaacciGccqWFdpWCdaWgaaWcbaGaemyAaKgabeaaaeaacqWGPbqAcqGH9aqpcqaIXaqmaeaacqWGXbqCa0GaeyyeIuoakiabcMcaPiabc+caViabcIcaOmaaqahabaGaemOEaO3aaSbaaSqaaiabdMgaPjabdUgaRbqabaaabaGaemyAaKMaeyypa0JaeGymaedabaGaemyCaehaniabggHiLdGccqGGPaqkaaa@4EF7@ is the average standard deviation for the *k*^th ^true QTL. This measures the separation between consecutive true QTL relatively to the precision of the experiments: *δ *≤ 2 corresponds to tightly or moderately separated QTL, while *δ *≥ 3 corresponds to well separated QTL.

We stress that this process is not an attempt to describe reality, nevertheless it makes it possible to cover a large range of possible repartitions of the QTL. Finally, for each of the 4 distance constraints considered (*δ*_*min *_= 1, 2, 3,4 and *δ*_*max *_= *δ*_*min *_+ 1), 50 configurations were generated. For a given value of *K*, the following scenario was repeated 100 times:

1. Draw a sample X^
 MathType@MTEF@5@5@+=feaafiart1ev1aaatCvAUfKttLearuWrP9MDH5MBPbIqV92AaeXatLxBI9gBaebbnrfifHhDYfgasaacH8akY=wiFfYdH8Gipec8Eeeu0xXdbba9frFj0=OqFfea0dXdd9vqai=hGuQ8kuc9pgc9s8qqaq=dirpe0xb9q8qiLsFr0=vr0=vr0dc8meaabaqaciaacaGaaeqabaqabeGadaaakeaacuWGybawgaqcaaaa@2DF5@ of size *q*.

2. Run the EM-algorithm to obtain Θ˜
 MathType@MTEF@5@5@+=feaafiart1ev1aaatCvAUfKttLearuWrP9MDH5MBPbIqV92AaeXatLxBI9gBaebbnrfifHhDYfgasaacH8akY=wiFfYdH8Gipec8Eeeu0xXdbba9frFj0=OqFfea0dXdd9vqai=hGuQ8kuc9pgc9s8qqaq=dirpe0xb9q8qiLsFr0=vr0=vr0dc8meaabaqaciaacaGaaeqabaqabeGadaaakeaacuqHyoqugaacaaaa@2E32@^[*K*] ^for *K *= 1,..., *q*.

3. Choose the best model according to each criterion.

Since the goal of QTL meta-analysis is to obtain a better predictive inference of the true QTL locations we have compared the two alternative strategies:

• Strategy 1 : choose the model with as many true QTL as the number of observed QTL. It is the naive model, $\bar{x}$_*i*_(1) = x^
 MathType@MTEF@5@5@+=feaafiart1ev1aaatCvAUfKttLearuWrP9MDH5MBPbIqV92AaeXatLxBI9gBaebbnrfifHhDYfgasaacH8akY=wiFfYdH8Gipec8Eeeu0xXdbba9frFj0=OqFfea0dXdd9vqai=hGuQ8kuc9pgc9s8qqaq=dirpe0xb9q8qiLsFr0=vr0=vr0dc8meaabaqaciaacaGaaeqabaqabeGadaaakeaacuWG4baEgaqcaaaa@2E35@_*i*_

• Strategy 2 : choose the best model *K *according to the model choice criterion, x¯i(2)=∑j=1Ktij[K]x˜j[K]
 MathType@MTEF@5@5@+=feaafiart1ev1aaatCvAUfKttLearuWrP9MDH5MBPbIqV92AaeXatLxBI9gBaebbnrfifHhDYfgasaacH8akY=wiFfYdH8Gipec8Eeeu0xXdbba9frFj0=OqFfea0dXdd9vqai=hGuQ8kuc9pgc9s8qqaq=dirpe0xb9q8qiLsFr0=vr0=vr0dc8meaabaqaciaacaGaaeqabaqabeGadaaakeaacuWG4baEgaqeamaaBaaaleaacqWGPbqAaeqaaOGaeiikaGIaeGOmaiJaeiykaKIaeyypa0ZaaabmaeaacqWG0baDdaqhaaWcbaGaemyAaKMaemOAaOgabaGaei4waSLaem4saSKaeiyxa0faaaqaaiabdQgaQjabg2da9iabigdaXaqaaiabdUealbqdcqGHris5aOGafmiEaGNbaGaadaqhaaWcbaGaemOAaOgabaGaei4waSLaem4saSKaeiyxa0faaaaa@4891@

For each strategy *s *= 1, 2, the measure of performance used was the mean squared error of prediction defined as follows:

MSEP(s)=1q∑i=1qE[(xi−x¯i(s))2]
 MathType@MTEF@5@5@+=feaafiart1ev1aaatCvAUfKttLearuWrP9MDH5MBPbIqV92AaeXatLxBI9gBaebbnrfifHhDYfgasaacH8akY=wiFfYdH8Gipec8Eeeu0xXdbba9frFj0=OqFfea0dXdd9vqai=hGuQ8kuc9pgc9s8qqaq=dirpe0xb9q8qiLsFr0=vr0=vr0dc8meaabaqaciaacaGaaeqabaqabeGadaaakeaaieaacqWFnbqtcqWFtbWucqWFfbqrcqWFqbaucqGGOaakcqWGZbWCcqGGPaqkcqGH9aqpdaWcaaqaaiabigdaXaqaaiabdghaXbaadaaeWbqaaiabdweafjabcUfaBjabcIcaOiabdIha4naaBaaaleaacqWGPbqAaeqaaOGaeyOeI0caleaacqWGPbqAcqGH9aqpcqaIXaqmaeaacqWGXbqCa0GaeyyeIuoakiqbdIha4zaaraWaaSbaaSqaaiabdMgaPbqabaGccqGGOaakcqWGZbWCcqGGPaqkcqGGPaqkdaahaaWcbeqaaiabikdaYaaakiabc2faDbaa@4F75@

Absolute values of these MSEP are not of interest here because our goal is comparison of strategies; hence, we consider the ratios MSEP(2)/MSEP(1) for 5 different information based criteria:

• AIC = -2*L*(X^
 MathType@MTEF@5@5@+=feaafiart1ev1aaatCvAUfKttLearuWrP9MDH5MBPbIqV92AaeXatLxBI9gBaebbnrfifHhDYfgasaacH8akY=wiFfYdH8Gipec8Eeeu0xXdbba9frFj0=OqFfea0dXdd9vqai=hGuQ8kuc9pgc9s8qqaq=dirpe0xb9q8qiLsFr0=vr0=vr0dc8meaabaqaciaacaGaaeqabaqabeGadaaakeaacuWGybawgaqcaaaa@2DF5@, Σ; Θ˜
 MathType@MTEF@5@5@+=feaafiart1ev1aaatCvAUfKttLearuWrP9MDH5MBPbIqV92AaeXatLxBI9gBaebbnrfifHhDYfgasaacH8akY=wiFfYdH8Gipec8Eeeu0xXdbba9frFj0=OqFfea0dXdd9vqai=hGuQ8kuc9pgc9s8qqaq=dirpe0xb9q8qiLsFr0=vr0=vr0dc8meaabaqaciaacaGaaeqabaqabeGadaaakeaacuqHyoqugaacaaaa@2E32@^[*K*]^) + 2*ν*

• AIC_*c *_= -2*L*(X^
 MathType@MTEF@5@5@+=feaafiart1ev1aaatCvAUfKttLearuWrP9MDH5MBPbIqV92AaeXatLxBI9gBaebbnrfifHhDYfgasaacH8akY=wiFfYdH8Gipec8Eeeu0xXdbba9frFj0=OqFfea0dXdd9vqai=hGuQ8kuc9pgc9s8qqaq=dirpe0xb9q8qiLsFr0=vr0=vr0dc8meaabaqaciaacaGaaeqabaqabeGadaaakeaacuWGybawgaqcaaaa@2DF5@, Σ; Θ˜
 MathType@MTEF@5@5@+=feaafiart1ev1aaatCvAUfKttLearuWrP9MDH5MBPbIqV92AaeXatLxBI9gBaebbnrfifHhDYfgasaacH8akY=wiFfYdH8Gipec8Eeeu0xXdbba9frFj0=OqFfea0dXdd9vqai=hGuQ8kuc9pgc9s8qqaq=dirpe0xb9q8qiLsFr0=vr0=vr0dc8meaabaqaciaacaGaaeqabaqabeGadaaakeaacuqHyoqugaacaaaa@2E32@^[*K*]^) + 2*ν *+ 2ν(ν+1)q−ν−1
 MathType@MTEF@5@5@+=feaafiart1ev1aaatCvAUfKttLearuWrP9MDH5MBPbIqV92AaeXatLxBI9gBaebbnrfifHhDYfgasaacH8akY=wiFfYdH8Gipec8Eeeu0xXdbba9frFj0=OqFfea0dXdd9vqai=hGuQ8kuc9pgc9s8qqaq=dirpe0xb9q8qiLsFr0=vr0=vr0dc8meaabaqaciaacaGaaeqabaqabeGadaaakeaadaWcaaqaaiabikdaYGGaciab=17aUjabcIcaOiab=17aUjabgUcaRiabigdaXiabcMcaPaqaaiabdghaXjabgkHiTiab=17aUjabgkHiTiabigdaXaaaaaa@3A8C@

• AIC3 = -2*L*(X^
 MathType@MTEF@5@5@+=feaafiart1ev1aaatCvAUfKttLearuWrP9MDH5MBPbIqV92AaeXatLxBI9gBaebbnrfifHhDYfgasaacH8akY=wiFfYdH8Gipec8Eeeu0xXdbba9frFj0=OqFfea0dXdd9vqai=hGuQ8kuc9pgc9s8qqaq=dirpe0xb9q8qiLsFr0=vr0=vr0dc8meaabaqaciaacaGaaeqabaqabeGadaaakeaacuWGybawgaqcaaaa@2DF5@, Σ; Θ˜
 MathType@MTEF@5@5@+=feaafiart1ev1aaatCvAUfKttLearuWrP9MDH5MBPbIqV92AaeXatLxBI9gBaebbnrfifHhDYfgasaacH8akY=wiFfYdH8Gipec8Eeeu0xXdbba9frFj0=OqFfea0dXdd9vqai=hGuQ8kuc9pgc9s8qqaq=dirpe0xb9q8qiLsFr0=vr0=vr0dc8meaabaqaciaacaGaaeqabaqabeGadaaakeaacuqHyoqugaacaaaa@2E32@^[*K*]^) + 3*ν*

• BIC = -2*L*(X^
 MathType@MTEF@5@5@+=feaafiart1ev1aaatCvAUfKttLearuWrP9MDH5MBPbIqV92AaeXatLxBI9gBaebbnrfifHhDYfgasaacH8akY=wiFfYdH8Gipec8Eeeu0xXdbba9frFj0=OqFfea0dXdd9vqai=hGuQ8kuc9pgc9s8qqaq=dirpe0xb9q8qiLsFr0=vr0=vr0dc8meaabaqaciaacaGaaeqabaqabeGadaaakeaacuWGybawgaqcaaaa@2DF5@, Σ; Θ) + *ν *log(*q*)

• EIC ≈ AIC - *K *+ 1, which was obtained by means of simulations (data not shown).

where *ν *= 2*K *- 1 is the number of free parameters of the model and *q *the number of observed QTL along the chromosome (see Additional file 2 for theoretical details on each above criterion).

**Figure 1:**
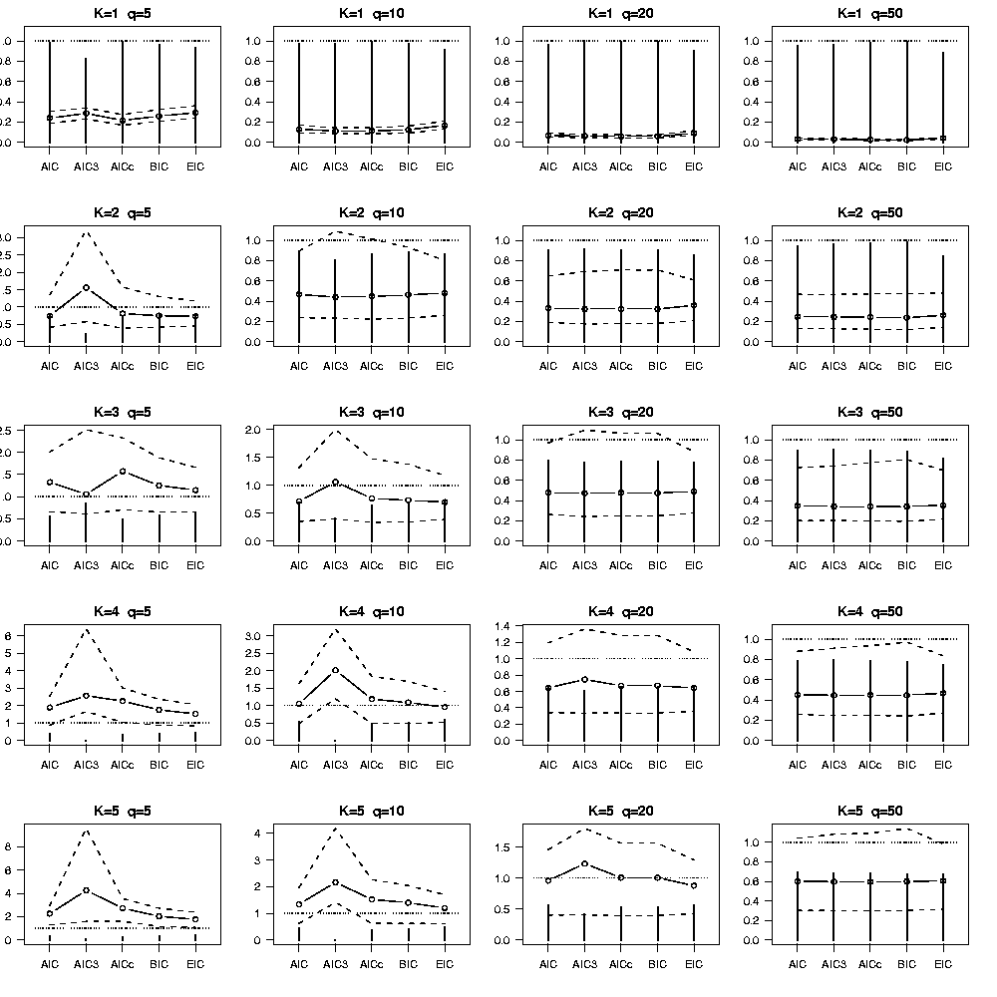
**Comparison of model choice criteria: a simulation study**. Simulation results for different values of the true number of QTL, *K*, and the number of observed QTL, *q*. The vertical bars indicate the probability that the best model selected by the criterion is the true model. The open circles, respectively the dotted lines, represent the mean, respectively the 0.1% and 0.9% quantiles, of the ratios MSEP(2)/MSEP(1) for each criterion.

**Figure 2:**
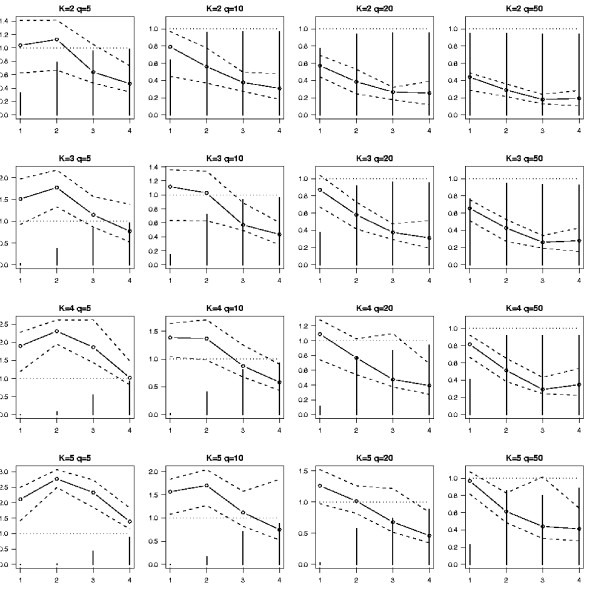
**Performance of AIC: a simulation study**. Behavior of the AIC criterion for the 4 distance constraints, *δ*_min _= 1, 2, 3, 4, and for different values of the true number of QTL, *K*, and the number of observed QTL, *q*. The vertical bars indicate the probability than the AIC criterion has selected the true model. The open circles, respectively the dotted lines, represent the mean, respectively the 0.1% and 0.9% quantiles, of the ratios MSEP(2)/MSEP(1).

In Figure 1 we summarized the result of simulations for several values of *K *and *q *by averaging over the distance constraint configurations (*δ*_min _= 1, 2, 3 and 4). At first sight the 5 criteria seem to have the same behavior whatever the configuration, except for AIC3 which crucially underperforms for small values of *q *(this can be explained by the higher penality of AIC3 comparing to the other criteria for small values of *q*). For reasonable sample size relatively to the true number of components the meta-analysis appears to be more efficient than strategy 1. Since the AIC criterion has relatively good performance in these simulations we assume that there is no need for a specific theory to deal with this kind of mixture models and that this criterion can be used to carry out model selection in this context. So, in Figure 2 we focus on the AIC criterion for the different distance configurations *δ*_min _= 1, 2, 3 and 4. This clearly shows that, for configurations with reasonable separation between the true positions of the QTL, the meta-analysis performs relatively well. It is worth noting that the better the probability to choose the true model, the better the quality of the QTL position estimates. In order to evaluate the ability of the meta-analysis to improve the precision on the "true" QTL locations we computed the quantities |*x*_*i *_- x^
 MathType@MTEF@5@5@+=feaafiart1ev1aaatCvAUfKttLearuWrP9MDH5MBPbIqV92AaeXatLxBI9gBaebbnrfifHhDYfgasaacH8akY=wiFfYdH8Gipec8Eeeu0xXdbba9frFj0=OqFfea0dXdd9vqai=hGuQ8kuc9pgc9s8qqaq=dirpe0xb9q8qiLsFr0=vr0=vr0dc8meaabaqaciaacaGaaeqabaqabeGadaaakeaacuWG4baEgaqcaaaa@2E35@_*i *_(*s*)| and calculated the quantiles at 95 and 90% of its empirical distribution over all the QTL for the two strategies. The smaller this confidence interval, the better the estimated position x^
 MathType@MTEF@5@5@+=feaafiart1ev1aaatCvAUfKttLearuWrP9MDH5MBPbIqV92AaeXatLxBI9gBaebbnrfifHhDYfgasaacH8akY=wiFfYdH8Gipec8Eeeu0xXdbba9frFj0=OqFfea0dXdd9vqai=hGuQ8kuc9pgc9s8qqaq=dirpe0xb9q8qiLsFr0=vr0=vr0dc8meaabaqaciaacaGaaeqabaqabeGadaaakeaacuWG4baEgaqcaaaa@2E35@_*i*_(*s*). We reported in Supplementary Table 1 (see Additional File 4) the average ratios of these quantities between the two strategies. Hence, if there are actually one, two, three or four different QTL locations with a reasonable separation (*δ*_*min *_≥ 2), we can see that the meta-analysis gives better estimates of the QTL locations and makes it possible to reduce the length of the 95% CI (in most of the situtations this length is halved). According to [29] to halve a CI in a QTL experiment, one needs to use at least two times the initial number of individuals.

**Table 1:** Model choice criteria for the meta-analysis of flowering time QTL on maize chromosome 8

K	Model Choice Criterion			
	
	AIC	AIC_*c*_	AIC3	BIC
1	1096.32	1088.94	1088.32	1084.11
2	497.22	490.52	491.22	488.06
3	139.15	133.79	135.15	133.05
4	34.73	31.53	32.73	31.67
**5**	**0.00**	**0.00**	**0.00**	**0.00**
6	4.00	8.50	6.00	7.05
7	8.00	18.70	12.00	14.11
8	12.00	31.17	18.00	21.16
9	16.00	46.75	24.00	28.21
10	19.54	66.33	29.54	34.81
34	51.42	43.92	76.42	89.58

All the criteria select the model with 5 QTL (in bold). The values are adjusted with respect to the minimal value of the criterion, i.e. the value for K = 5..

### Implementation

The previous methods have been implemented into a Java package called **MetaQTL**. This package includes also additional programs to format, organize or visualize the data. All the programs in **MetaQTL **are command line programs (see Supplementary Table 2 in Additional File 4 for a complete list of the programs available in the package). Each program performs a specific task and the programs can be combined by the user as a group to run a complete analysis. Thanks to its flexible and modular implementation, **MetaQTL **could also be integrated in more elaborated softwares if needed. First, before running meta-analysis one needs to store the different QTL studies into a database. To do this **MetaQTL **uses a simple multiple plain text files database. Each file corresponds to a table and the database is organized as follows:

**Table 2:** Parameter estimates of the best meta-analysis model for flowering time on maize chromosome 8

QTL	Position X˜ MathType@MTEF@5@5@+=feaafiart1ev1aaatCvAUfKttLearuWrP9MDH5MBPbIqV92AaeXatLxBI9gBaebbnrfifHhDYfgasaacH8akY=wiFfYdH8Gipec8Eeeu0xXdbba9frFj0=OqFfea0dXdd9vqai=hGuQ8kuc9pgc9s8qqaq=dirpe0xb9q8qiLsFr0=vr0=vr0dc8meaabaqaciaacaGaaeqabaqabeGadaaakeaacuWGybawgaacaaaa@2DF4@	Weight ∏˜ MathType@MTEF@5@5@+=feaafiart1ev1aaatCvAUfKttLearuWrP9MDH5MBPbIqV92AaeXatLxBI9gBaebbnrfifHhDYfgasaacH8akY=wiFfYdH8Gipec8Eeeu0xXdbba9frFj0=OqFfea0dXdd9vqai=hGuQ8kuc9pgc9s8qqaq=dirpe0xb9q8qiLsFr0=vr0=vr0dc8meaabaqaciaacaGaaeqabaqabeGadaaakeaacuGHpis1gaacaaaa@2E4D@	Mahalanobis distance to next QTL	95% CI
1	14.6	0.06	6.21	11.7
2	75.4	0.35	1.26	6.2
3	89.5	0.44	2.64	3.8
4	114.5	0.07	5.20	11.1
5	165.2	0.09	-	13.9

The positions and the lengths of the CI are given in cM. The 95% CI is derived from the conditional variance estimate of the position obtained by applying the SEM strategy. Note that QTL 2 and 3 correspond to *vgt2 *and *vgt1*, respectively (following the designation of [43]).

• Experiment file: stores descriptions on mapping experiments (name, population type and size, reference, ...).

• Genetic map directory : contains one file per input marker map. Each file contains the corresponding genetic marker map.

• QTL map directory : contains one file per QTL mapping experiments. For each QTL mapping experiment the file provides the properties of each detected QTL (trait, position, confidence interval, r-square, ...).

• Trait ontology file: describes how the traits are related together using a simple hierarchical relationship scheme. This information can then be used to group the QTL according to the ontology in subsequent analyses.

Once the database created, **MetaQTL **first checks the input data files and then summarizes their content into a set of XML files. All the programs of **MetaQTL **use these XML files as inputs. Utilities are provided to convert them in various plain text file formats if required (for more details on using **MetaQTL **see the user manual in Additional File 5).

### Application

Recently, [12] made a bibliographical review of QTL studies relative to 4 traits related to flowering time in maize: days to pollen shed (DPS), silking date (SD), plant height (HT) and leaf number (LN). From the 22 QTL studies they reported, we excluded 6 experiments for which QTL detection was based on ANOVA with a low density of markers and 2 other for which it was not possible to get exact information on either the genetic linkage map or the QTL locations. In addition to these 15 mapping experiments we considered 3 other recent experiments (details of these 18 QTL studies are given in Additional File 6). We focus here on chromosome 8 and we present results by using for each step of the meta-analysis the corresponding program name of **MetaQTL.**

#### Result of InfoMap

Among the 153 distinct markers which have been positioned over the 18 mapping experiments on the chromosome 8, only 53 markers are observed in at least two different mapping experiments. We restricted the meta-analysis to these 53 markers. Only one order inconsistency was detected between [38] and [39] concerning markers *umc89a *and *umc12a*. As in [38]*umc12a *is very close to *umc89a *(less than 2 cM) we have decided to ignore this marker in this mapping experiment. Over the 18 mapping experiments the mean interval distance was about 18.9 cM with an average of 8.7 markers per mapping experiment and it existed at least one common marker path which connected all the mapping experiments together (insuring that the WLS can be applied).

#### Result of ConsMap

**Figure 3:**
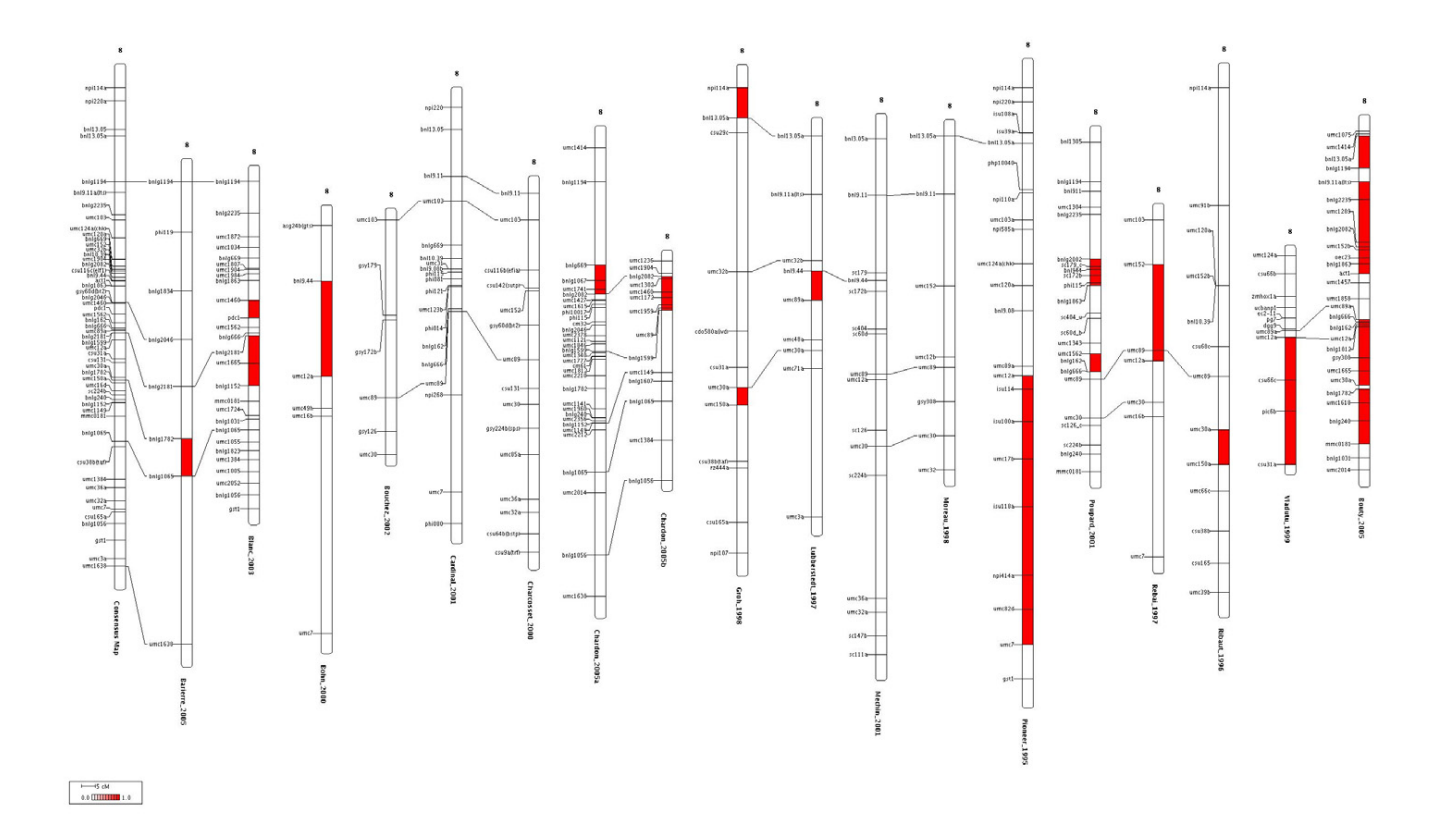
**Overview of the maize chromosomes 8 together with the consensus chromosome**. Overview of chromosome 8 for the 18 mapping experiments involved in the meta-analysis of flowering time in maize. The first chromosome at the left represents the consensus chromosome obtained by applying the WLS approach as described in the first section of the article (implemented into ConsMap). The filled marker intervals indicate that the standardized residual between the interval distance estimates of the original chromosome and the consensus one exceeded the double-sided 95% percentile of a normalized centered gaussian. This figure has been created by the program MMapView.

The consensus linkage group of chromosome 8 is depicted in Figure 3. The goodness-of-fit of the consensus chromosome is relatively bad: *λ *= 365.31 with *λ *~ χ872
 MathType@MTEF@5@5@+=feaafiart1ev1aaatCvAUfKttLearuWrP9MDH5MBPbIqV92AaeXatLxBI9gBaebbnrfifHhDYfgasaacH8akY=wiFfYdH8Gipec8Eeeu0xXdbba9frFj0=OqFfea0dXdd9vqai=hGuQ8kuc9pgc9s8qqaq=dirpe0xb9q8qiLsFr0=vr0=vr0dc8meaabaqaciaacaGaaeqabaqabeGadaaakeaacqaHhpWydaqhaaWcbaGaeGioaGJaeG4naCdabaGaeGOmaidaaaaa@317C@. It could be due to some heterogeneities in recombination rate among mapping experiments, located in the filled marker intervals of Figure 3. Note that variability of recombination rate in maize was first reported by [40] and, more recently, [41] demonstrated that exotic inbred lines exhibit higher recombination rate that U.S. inbreds origin along chromosome 1 (see also [42]). On the other hand, since no information about the marker configurations in each individual mapping experiment was available, the variances of the distance estimates have been computed by assuming no missing data and no ambiguities (dominance) in the original data sets. This is surely too optimistic and some data sets may have included missing data and/or dominant markers. Therefore the precision on the distance estimate may have been overestimated for some marker intervals.

#### Result of QTLProj

From the 18 QTL studies we projected 34 QTL on the consensus chromosome 8. Among these 34 QTL, 16 (47%) are related to SD, 10 (29%) to DPS and 8 (24%) to HT. The distribution of the r-square values clearly shows a L-shape: 75% of the QTL have r-square values lower than 12%. For 17 QTL a CI was reported (build from a 1-LOD support) from which we computed the standard deviations assuming that a 1-LOD support corresponds in fact to a 90% CI. For the other QTL we derived the standard deviations from the formula proposed by [29]. Then models from *K *= 1 to *K *= 10 QTL were considered and their parameters estimated by applying our EM-algorithm.

#### Result of QTLClust

**Figure 4:**
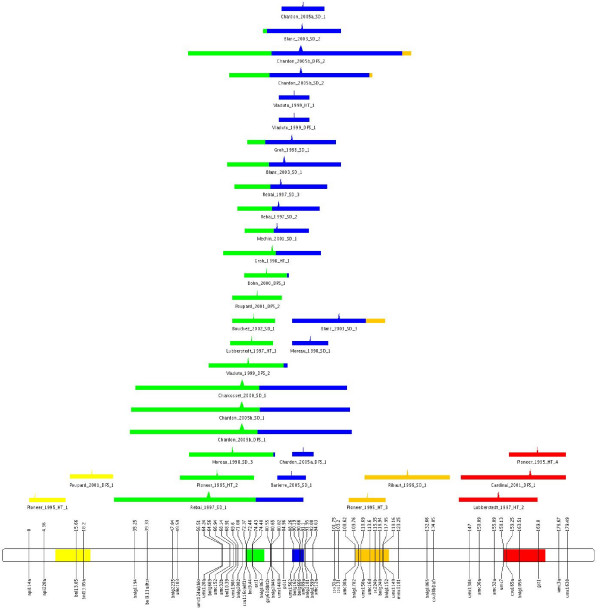
**Visualization of the QTL meta-analysis result on maize chromosome 8**. Result of the meta-analysis of the 34 QTL projected on the consensus chromosome 8. The CI of the meta-QTL positions are indicated on the chromosome by the filled colored areas. The observed QTL positions are depicted by their most probable position (triangle) and CI (segment). Membership probabilities of each initial QTL with respect to meta-QTL is visualized by the proportions of corresponding colored segments. This figure has been created by using MQTLView.

In Table 1 we give the values for the criteria AIC, AIC_*c*_, AIC3 and BIC for the different values of *K *explored. This clearly shows that the model with 5 QTL is the best one. Then, for the model with 5 QTL, the parameter estimates are listed in Table 2 and depicted in Figure 4. First, 3 QTL (1,4 and 5) have been detected in only 22% of the mapping experiments. At least two observed QTL are assigned to each of these 3 QTL without ambiguity.

Secondly, two closely linked QTL (2 and 3) contribute to 75% of the reported QTL. This is strongly consistent with the knowledge of this region where a major QTL, *vgt1*, is tightly linked to another QTL, *vgt2 *[43,44]. It is worth noting that the confidence interval of the QTL corresponding to *vgt1 *(around 3.8 cM) encompasses a marker interval of approximately 2 cM (at the left of the marker *umc89a*) in which this QTL has been finely mapped by [44] using NIL lines (result not included in our analysis). This congruency lends further credence to the meta-analysis approach.

## Discussion and conclusion

Nowadays more and more studies concerning QTL detection are available via public databases and the number of articles dealing with the comparison and/or integration of these results increases [12,45-47]. We believe that our meta-analysis procedure can contribute to facilitate the elaboration of such syntheses by providing a simple statistical framework to establish consensus models for both linkage maps and QTL locations.

First, the WLS strategy we proposed is a step forward to integrate several genetic marker maps. Contrary to iterative projection procedures, this approach provides a well-established statistical machinery (WLS) to assess the goodness-of-fit of the consensus model. It can also be used to test the homogeneity of the distance estimates among different mapping experiments. This can be usefull to investigate the possible variation of recombination rate among genotypes (as reported by [41]). As pointed out in the application, this method can suffer from the lack of knowledge about the effective precision on the marker interval distances in each individual mapping experiment due to possible missing data and/or the type of scoring of individual markers (codominance vs dominance). This could be improved by asking researchers to supply the variance estimates of the marker interval distances when they submit their results to a public database. These variance estimates could be used to improve the weight factors in the WLS model. Also, as sometimes robust framework maps are available in the literature or via public databases, the program ConsMap in **MetaQTL **offers the possibility to fix a genetic map as a reference (i.e for which the distances between ordered markers are assumed to be the "actual" distances). In this case, only the positions of the markers which are not reported on the reference have to be estimated.

Secondly, for the QTL meta-analysis itself, the Gaussian mixture model used to fit the distribution of the observed QTL locations on the chromosome provides a well-studied statistical inference technique. In this model-based clustering, each "true" QTL is mathematically represented by the Gaussian distribution of its detected positions, which leads to a probabilistic classification of the observed QTL. Contrary to [17] who developed a specific model choice criterion, our simulation results show that AIC gives relatively good performances in our QTL meta-analysis framework. This difference, with regard to the conclusions of [17], may be explained by their discrete formulation of the problem (recall that, instead of using a usual Gaussian mixture likelihood to evaluate the probability of the data, they assumed that the observations could be categorically assigned to the mixture components). Parameter estimates obtained by this approach were not really the maximum-likelihood estimates of the underlying mixture model. This may have added a bias in the evaluation of the AIC criterion, which could explain the bad performances of AIC in their simulations.

Thus, our mixture-modelling approach makes it possible to go beyond the limits encountered by [17]: the Akaike like criterion they proposed was limited to models from 1 to 4 QTL. As a consequence, [47] who used the method of [17], was obliged to break chromosomes on distinct segments to carry out the meta-analysis. This subjective division of the chromosome can now be avoided thanks to our method. Simulations have shown that the ratio between the number of observed QTL and the number of "true" QTL is one of the main limiting factor. The number of "true" QTL which can be assessed by the meta-analysis must be reasonable compared to the number of observed QTL (at least between 5 or 10 observed QTL per actual location). Note that this also depends on the distance between true QTL. But since there are more and more QTL locations reported for a given trait and since the real number of distinct QTL locations which can be detected with usual experimental designs is limited (only QTL with relatively large effects can be found), we assume that in many cases the ratio between observed and "true" QTL locations will steadily increase and should generally be reasonable. It is worth noting that, provided that the number of observed QTL is appropriate, the meta-analysis is able to separate "true" QTL locations even if they are closely linked (as illustrated in the application with *vgt1 *and *vgt2*, and the consistency of the *vgt1 *estimated position with fine mapping result of [44], result not included in the meta-analysis).

The ultimate step toward a more accurate identification of QTL relies on finding the underlying genes. Up to now, the majority of QTL isolated in plants have been cloned via positional cloning (see for instance [48]). However positional cloning of QTL is quite expensive both in terms of time and resources due to the necessity to screen recombinant individuals within large population (typically several hundreds) and to characterize these individuals with a very dense set of molecular markers. As an alternative and thanks to the advent of structural and functional genomics, QTL can also be resolved through association mapping of candidate genes. Candidate genes identification is based on a assumption that the polymorphism of the gene is associated with the variation of the trait of interest. Both function and mapping information have to be crossed to establish this assumption. The function of the gene may have been determined in the species of interest, based for instance on mutant analysis. More often, function is hypothesized based on sequence homology with genes the function of which has been established in model species, including possible positional cloning of QTL. Gene mapping information may have been obtained in the species of interest, but may have been also inferred from synteny based projections, as illustrated by [12] for rice to maize. Relevancy of the colocalization between QTL and candidate genes crucially depends on the confidence interval of the QTL positions. For this purpose the reduction of the confidence interval of the QTL is an important goal [2]. The ability of our method to reduce the QTL confidence interval by taking advantage of pooling QTL results could contribute in an increased resolution in selecting candidate genes. It is worth noting that candidate genes are generally mapped on a framework map used as reference for the species of interest (e.g. in maize [49]), while the QTL detections are carried out using specific populations (generally obtained by crossing parents contrasted for the trait(s) of interest).

Therefore, the selection of candidate genes which colocalize with QTL depends also on the process used to merge these different maps. Up to now, no statistical method had been proposed to combine candidate genes and QTL mapped in independent experiments, so we think that our WLS strategy should increase the precision of the integration of candidate gene mapping information.

Finally once candidate genes have been selected and their different haplotypes defined, association studies can be carried out. The identification of a statistically significant association between haplotype variation at a candidate gene and the target trait gives further credence on the role of this gene in the trait variation. Since the last 5 years more and more association studies have been reported in plants [50]. It would be interesting to integrate these new results into a global meta-analysis framework. Further developments are needed to combine onto a synthetic model the different scale of mapping: from linkage mapping (QTL) to fine mapping (association studies).

## Availability and requirements

• Project name: MetaQTL

• Project home page: 

• Operating system: Platform independent

• Programming language: Java

• Other requirements: Java 5.0

• License: GNU GPL

## Authors' contributions

B.G and A.C conceived the initial idea. J-B.V. developed and designed the project: he coded the package **MetaQTL**, developed the simulation tests, applied **MetaQTL **on the flowering time maize data set. All authors contributed to the interpretation of the results. Then J-B.V. and A.C. drafted the manuscript. All authors read and approved the final version of the manuscript.

## Supplementary Material

Additional file 1 **Theory and method for the computation of the variance of the recombination rate estimate**. This PDF file contains a short review of the theory and the methods to compute the variance of the recombination rate estimator for different kinds of pairwise marker configurations and different types of mapping experiments. Click here for file

Additional File 2 **Model choice by information based criterion**. This PDF file deals with a short review of the theory underlying some well-known information based criteria to select the number of components in a mixture model. Click here for file

Additional File 3 **Simulation study for the meta-analysis of genetic maps**. This PDF file describes two simulation scenarios which have been used to evaluate our weighted least squares strategy to build a consensus marker map. Click here for file

Additional File 4 **Supplementary Tables**. This PDF file contains the two supplementary tables. Click here for file

Additional File 5 **Documentation of MetaQTL**. The documentation of the Java package MetaQTL in PDF format. Click here for file

Additional File 6 **Summary of the QTL mapping experiments used in the application**. This PDF file gives details on the mapping experiments used in our application. Click here for file

Additional File 7 **MetaQTL Package : jar file and tutorial**. This Zip archive contains both the MetaQTL JAR file and the files of the tutorial. Click here for file
