# Supplementary material for: MetaQTL: a package of new computational methods for the meta-analysis of QTL mapping experiments
Source: BMC Bioinformatics. 2007 Feb 8;8:49. doi: 10.1186/1471-2105-8-49 (PMC1808479; doi:10.1186/1471-2105-8-49)
Supplement: Additional file 1 — Theory and method for the computation of the variance of the recombination rate estimate. This PDF file contains a short review of the theory and the methods to compute the variance of the recombination rate estimator for different kinds of pairwise marker configurations and different types of mapping experiments. [file 1471-2105-8-49-S1.pdf]

Theory and method for the computation of the variance of the recombination rate estimate

Let's assume that g classes of genotype are expected in the frequencies $\{p_j\}_{j=1,\ldots,g}$ which are function of r, the recombination fraction. [3] have discussed in details how to estimate r from the derivative of the log-likelihood,

$$\frac{\partial L}{\partial r} = \sum_{j=1}^{g} a_j \frac{\partial p_j}{\partial r}$$

where a_j is the observed number of genotypes for the jth class, $j = 1, \ldots, g$. He also defined the mean amount of information, i_r, supplied by a single individual as,

$$i_r = \sum_{j=1}^{g} \left[\frac{1}{p_j} \left(\frac{\partial p_j}{\partial r} \right)^2 \right]$$

from which the standard error of r, σ, can be derived: $\sigma = (N i_r)^{-1}$ where N is the number of individuals in the family.

Let's consider the results of crossing two parents $AABB$ and $aabb$. For backcross design $g = 4$ classes can be discriminated, namely $AABB$, $AABb$, $AaBB$, $AaBb$, and the individual information is given by

$$i_r = \frac{1}{r(1-r)}.$$

For selfed population usual marker technics makes it possible to distinguish nine genotypic classes (e.g RFLP markers showing codominant segregation) over the ten possible genotypic configurations: the $AaBb$ class generally includes the two double heterozygous genotypes AB/ab and Ab/aB unless the phase can be resolved. The expected frequencies $p_1, p_2, \ldots, p_9$ can be expressed in terms of the [1] zygotic proportions:

$$\begin{cases} C_t & = AABB + aabb = p_1 + p_2 \\ D_t & = AAbb + aaBB = p_3 + p_4 \\ 2E_t & = AABb + AaBB + Aabb + aaBb = p_5 + \ldots + p_8 \\ \frac{1}{2}(F_t + G_t) & = ABab = p_9 \end{cases}$$

where t is the number of selfing generations. The classes C_t, D_t, E_t, F_t and G_t are subject to the constraint $2C_t + 2D_t + 4E_t + F_t + G_t = 2$ so that $C_1 = D_1 = E_1 = G_1 = 0$ and $F_1 = 2$ [1]. It follows that the mean average

amount of information in a selfed population following t generations of self-fertlization is given by,

$$i_r = \frac{1}{C_t}\left(\frac{\partial C_t}{\partial r}\right)^2 + \frac{1}{D_t}\left(\frac{\partial D_t}{\partial r}\right)^2 + \frac{2}{E_t}\left(\frac{\partial E_t}{\partial r}\right)^2 + \frac{1}{2(F_t+G_t)}\left(\frac{\partial (F_t+G_t)}{\partial r}\right)^2$$

where the derivatives of the expected frequencies of each class with respect to r can be obtained by derivating the recurrence equations.

Self-fertilized recombinant inbred line population is a limit case of selfed population when $t \to \infty$. [1] have demonstrated that the fraction of crossover events observed, R, is related to the recombination frequency r per meiosis by the formula,

$$r = D(R) = \frac{R}{2(1-R)}$$

In term of R the mean amount of information is similar to the backcross case and leads to,

$$i_R = \frac{1}{R(1-R)}$$

It can be showed that i_r is directly related to i_R by the equation,

$$\begin{aligned} i_r &= \left(\frac{\partial D(R)}{\partial R}\right)^{-2} i_R \\ &= \frac{2}{r(1+2r)^2} \end{aligned}$$

More recently [2] and [4] have extended the Haldane and Waddington [1] equations to the case of intermated populations. It comes from these results that i_r of a single lineage in an population following t generations of random mating is given by,

$$i_r = \frac{(1-r)^{2t-2}[2(1-r)+t(1-2r)]^2[1+3(1-2r)^2(1-r)^{2t}]}{[1-(1-2r)^4(1-r)^{4t}]}$$

and that the relation between the fraction of crossover events observed in a self-fertilized intermated recombinant inbred population to the recombination frequency per meiosis is,

$$R = \frac{1}{2}\left(1 - \frac{1-2r}{1+2r}(1-r)^t\right).$$

Then $D(R)$ is the function which values are the solutions of the equation

$$2[1-D(R)]^{t+1} - [1-D(R)]^t + [2-4R][1-D(R)] + 3[2R-1] = 0$$

Although in this case there are none analytical formula of both $r = D(R)$ and $i_r = \left(\frac{\partial D(R)}{\partial R}\right)^{-2} i_R$, this quantities can be evaluated using standard numerical methods. Finally if a pair of markers is fully informative or if there is enough individuals with no missing information σ can be consistently estimated by applying the above strategy according to the family structure. Otherwise the number of classes to consider is the number of observed classes $\tilde{g} < g$ and σ can be computed using the same procedure by substituting g by $\tilde{g}$.

Bibliography

[1] J.B.S Haldane and C.H. Waddington. Inbreeding and Linkage. *Genetics*, 16:357–374, 1930.

[2] S C Liu, S P Kowalski, T H Lan, K A Feldmann, and A H Paterson. Genome-wide high-resolution mapping by recurrent intermating using Arabidopsis thaliana as a model. *Genetics*, 142(1):247–258, Jan 1996.

[3] K. Mather. Types of linkage data and their value. *Ann. Eugenics*, 7:251–264, 1936.

[4] Christopher R Winkler, Nicole M Jensen, Mark Cooper, Dean W Podlich, and Oscar S Smith. On the determination of recombination rates in intermated recombinant inbred populations. *Genetics*, 164(2):741–745, Jun 2003.
