# Supplementary material for: MetaQTL: a package of new computational methods for the meta-analysis of QTL mapping experiments
Source: BMC Bioinformatics. 2007 Feb 8;8:49. doi: 10.1186/1471-2105-8-49 (PMC1808479; doi:10.1186/1471-2105-8-49)
Supplement: Additional File 2 — Model choice by information based criterion. This PDF file deals with a short review of the theory underlying some well-known information based criteria to select the number of components in a mixture model. [file 1471-2105-8-49-S2.pdf]

Model choice by information based criterion

Assuming that model is true, regularity conditions of the loglikelihood and asymptotic normality of the MLE, [2] proved that B can be asymptotically approximated by the number of free parameters in the model. This leads to the well-established expression,

$$\mathrm{AIC} = -2L(\hat{X}, \Sigma; \tilde{\Theta}^{[K]}) + 2\nu$$

as discussed where $\nu = 2K - 1$. When ν is large relative to the sample size q there is a small-sample version of AIC called AIC_c,

$$\mathrm{AIC}_c = -2L(\hat{X}, \Sigma; \tilde{\Theta}^{[K]}) + 2\nu + \frac{2\nu(\nu+1)}{q-\nu-1}$$

which should be used unless $q/\nu >$ about 40 for the model with the largest value of ν (see [11]). These easily computable information criteria are also an extension of Fisher's loglikelihood theory [3]. It is worth mentioning that without assuming that the model is true [12] derived an asymptotically unbiased estimator of expected log-likelihood. His method, which requires quite large sample sizes to reliably estimate the bias adjustment term (details in [7]), leads in many cases to a correction approximatively equal to ν giving further credence to use AIC and AIC_c in practice.

But since AIC and AIC_c rely on the usual asymptotic theory of the MLE and that the regularity conditions do not hold when comparing two constrated mixture models they are not a correct way of comparing models in the case of mixture (see [13], [1]). However due to its simplicity and its compelling concept AIC have been widely used in mixture model applications and seems to yield relatively good performances in simulation studies (see for instance [4]).

By means of a Monte-Carlo approach Wolfe [15] obtained an approximation of the null distribution of the loglikelihood ratio test (LRT) when testing two contrasted hypotheses on the number of components in a Gaussian mixture. Bozdogan [5] proposed to use this approximation leading to a modified AIC criterion, namely AIC3 defined by

$$\mathrm{AIC3} = -2L(\hat{X}, \Sigma; \tilde{\Theta}^{[K]}) + 3\nu$$

More recently Bozdogan [6] proposed an informational complexity criterion, called ICOMP, for choosing parsimonious models which requires to compute the Fisher information matrix of the model which can make its computation tedious (Cutler and Windham [9] suggested to approximate the Fisher information matrix with its empirical mean to derive ICOMP). It is worth

mentioning that Windham and Cutler [14] have also introduced another criterion, called MIR, which is based on the smallest eigenvalue of the ratio of Fisher information matrices (MIR can be computed from the EM convergence rate).

Another widely used criterion in both frequentist and bayesian mixture context was originally proposed by Schwarz [10], the bayesian information criterion defined as

$$\mathrm{BIC} = -2L(\hat{X}, \Sigma; \Theta) + \nu \log(q)$$

which is based on an approximation of the Bayes factor. Note that BIC can also be derived as a non-Bayesian result and that like AIC the BIC approximation is only valid when standard regularity conditions regarding the loglikelihood are verified. Burnham and Anderson [8] give more detail on the deep foundations of both BIC and AIC.

Finally, whatever the information criterion used to select the best model the individual criterion values are not generally interpretable. It is imperative to rescale its values. For example, the Akaike information criterion, AIC, can be rescaled as follows:

$$\Delta_K = AIC_K - AIC_{K^*}$$

where K^* is the value of K which gives the minimal value of AIC for the $K_{\max}$ different AIC_K values. Δ_K is easy to interpret as the information loss experienced if we are using a model with K components rather than the best model with K^* components for inference. Hence the Δ_K's allow a quick stength-of-evidence comparison and ranking candidate models. In particular one can compute the useful "weights of evidence" w_K given by,

$$w_K = \frac{\exp(-\Delta_K/2)}{\sum_{j=1}^{K_{\max}} \exp(-\Delta_j/2)}$$

which can be interpreted as the probabilty that model K is in fact the best model for the data.

Bibliography

[1] M. Aitkin and D.B. Rubin. Estimation and Hypothesis Testing in Finite Mixture Models. *Journal of the Royal Statistical Society*, 47:67–75, 1985.

[2] H. Akaike. Information theory and an extension of the maximum likelihood principle. *2nd Inter. Symp. on Information Theory*, pages 267–281, 1973.

[3] H. Akaike. *Breakthroughs in Statistics*, volume 1, chapter Information Theory and an Extension of the Maximum Likelihood Principle, pages 610–624. Springer-Verlag, London, 1992.

[4] C. Biernacki and G. Govaert. Choosing Models in Model-based Clustering and Discriminant Analysis. Technical Report 3509, INRIA, France, 1998.

[5] H. Bozdogan. Model Selection and Akaike Information Criteria (AIC) : The general theory and its analytic extensions. *Psychometrika*, 52:345–370, 1987.

[6] H. Bozdogan. On the Information-Based Measure of Covariance Complexity and its Application to the Evaluation of Multivariate Linear Models. *Communication in Statistics, Theory and Methods*, 19:221–278, 1990.

[7] K. P. Burnham and D. R. Anderson. *Model Selection and Multimodel Inference: A Pratical Information-Theoretical Approach*, volume 33. Springer-Verlag, New-York, 2 edition, 2002.

[8] K. P. Burnham and D. R. Anderson. Multimodel Inference, Understanding AIC and BIC in Model Selection. *Sociological Methods & Research*, 33(2):261–304, November 2004.

[9] A. Cutler and M. P. Windham. Information-Based Validity Functionals for Mixture Analysis. In Bozdogan H., editor, *Proceedings of the first*

US-Japan Conference on the Frontiers of Statistical Modeling, pages 149–170, Amsterdam, 1993. Kluwer.

[10] Schwarz. Estimating the Dimension of a Model. *Annals of Statistics*, 6:461–464, 1978.

[11] N. Sugiura. Further Analysis of the Data by Akaike's Information Criterion and the Finite Corrections. *Communications in Statistics, Theory and Methods*, A(7):13–26, 1978.

[12] K. Takeuchi. Distribution of informational statistics and a criterion model fitting. *Math. Sci.*, 153:12–18, 1976.

[13] D.M. Titterington, A.F. Smith, and U.E. Markov. *Statistical Analysis of Finite Mixture Distributions.* John Wiley and Sons, New York, 1985.

[14] M.P. Windham and A. Cutler. Information Ratios for Validating Mixture Analyses. *J. Am. Stat. Ass.*, 87:1188–1192, 1992.

[15] J.H. Wolfe. A Monte Carlo study of sampling distribution of the likelihood ration for mixtures of multinormal distributions. *Technical Bulletin STB*, 72-2, 1971.
