# Supplementary material for: MetaQTL: a package of new computational methods for the meta-analysis of QTL mapping experiments
Source: BMC Bioinformatics. 2007 Feb 8;8:49. doi: 10.1186/1471-2105-8-49 (PMC1808479; doi:10.1186/1471-2105-8-49)
Supplement: Additional File 3 — Simulation study for the meta-analysis of genetic maps. This PDF file describes two simulation scenarios which have been used to evaluate our weighted least squares strategy to build a consensus marker map. [file 1471-2105-8-49-S3.pdf]

Simulation study for the meta-analysis of genetic map

Scenario 1

As Γ is based on a simple Taylor expansion at the first order, the meta-analysis result could suffer from a lack of precision of the variance estimates. Moreover the variance estimates are generally computed using the maximum-likelihood estimate of the recombination rate, which is also an approximation. A simple scenario was explored in order to investigate the impact of these approximations on the consensus linkage group construction. We considered a single chromosome on which 21 markers were spread by randomly drawing 20 marker interval distances in a Gaussian distribution with mean equals to 10 cM and standard deviation of 2 cM. This Gaussian distribution of marker interval distances allows to create a variety of marker configurations with intervals of reasonable length. For a given pedigree, n population data sets were simulated for each marker configuration (all the markers were assumed to be fully informative). For all the mapping experiments we fixed the number of individuals to 200. For each data set the recombination fractions were estimated using a usual maximum likelihood procedure. The order of markers was assumed to be known and the consensus linkage group was built by our WLS strategy. Finally, 50 marker configurations were drawn for a given pedigree and 100 replicates were done for each marker configuration.

For each individual mapping experiment we define the Interval Mean Square Error (IMSE) as $\text{IMSE}(i) = \frac{1}{J}\sum_{j=1}^{J} E(\hat{r}_{i,j} - r_j)^2$ where J is the number of intervals (here $J = 20$), $\hat{r}_{i,j}$ the estimation of the recombination rate in the jth marker interval in the ith mapping experiment and r_j is the true recombination rate. This indicator measures the quality of an estimated marker map relatively to the "true" marker map. It comes immediately that the expected IMSE(i) is $\frac{1}{J}\sum_{j=1}^{J}\eta_{i,j}^2$ where $\eta_{i,j}^2$ is the variance of the recombination rate estimate in the jth marker interval in the ith mapping experiment. Using the distance estimates obtained by the WLS approach, it is also possible to compute this indicator for the consensus linkage group, denoted IMSE(c). Therefore in order to evaluate the quality of the consensus linkage group we computed the quantity $\overline{\text{IMSE}} = \frac{1}{n}\sum_{i=1}^{n} \text{IMSE}(i)/\text{IMSE}(c)$. If the n mapping experiments share the same family structure and if the approximation made on the variance estimates are not too crude, $\overline{\text{IMSE}}$ should be equal to n.

Scenario 2

Secondly, mapping experiments generally do not have all their markers in common. In this case, the proportion, p, of common markers between the mapping experiments can be a limiting factor to carry out the meta-analysis. In order to study the impact of p on the quality of the consensus linkage group, we investigated a 200 cM long chromosome covered by 2001 markers equally spaced by 0.1 cM. Each mapping experiment consists in a scattering view of the original chromosome with a limited number of markers randomly picked but subject to a constraint on marker interval distances in order to avoid both too tiny or too large intervals. This constraint was set so that all the mapping experiments have marker intervals with distances laying between 5 and 30 cM. Finally, for a given number n of mapping experiments, p was defined as the ratio between the average number of common markers over all the pairs of individual maps and the total number of markers M. The number of markers per mapping experiments was set to 20. In our simulation study we focused on 4 common marker proportion configurations: $p = 0.15, 0.25, 0.50$ and 0.75 which correspond to respectively $3, 5, 10$ and 15 common markers between pairs of mapping experiments. For a given type of population, a given number of populations n and a given value of p, 25 marker configurations were generated. Each replicate consisted in 100 simulated data sets and for each data set the linkage group of the n mapping experiments were constructed as for the first simulation procedure.

For this second simulation scenario there are two ways to evaluate the quality of the consensus linkage group. As previously proposed, we first computed the quantity $\overline{\text{IMSE}}$. Note that in this case IMSE(i) and IMSE(c) are not computed on the same marker intervals and we cannot presume the value of the ratio of these two quantities. Nevertheless it is a practical way to evaluate the mean square error of the consensus linkage relatively to the ones of the individual mapping experiments. We can also look at the ability of the consensus model to predict the marker interval recombination fractions in the individual mapping experiments. This can be done by substituting in each IMSE(i) the recombination rate estimates computed from the experiment data set by those deduced from the consensus linkage group. This leads to the Interval Mean Square Error of Prediction (IMSEP). Thus the ability of the consensus model to predict the recombination rate estimates in each mapping experiment can be evaluated by the indicator $\overline{\text{IMSEP}} = \frac{1}{n} \sum_{i=1}^{n} \text{IMSE}(i)/\text{IMSEP}(i)$.

Results

The results of simulations for the first scenario are depicted in Figure 1 for three different population types. As expected, the observed ratio increases with n, despite slightly lower than the value expected if the true variances for distance estimation would be used. Whatever the kind of population, this indicates that our approximation is not too crude and that further theoretical developments would only have a minor effect.

In Table 1 we reported the results of the second simulation scenario. This shows that the proportion of common markers can have a strong impact on the estimations of the consensus marker interval distances. For example when 2 mapping experiments have less than 75% of common markers, both $\overline{\text{IMSE}}$ and $\overline{\text{IMSEP}}$ indicate a substantial loss in quality on the recombination rate estimates of the consensus linkage group. This is partially removed when n increases. However the results indicates that when n increases, although the WLS approach leads generally to a consensus model with a good quality of prediction (measured by $\overline{\text{IMSEP}}$) whatever the proportion p of common markers, the intrinsic quality of the result (measured by $\overline{\text{IMSE}}$) strongly depends on this proportion (for $n = 10$ and $p = 0.15$ and 0.25, the consensus linkage group have in average a lower IMSE than the individual mapping experiments). This can be explained by the fact that, for a given proportion of common markers p, the number of markers to position on the consensus linkage groups increases with n. In other word the gain due to the amount of information brought by the combination of common markers over the experiments is balanced by the markers which are only observed in a single mapping experiment (e.g. in our simulation the average number of distinct markers for $n = 2, 5, 10$ and $p = 0.75$ was $M = 30, 32, 36$).

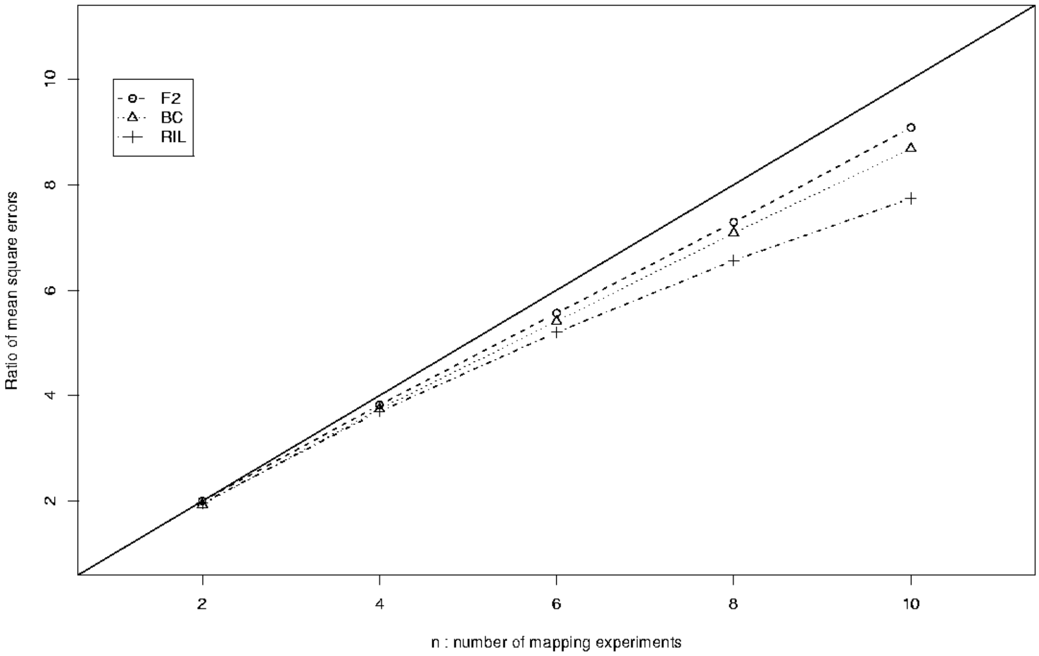

Figure 1: Average values of $\overline{IMSE}$ over 50 marker configurations (scenario 1) for 3 kinds of pedigree: backcross (BC), F2 and recombinant inbred lines via selfing (RIL). The solid line represents the expected values of $\overline{IMSE}$.

Pedigree		n											
		2				5				10			
	p =	0.15	0.25	0.50	0.75	0.15	0.25	0.50	0.75	0.15	0.25	0.50	0.75
BC	$\overline{\text{IMSE}}$	0.28	0.44	0.82	**1.24**	0.33	0.52	**1.01**	**1.62**	0.43	0.64	**1.02**	**1.65**
	$\overline{\text{IMSEP}}$	0.74	0.78	0.95	**1.06**	0.97	**1.17**	**1.62**	**2.08**	**1.32**	**1.67**	**2.45**	**3.30**
F2	$\overline{\text{IMSE}}$	0.24	0.54	0.87	**1.39**	0.33	0.58	**1.22**	**1.97**	0.46	0.76	**1.40**	**3.01**
	$\overline{\text{IMSEP}}$	0.71	0.76	0.90	**1.02**	0.94	**1.16**	**1.60**	**1.97**	**1.30**	**1.67**	**2.35**	**2.80**

Table 1: Average values of $\overline{\text{IMSE}}$ and $\overline{\text{IMSEP}}$ over 25 marker configurations (scenario 2) for a given proportion p of common markers between n mapping experiments, for backcross (BC) and F2 designs. The values are indicated in bold when the meta-analysis leads to a consensus linkage groups with a better quality in terms of recomination rate estimates than the individual mapping experiments.
