# Supplementary material for: MetaQTL: a package of new computational methods for the meta-analysis of QTL mapping experiments
Source: BMC Bioinformatics. 2007 Feb 8;8:49. doi: 10.1186/1471-2105-8-49 (PMC1808479; doi:10.1186/1471-2105-8-49)
Supplement: Additional File 4 — Supplementary Tables. This PDF file contains the two supplementary tables. [file 1471-2105-8-49-S4.pdf]

K	q	δ							
		1		2		3		4	
		q90	q95	q90	q95	q90	q95	q90	q95
2	20	**0.75 (100%)**	**0.84 (100%)**	**0.52 (100%)**	**0.66 (98%)**	**0.39 (100%)**	**0.46 (100%)**	**0.29 (100%)**	**0.30 (100%)**
	50	**0.62 (100%)**	**0.74 (100%)**	**0.38 (100%)**	**0.55 (100%)**	**0.26 (100%)**	**0.33 (100%)**	**0.18 (100%)**	**0.19 (100%)**
3	20	0.94 (72%)	1.02 (38%)	**0.68 (100%)**	**0.84 (92%)**	**0.50 (100%)**	**0.59 (100%)**	**0.36 (100%)**	**0.38 (100%)**
	50	0.81 (100%)	0.91 (94%)	**0.53 (100%)**	**0.71 (100%)**	**0.34 (100%)**	**0.45 (100%)**	**0.23 (100%)**	**0.26 (100%)**
4	20	1.06 (18%)	1.10 (8%)	0.85 (86%)	1.01 (50%)	**0.63 (94%)**	**0.76 (90%)**	**0.42 (100%)**	**0.46 (100%)**
	50	0.92 (98%)	0.99 (56%)	**0.63 (100%)**	**0.82 (96%)**	**0.40 (100%)**	**0.51 (98%)**	**0.27 (100%)**	**0.33 (100%)**
5	20	1.13 (6%)	1.15 (2%)	1.03 (36%)	1.18 (6%)	0.74 (92%)	0.94 (70%)	**0.48 (100%)**	**0.57 (96%)**
	50	1.00 (40%)	1.05 (8%)	0.72 (98%)	0.92 (84%)	0.54 (96%)	0.73 (84%)	**0.33 (100%)**	**0.41 (94%)**

Table 1: Mean ratio of the length of the confidence interval at 90% (q90) and 95% (q95) between strategy 2 (meta-analysis) and 1 (naive). Recall that K is the number of “true” QTL, q the number of observed QTL, δ the Mahalanobis distance between the “true” QTL. The values between brackets indicate the percentage of times the meta-analysis approach led to a lower value of the quantile and the cells are in bold when the meta-analysis improved the precision on the QTL location in at least 90% of times.

Module	Program	Description
Data Base	MetaDB	Checks and summarizes all the input data into a set of XML files
Meta-analysis of genetic map	InfoMap	Displays information of the common marker configurations bewteen input marker maps
Meta-analysis of genetic map	ConsMap	Performs the WLS approach to build the consensus marker map
Meta-analysis of QTL	QTLProj	Projects QTL from a set of mapping experiments on a given marker map
Meta-analysis of QTL	QTLClust	Performs the Gaussian mixture algorithm
Meta-analysis of QTL	QTLClustInfo	Summarizes the resulf of QTLClust for a given meta-QTL model
Meta-analysis of QTL	QTLTree	Performs hierarchical agglomerative clustering on the QTL positions
Visualization	MapView	Creates an image of a chromosome with various displaying options
Visualization	MMapView	Creates an image of multiple chromosomes with various displaying options
Visualization	MQTLView	Creates an image of the QTL meta-analysis result with various displaying options
Utilities	Xml2A	Converts XML files into plain text ASCII files
Utilities	A2Xml	Converts plain text ASCII files into XML files

Table 2: Description of the programs implemented in the **MetaQTL** package. All the programs are command line programs and can be combined in various ways to carry out a complete integration of multiple QTL mapping experiments.
