# Supplementary material for: MetaQTL: a package of new computational methods for the meta-analysis of QTL mapping experiments
Source: BMC Bioinformatics. 2007 Feb 8;8:49. doi: 10.1186/1471-2105-8-49 (PMC1808479; doi:10.1186/1471-2105-8-49)
Supplement: Additional File 5 — Documentation of MetaQTL. The documentation of the Java package MetaQTL in PDF format. [file 1471-2105-8-49-S5.pdf]

MetaQTL

Reference Manual
Edition 1.0, for MetaQTL Version 1.0
20 August 2005

J.-B. Veyrieras (`veyrieras@moulon.inra.fr`)
B. Goffinet
A. Charcosset

Copyright © 2005 Jean-Baptiste Veyrieras, INRA.

Permission is granted to copy, distribute and/or modify this document under the terms of the GNU Free Documentation License, Version 1.2 or any later version published by the Free Software Foundation; with the Invariant Sections being "GNU General Public License" and "Free Software Needs Free Documentation", the Front-Cover text being “A GNU Manual”, and with the Back-Cover Text being (a) (see below). A copy of the license is included in the section entitled “GNU Free Documentation License”.

(a) The Back-Cover Text is: “You have freedom to copy and modify this GNU Manual, like GNU software. Copies published by the Free Software Foundation raise funds for GNU development.”

Short Contents

1 Introduction 1
2 Data Base 4
3 Meta Analysis 8
4 Visualization 18
5 Utilities 31
6 Tutorial 36
A Copying This Manual 54

Table of Contents

1 Introduction ... 1
1.1 General Overview ... 1
1.1.1 What is MetaQTL ... 1
1.1.2 Definition of the Problem ... 1
1.2 Citing MetaQTL ... 2
1.3 How to Get and Install MetaQTL ... 2
1.4 Getting Help ... 2
1.5 General Usage of the Programs ... 2
1.6 References ... 2

2 Data Base ... 4
2.1 Overview ... 4
2.1.1 Experiment Table ... 4
2.1.2 Trait Ontology Table ... 5
2.1.3 GLMdb ... 5
2.1.4 QTLdb ... 6
2.2 MetaDB ... 6
2.2.1 Command Line Options ... 6

3 Meta Analysis ... 8
3.1 Consensus map ... 8
3.1.1 InfoMap ... 8
3.1.1.1 Method ... 8
3.1.1.2 Command Line Options ... 8
3.1.1.3 Output ... 9
3.1.2 ConsMap ... 10
3.1.2.1 Method ... 10
3.1.2.2 Command Line Options ... 10
3.1.3 Output ... 11
3.2 QTL Projection ... 11
3.2.1 Method ... 11
3.2.2 QTLProj ... 12
3.2.2.1 Command Line Options ... 12
3.2.2.2 Output files ... 12
3.3 QTL Clustering ... 13
3.3.1 ClustQTL ... 13
3.3.1.1 Method ... 13
3.3.1.2 Command Line Options ... 13
3.3.1.3 Output ... 13
3.3.2 QTLTree ... 15
3.3.2.1 Method ... 15
3.3.2.2 Command Line Options ... 16
3.3.2.3 Output ... 16

4 Visualization 18
4.1 MapView 18
4.1.1 Command Line Options 18
4.1.2 Drawing Parameters 18
4.2 MMapView 21
4.2.1 Command Line Options 21
4.2.2 Drawing parameters 22
4.3 MQTLView 25
4.3.1 Command Line Options 25

5 Utilities 31
5.1 A2Xml 31
5.1.1 Command Line Options 31
5.2 Xml2A 33
5.2.1 Command Line Options 33
5.3 QTLClustInfo 33
5.3.1 Command Line Options 33
5.4 QTLModel 34
5.4.1 Command Line Options 34
5.5 References 35

6 Tutorial 36
6.1 Tutorial Introduction 36
6.2 Step 1 - Creating the XML Database 36
6.3 Step 2 - Building the consensus map 36
6.3.1 Checking chromosome connection and marker order consistency 36
6.3.2 Building the consensus map 41
6.4 Step 3 - Projection of the QTL 45
6.5 Step 4 - QTL Clustering 47
6.5.1 QTL Clustering 47
6.5.2 QTL Tree 50
6.6 Step 4 - Post processing 52
6.6.1 Extracting the meta-QTL 52
6.6.2 Projection of the meta-QTL 52

Appendix A Copying This Manual 54
ADDENDUM: How to use this License for your documents 60

1 Introduction

1.1 General Overview

1.1.1 What is MetaQTL

MetaQTL is a suite of programs designed to carry out meta-analysis of QTL mapping experiments. A QTL mapping experiment consists in a genetic linkage map and a set of quantitative trait loci (QTL) which have been detected and positioned onto the genetic linkage map. This package is composed by several programs written in pure Java. These programs can perform various tasks, including reformatting, analyzing data and visualizing the results of the analyses. Presently the programs can handle data from backcross, intercrosses and recombinant inbreds, as well as a few other experimental designs. This project is ongoing and suggestions are welcome for further improvements and enhancements.

1.1.2 Definition of the Problem

Since the last decade, the advent of molecular markers have accelerated the pace of discovering the loci which are implied in quantitative trait variation. Quantitative Trait Loci (QTL) mapping usually begins with the collection of genotypic (based on molecular markers) and phenotypic data from a segregating population. First, from the genoptypic data the markers are both ordered and positioned on a genetic map using standard linkage mapping approaches. Secondly, refinement of analytical methods have enabled to detect one ore several QTL on each chromosome (see for instance Lander and Botstein 1989, Zeng 1994). Nevertheless due to the limiting number of individuals and generations in usual experiment this approach generally leads to QTL locations with a confidence interval (CI) around 10 cM (Kearsey 1998) which in plant generally corresponds to a thousand of genes or more.

Due to its relative simplicity and its compelling concept QTL mapping has been widely used and more and more QTL detection results are now available in public databases (e.g in maize at `http://www.maizegdb.org`). One of the main purpose of these databases was to facilitate the comparison of different QTL detection results by providing both standard description of these results and ontologies (see for instance the trait ontology at `http://www.gramene.org/plant\_ontology/`). Relevance of comparative analysis of QTL studies have been illustrated by several authors (Khavkin 1997 and 1998, Lin 1995). However these studies often relied on simple descriptive statistics.

QTL congruency study was partially improved thanks to Goffinet and Gerber (2000) who proposed a meta-analysis based approach in order to integrate QTL results from several experiments. Their method makes it possible to evaluate how many "actual" QTL locations underly the distribution of the observed QTL on the genome. This approach has been implemented in BioMercator by Arcade et al. (2004). This software allows user to merge both markers and QTL onto a consensus map by means of an iterative projection procedure. Then the algorithm devised by Goffinet and Gerber (2000) can be applied to evaluate the likelihood of clustering the observed QTL in 1,2,3 or 4 groups. Afterward, the optimal number of clusters is selected by using a Akaike like criterion. Alghough original this approach suffers from the absence of indicator to assess the consensus map quality and from the limiting number of QTL clusters which can be explored.

Based on recent methodological developments, MetaQTL implements a series of Java programs in order to carry out whole-genome QTL meta-analysis. All the programs in MetaQTL are command line programs. Each program does a small job and the user can easily combine the programs as a group to do a complete analysis.

1.2 Citing MetaQTL

In publications you should cite this manual.

- Veyrieras J-B., Goffinet B. and Charcosset A., 2005. MetaQTL, Version 1.0. INRA, France.

1.3 How to Get and Install MetaQTL

MetaQTL is avalaible at `http://bioinformatics.org/mqtl`. As **MetaQTL** is entirely written in Java (1.5) it should be run on most of operating systems provided that a compatible Java Virtual Machine (1.5) has been previously installed on your machine. Otherwise go to `http://java.sun.com/downloads/index.html`: then download and install the last version of the Java 2 Platform. To run the programs of **MetaQTL** you must have updated the Java *CLASSPATH* by adding the path to the jar archive '`metaqtl.jar`' included into the **MetaQTL** distribution. Under an UNIX like operating system this can be done as follows

```
bash$ export CLASSPATH=${CLASSPATH}:/path/to/metaqtl/directory/metaqtl.jar
```

Under Windows 9x/NT/2000/XP you can look at `http://support.microsoft.com/` to find how to update or create the environment variable *CLASSPATH*. Generally, to view or change environment variables you have to apply the following steps:

- Right-click My Computer, and then click Properties.
- Click the Advanced tab.
- Click Environment variables.
- Click one the following options, for either a user or a system variable:
  - Click New to add a new variable name and value.
  - Click an existing variable, and then click Edit to change its name or value (here the value will be the entire file path from the root of the disk to the jar archive of MetaQTL).

In the next sections, the examples are given assuming the *CLASSPATH* have been correctly set.

1.4 Getting Help

For any questions or troubles about the use of **MetaQTL**, contact J.B. Veyrieras by email at `veyrieras@moulon.inra.fr`.

1.5 General Usage of the Programs

The programs in the **MetaQTL** suite all have the same look and feel and are heavily influenced by UNIX programs. They can only be used as command line programs. The options `-v, --verbose` and `-h, --help` are common to all the programs. The first one activates the verbosity mode and the second one can be used to display a brief help on program usage.

1.6 References

- A. Arcade, A. Labourdette, M. Falque, B. Mangin, F. Chardon *et al.*, 2004, *BioMercator: integrating genetic maps and QTL towards discovery of candidate genes*, Bioinformatics, 20:2324-2326.
- A.P. Dempster, N.M. Laird, D.B. Rubin, 1977, *Maximum Likelihood from incomplete data via the EM algorithm (with discussion)*, J. Roy Stat. Soc., B 39:1-38.
- B. Goffinet and S. Gerber., 2000, *Quantitative trait loci: a meta-analysis*, Genetics, 155(1):463-473.
- M.J. Kearsey and A.G.L. Farquhar., 1998, *QTL analysis in plants; where are we now?*, Heredity, 80:137-142.

- E. Khavkin and E. H. Coe., 1997, *Mapped genomic locations for developmental functions and QTLs reflect concerned groups in maize (Zea mays L.)*, Theor. Appl. Genet., 95:343-352.
- E. Khavkin and E. H. Coe., 1998, *The major quantitative trait loci for plant stature, development and yield are general manifestations of developmental gene clusters*, Maize Newslett., 72:60-66.
- E. S.Lander and D. Botstein, 1989, *Mapping Mendelien factors underlying quantitative traits using RFLP linkage maps*, Genetics, 121:185-199.
- Y. R. Lin, K. F. Schertz, A. H. Paterson, 1995, *Comparative analysis of QTLS affecting plant height and maturity across the poaceae, in reference to an interspecific sorghum population*, Genetics, 141:391-411.
- T. Sasaki, T. Matsumoto, K. Yamamoto, K. Sakata, 2002, *et al.*, *The genome sequence and structure of rice chromosome 1*, Nature, 420(6913):312-316.
- J-B. Veyrieras, B. Goffinet, A. Charcosset, 2005, *Meta-analysis of QTL Mapping Experiments*, in prep., 2005.
- J. H. Ward., 1963, *Hierarchical Grouping to Optimize an Objective Function*, J. Am. Stat. Assoc., 58:236-244.
- Z-B. Zeng, 1994, *Precision mapping of quantitative trait loci*, Genetics, 136(4):1457-1468.

2 Data Base

2.1 Overview

The first phase in using **MetaQTL** is to create the database to store the QTL mapping experiments. The database here relies on a simple multiple file framework. All the tables of the database are plain text file where the first line contains the labels of the columns and the following lines represents the records. For both the header and the records the fields are separated by a TABULATION. The global database (METAdb) consists of 3 tables, the experiment table, the trait ontology table and the marker dictionary table, plus 2 sub databases, the genetic linkage map database (GLMdb) and the QTL database (QTLdb). In the next sections we provide a full description of the tables of the database. We have adopted the following convention to specify each field of the tables: the fields in bold are required, i.e they must be specified in the header of the file while the other can be ignored. For each field we have indicated the type of the value between brackets (e.g STRING, INTEGER, BOOLEAN,...).

2.1.1 Experiment Table

The experiment table summarizes the parameters of each mapping experiment. This table contains the following fields:

- **map** (STRING) : the name of the mapping experiment which must be unique.
- **mapping.function** (STRING) : the name of the mapping function used to build the genetic map for this mapping experiment. Presently the possible values are:
  - haldane
  - kosambi
- **mapping.unit** (STRING) : the unit of the genetic distances of the genetic linkage map. Possible values are M for Morgan or cM for centiMorgan.
- mapping expansion (BOOLEAN) : 1 if the genetic linkage map of this mapping experiment must be rescaled.
- mapping.cross : the cross design used to build the map (may differ from the cross.type).
- **cross.type** (STRING) : the cross design used to build the genetic linkage map. **MetaQTL** is able to deal with various types of experimental designs. The design is encoded by a string of characters. For example BC stands for backcross, SFi stands for selfed intercross lines and the integer i indicates the generation (if i=2 then we have a classical F2). Experimental designs presently supported are listed in the table below (the cross type naming and labelling convention comes from QTLCartographer).

**Cross type**	**Code**	**Example**
Backcross	BC	BC
Selfed generation i intercross	SFi	SF2
Randomly mated generation i intercross	RFi	RF3
Recombined Inbred via selfing	RI0	RI0
Recombined Inbred via sib mating	RI1	RI1
Intermated (i generations) Recombined Inbred via selfing	IRI0i	IRI04
Intermated (i generations) Recombined Inbred via sib mating	IRI1i	IRI13

- **cross.name** (STRING) : the name of the cross.
- **cross.size** (INTEGER): the number of individuals used to build the genetic linkage map.

For example, inserting 5 mapping experiments leads to the following file

```
map  mapping.function  mapping.unit  mapping.expansion  cross.type  cross.name  cross.size
exp1   haldane   cM   0   SF2   Mo17xB73   200
exp2   haldane   cM   1   RF4   F2xF252    300
exp3   kosambi   M    0   BC    H99xF2     150
exp4   haldane   cM   1   IRI04 F2xF252    400
exp5   haldane   cM   0   SF4   Mo17xF2    150
```

2.1.2 Trait Ontology Table

MetaQTL can also use a trait ontology in order to group the QTL in trait categories. The ontology representation handled by **MetaQTL** is based on a simple hierarchy (such as a taxonomy) where each child has a unique parent. The table which stores the trait ontology is defined by the following fields:

- **term_id**(INTEGER): the unique identifier of the term.
- **term_name**(STRING): the name of the term, i.e the trait name.
- **parent_id**(INTEGER): the identifier of the unique parent of this term (required except for the root of the ontology).
- synonyms(STRING): the synonyms of the trait name separated by a coma (then the synonyms must not contain any coma).
- definition(STRING): the definition of the trait.

For example:

```
term_id   term_name   parent_id   synonyms   definition
1       my_ontology
2       flowering_time          1
3       days_to_pollon_shed     2
4       plant_height            2
5       leaf_number             2
6       silking_date            2
```

REMARK: The trait ontology must always be rooted (in the previous example the root was `my_ontology`). Generally, The root is not itself a trait but the name of the ontology.

2.1.3 GLMdb

The genetic linkage map database is simply organized as a set of plain text files each corresponding to a single genetic linkage map. The link between the experiment table and the genetic map files is done by using the same stem file name than the name of the mapping experiment declared in the column **map** of the experiment table. For example the genetic linkage map related to the mapping experiments '`exp1`' will be stored in a file named '`exp1.txt`'. Here a genetic linkage map is represented by a table with the following fields:

- **group** (STRING) : the linkage group name.
- **marker** (STRING) : the name of the marker.
- **position** (NUMERIC) : the position of the marker on the linkage group the unit of which is declared in the experiment table.

For example, the genetic linkage map file for the mapping experiments `exp1` would be

```
group     marker    position
1         lgm0392   0
1         bnlg1178  47.7
1         ts2       102.7
1         bnlg1884  137.5
1         lgm1234   179.3
1         lgm1465   293.2
2         bnlg1092  0
2         bnlg1017  26.8
2         lgm0701   37.4
2         lgm0653   165
2         bnlg1520  190.8
2         dupssr32  218.1
3         bnlg1904  0
3         bnlg1035  36.6
3         bnlg1063  63.1
3         bnlg1182  140.2
3         dupssr8b  161.2
```

2.1.4 QTLdb

As for GLMdb the QTL database consists in as many plain text files as the number of mapping experiments described in the experiment table. Each file must respect the same naming convention as for GLMdb, i.e the stem name of the file must be the name of the corresponding mapping experiment as defined in the **map** field of the experiment table. Thus if a QTL detection result has been reported for the mapping experiment '`exp1`' we will have a file '`exp1.txt`'. Each file of QTLdb are organized as follows:

- **group** (STRING) : the linkage group name.
- **qtl** (STRING) : the name of the QTL.
- **position** (NUMERIC) : the most probable position of the QTL on the linkage group.
- **qtl.trait.name**(STRING): the name of the trait to which this QTL is related.
- qtl.ci.from(NUMERIC) : the lower bound of the CI of the QTL position.
- qtl.ci.to(NUMERIC) : the upper bound of the CI of the QTL position.
- qtl.ci.lod.decrease(NUMERIC): if the CI is based on a LOD support, use this field to define the decrease of LOD which was used to build the CI.
- qtl.rsquare(NUMERIC): the estimated r-square of the QTL.
- qtl.cross.name(STRING): the name of the cross used to carry out the QTL mapping.
- qtl.cross.type(STRING): the type of the cross used to carry out the QTL mapping.
- qtl.cross.size(INTEGER): the size of the cross used to carry out the QTL mapping.

```
group  qtl   position qtl.ci.lod.decrease  qtl.ci.from  qtl.ci.to  qtl.rsquare qtl.trait.name
1  QTL1  208  1  182  236  0.044  days_to_pollen_shed
1  QTL2  152  1  128  174  0.045  silking_date
2  QTL3  74   1  58   88   0.051  silking_date
2  QTL4  126  1  110  140  0.048  days_to_pollen_shed
3  QTL6  36   1  18   46   0.093  leaf_number
8  QTL5  76   1  60   92   0.21   leaf_number
```

2.2 MetaDB

This program is designed to reformat all the tables of the database into a set of XML files which will be used by the analysis programs.

2.2.1 Command Line Options

Option	Usage	Type	Explanation
`-e,--exp`	required	STRING	Experiment file
`-o,--output`	required	STRING	Output XML directory
`-m,--mapdb`	required	STRING	The GLMdb directory
`-q,--qtldb`	optional	STRING	The QTLdb directory
`-t,--tofile`	optional	STRING	Trait Ontology file
`-d,--mrkdico`	optional	STRING	Marker dictionary file
`--mrkup`	optional	STRING	A file with the marker name to update
`--mrkrm`	optional	STRING	A file with the marker to remove
`--chrm`	optional	STRING	A file with the chromosome to remove

For example, suppose we have created a database which consists in the files '`exp.txt`' (experiment table), '`onto.txt`' (trait ontology), '`dico.txt`' (marker dictionary), and the directories '`glmdb/`' and '`qtldb/`' which contain the marker map and the QTL map tables relative to the mapping experiments specified in '`exp.txt`'. We assume that all the files and directories are included into the same directory, the database directory. Then we can create the XML repository, '`xmldb/`', by invoking the command (in the database directory)

```
>java org.metaqtl.main.MetaDB \
       -e exp.txt -t onto.txt -d dico.txt -m glmdb -q qtldb -o xmldb
```

This creates as many XML files in the '`xmldb/`' directory as the number of mapping experiments. For a given mapping experiment all the information (parameters, markers, QTL, ...) are merged into a single XML file which stem name is the same than the mapping experiment name. The output directory '`xmldb/`' also contains a file named '`ontology.xml`' which represents the trait ontology table (if this last one is not valid, the file is not created). Finally, using the marker dictionary, **MetaDB** converts all the marker names which match a synonym in the marker dictionary to their standard name. If you want to export these XML files into another file format, See Section 5.2 [Xml2A], page 33.

For the option `--mrkup`, the input file format must be a table with 4 columns seperated by a tabulation as follows:

```
Exp1 Chrom1 mrk3 mrk3a
Exp1 Chrom2 mrk4 mrk4b
Exp2 Chrom1 mrk1 mrk1c
...
```

where the first column gives the name of the mapping experiment (must be the same than in the experiment table), the second the name of the chromosome, the third one the current name of the marker to update and the last one the new name of the marker.

For the option `--mrkrm`, the input file is the same than the previous one except that it contains only the first three columns.

Finally, for the option `--chrm`, the input file is the same than the previous one except that it contains only the first two columns.

Note that these three last options have no effect on the raw data file: the rules they define are only applied to the ouput XML data base.

3 Meta Analysis

MetaQTL implements a serie of programs which can be combined to do a complete analysis. Here we give details on each program: how to use them and what they do. We classified the programs into 3 main categories which correspond to the main steps of a complete QTL meta-analysis.

3.1 Consensus map

The first step of the QTL meta-analysis is to intergate all the input genetic marker maps into a single one called the consensus marker map.

3.1.1 InfoMap

3.1.1.1 Method

Before performing the construction of the consensus map, it is important to evaluate how the input maps are connected together. For each linkage group, **InfoMap** displays some descriptive statistics about the marker maps and for each pair of mapping experiments the program looks for common marker sequences. A common marker sequence is a set of at least two common markers for which the order in the two linkage groups is consistent. The marker order is said to be consistent even if the sequence is completely inverted between the two linkage groups.

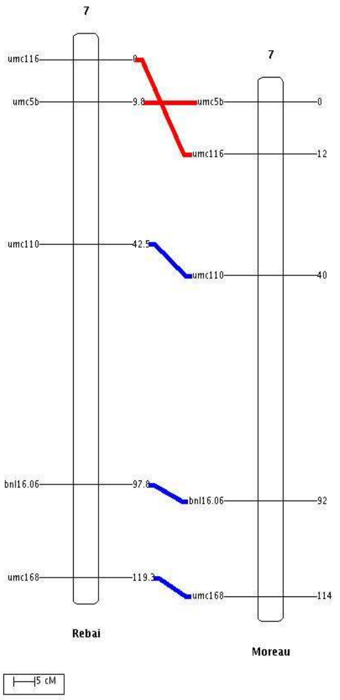

For example, in the above figure the number of common marker sequences is 2. The first one (in red) involved 2 markers (*umc116*, *umc5b*), which order is reversed between the two chromosome, and the second one (in blue) involved 3 markers (*umc110*, *bnl16.06*, *umc168*).

3.1.1.2 Command Line Options

**Option**	**Usage**	**Type**	**Explanation**

`-m,--mapdir`	required	STRING	The directory which contains the XML files.
`-o,--output`	required	STRING	The output file name.
`-t,--mrkth`	optional	INTEGER	The threshold on the occurence of the markers.

For example,

```
%java org.metaqtl.main.InfoMap -m xmldb -t 2 -o info.txt
```

gives information on the mapping experiments which XML files are included in the directory `xmldb` by keeping only markers which are defined in at least 2 mapping experiments.

3.1.1.3 Output

The output of **InfoMap** is a plain text file. For each linkage group, results are introduced by a '>CR' tag followed by the name of the linkage group as follows:

```
>CR 7 Connected=true
#
# Table of the chromosomes
#
1         Ribaut              12        9.67
2         Lubberstedt         9         10.34
3         Moreau              9         15.23
4         Rebai               9         13.26
5         Mechin              9         8.78
#
# Average number of marker per chrom m=9.60
# Average interval marker distance=11.45
#
```

The term '`Connected=true`' means that it exists a path in terms of common markers from any mapping experiment to any other mapping experiment. Otherwise, the value will be set to '`false`'. This insures that for this linkage group the consensus map of the chromosome can be build. Then comes a table which summarizes the number of markers and the average interval marker distance per mapping experiment. It is followed by the table of common markers which gives for each pair of mapping experiments the number of common markers which connects them. It can be viewed as the adjency matrix of the graph which connects the mapping experiments according to the number of common markers they share.

```
#
# Table of the number of common markers
#
# Total number of marker M=37
# Prop of common markers p=0.1650793650793651
#
Chrom             Index     1         2         3         4         5         Total
Ribaut            1                                       1.0                 1.0
Lubberstedt       2                             1.0       2.0       1.0       4.0
Moreau            3                   1.0                 5.0       2.0       8.0
Rebai             4         1.0       2.0       5.0                 3.0       11.0
Mechin            5                   1.0       2.0       3.0                 6.0
```

The two last tables are based on the common marker sequences found for each pair of mapping experiments.

```
#
# Table of the number of common sequences
#
Chrom              Index    1        2        3        4        5
Ribaut             1
Lubberstedt        2                                   1.0
Moreau             3                                   2.0      1.0
Rebai              4                 1.0      2.0               1.0
Mechin             5                          1.0      1.0

#
# Table of proportion of marker involved in common sequences
# The value can be negative depending on the frame of the
# common sequences
#
Chrom              Index    1        2        3        4        5
Ribaut             1
Lubberstedt        2                                   1.0
Moreau             3                                   0.2      1.0
Rebai              4                 1.0      0.2               1.0
Mechin             5                          1.0      1.0
```

In this example, the first table reveals that the mapping experiment “Moreau” and “Rebai” show marker order inconsistency since there are 2 common marker sequences between them. In the second table the proportion of common markers involved in the common marker sequences are reported. This proportion is obtained by summing the number of markers of the common sequences weighted by 1 if the order is the same, -1 otherwise, and dividing this quantity by the total number of common markers. This value lies between -1 and 1. A value of 1 indicates that all the common markers are correclty ordered between the mapping experiments meanwhile a value of -1 indicates that the common marker order is completely reversed between the two mapping experiments. For example, in the above figure the two maps share 5 common markers: 2 are involved in a common sequence which is inverted, and 3 in a common sequence in the same way. Then the proportion of markers involved in common sequences is (3 x 1 + 2 x (-1))/5 = 0.2.

3.1.2 ConsMap

ConsMap is dedicated to the construction of consensus marker map.

3.1.2.1 Method

The method implemented in **MetaQTL** is based on a Weigthed Least Square (WLS) strategy. Contrary to iterative projection procedure, this method makes it possible to integrate all maps in a single step. It is also possible to fix a genetic map as reference.

3.1.2.2 Command Line Options

Option	Usage	Type	Explanation
`-m,--mapdir`	required	STRING	The directory which contains the XML files.
`-o,--outstem`	required	STRING	The output file stem.
`-r,--refmap`	optional	STRING	The XML file of the reference map.
`-t,--mrkthresh`	optional	INTEGER	The threshold on the occurence of the markers.
`-d,--dubfile`	optional	STRING	The file containg a list of markers to ignore.
`--mrkdico`	optional	STRING	The marker dictionary.

For example,

```
%java org.metaqtl.main.ConsMap \
>     -m xmldb -r xmldb/IBM.xml -o consmap
```

builds a consensus linkage map using all the maps in the directory 'xmldb' fixing the map defined in 'xmldb/IBM.xml' as the reference.

3.1.3 Output

The result of **ConsMap** consists in two files:

- '<output_stem>_map.xml' : a XML file which represents the consensus map.
- '<output_stem>_fit.txt' : a plain text file which gives for each chromosome the goodness-of-fit and the standardized residuals between the input maps and the consensus one. Each line begins with an identifier composed by 2 characters followed by its corresponding value.

**Identifier**	**Value**
CR	The name of the linkage group.
NM	The number of distinct markers positioned on the consensus linkage group.
GF	The goodness-of-fit of the consensus linkage group.
PV	The p-value associated to the goodness-of-fit.
DF	The number of degree of freedoms of the residual.
SR	The standardized residuals: first comes the name of the mapping experiment followed by the value of the residual. These values are ordered according to the order of the marker intervals in the linkage group of the mapping experiment.

For example, this file can look like this:

```
CR 7
NM 37
GF 182.0738940381568
PV 1.0
DF 7
SR Lubberstedt 1.6308530669014496E-13
SR Lubberstedt -9.205700855506115E-14
SR Lubberstedt -3.401706740747478E-14
SR Lubberstedt -1.049889614734477E-13
SR Lubberstedt 1.2471027908772377E-13
SR Lubberstedt -2.9725343354129593E-13
SR Mechin -4.797069139413729E-15
SR Mechin 5.484351947823543E-14
SR Mechin 6.830428378396427E-15
SR Mechin 0.7995364207820779
SR Mechin 0.3047608429054208
SR Mechin -6.98290111003849E-14
SR Moreau 9.661620158868837E-15
SR Moreau 10.85507243048007
SR Moreau -2.1096760232982783
SR Moreau -0.002216097065843051
...
```

3.2 QTL Projection

3.2.1 Method

QTL projection consists in positioning the QTL located on a given map (the original map) onto another one (the reference map). To illustrate the basic principle of QTL projection, let's assume that the flanking markers of a given QTL location in the original map are also included in the reference. Then the QTL can be projected into the flanking marker interval of the reference map by using a simple homothetic rule. If the QTL have a confidence interval (CI) defined

on the orignal map then it is resized on the reference map according to a scaling factor which takes into account the marker interval distance variations between the original and the reference map. This factor is computed by summing, over all the common marker sequences, the ratio of the marker interval distances between the reference and the original map weighted by the probability that the QTL lies in these intervals.

3.2.2 QTLProj

QTLProj implements a dynamic algorithm in order to find the optimal context to do the projection. An optimal context consists in a pair of common marker which flanks the QTL in the original map and for which the distance estimate is consistent between the maps. Two parameters control the behaviour of the algorithm to find such a configuration: the minimal value of the ratio of the flanking marker interval distances and the minimal p-value obtained by testing the homogeneity of the flanking marker interval distances between the original and the reference map.

3.2.2.1 Command Line Options

**Option**	**Usage**	**Type**	**Explanation**
`-m,--map`	required	STRING	The reference map (XML format).
`-q,--qtl`	required	STRING	The directory containing the original maps (XML format).
`-o,--output`	required	STRING	The output file stem.
`-r,--ratio`	optional	DOUBLE	The minimal ratio between 0 and 1 (default is 0.5)
`-p,--pval`	optional	DOUBLE	The minimal p-value between 0 and 1 (default is 0.01).
`--rmrk`	optional	STRING	A list of marker to ignore during the projection
`--rqtl`	optional	STRING	A list of QTL to be excluded from the projection

For example,

```
%java org.metaqtl.main.QTLProj \
>     -m reference.xml -q xmldb -o projection -r 0.25 -p 0.05
```

projects the QTL located onto the maps included in the directory '`xmldb`' on the reference map '`reference.xml`'. Only the QTL for which it is possible to find a pair of flanking markers for which the interval distance is not reduced by more than a factor of 0.25 or for which the p-value of the homogeneity test of equal distances is greater than 0.05 will be projected. If the `-v` option is activated, the programs will return as many WARNING outputs as the number of QTL for which the projection is not possible.

3.2.2.2 Output files

QTLProj output consists in two XML files:

- '`<output_stem>_map.xml`' : This file contains both the markers and the projected QTL locations on the reference map (plus eventually the QTL previously defined on the reference).
- '`<output_stem>_qtl.xml`' : This file contains only the QTL which have been projected on the reference map (plus eventually the QTL previously defined on the reference).

3.3 QTL Clustering

Here we want to address the following question: How many “real” QTL do the QTL detected in the different mapping experiments represent - one, two, three, four,... or as many as the number detected throughout the studies ? The meta-analysis of QTL can be viewed as a clustering procedure. To do so, **MetaQTL** implements tow kinds of clustering algorithm. Whatever the procedure used to perform the clustering, the QTL locations are assumed to be normally distributed around their true locations with variances which can be derived from the reported CI or r-square values. This Gaussian and unbiased approximation comes from the classical asymptotic Gaussian distribution of the maximum-likelihood estimation of the parameters.

3.3.1 ClustQTL

3.3.1.1 Method

ClustQTL implements a clustering procedure based on a Gaussian mixture model which parameter estimates are obtained by applying a EM-algorithm.

3.3.1.2 Command Line Options

**Option**	**Usage**	**Type**	**Explanation**
`-q,--qtlmap`	required	STRING	The map with the QTL to clusterize (XML format).
`-o,--output`	required	STRING	The output file stem.
`-t,--tonto`	optional	STRING	The trait ontology.
`-k,--kmax`	optional	INTEGER	The maximal number of clusters.
`-c,--chr`	optional	STRING	The name of the chromosome on which to perform the meta-analysis.
`--cimode`	optional	INTEGER	The CI computation mode.
`--cimiss`	optional	INTEGER	The imputation mode for missing CI.
`--emrs`	optional	INTEGER	the number of random starting points for the EM algorithm
`--emeps`	optional	DOUBLE	the convergence threshold for the EM algorithm

The option `--cimode` controls the mode of computation of the variances of the QTL. There are four modes:

- 1 : the variances are computed according to the avalaible information: from the CI if defined, otherwise from the r-square value.
- 2 : the variances are only computed for the QTL locations for which a CI is reported.
- 3 : the variances are computed using the r-square values.
- 4 : the variances are obtained by taking the maximum value between the variance derived from the CI and/or from the r-square.

The `--cimiss` defines how to deal with QTL for which no variance can be computed. There are two possibilities:

- 1 : the mean of the estimated variances is attributed to QTL with no variance defined.
- 2 : the QTL with no variance defined are ignored.

3.3.1.3 Output

The output of **ClustQTL** is divided into 3 plain text files:

- '<output_stem>_res.txt' : this files contains a summary of the results of the clustering for each linkage group. The file is organized as follows

**Identifier**	**Value**
CR	The name of the linkage group.
TR	The trait name following by the number of related QTL on the chromosome.
QT	A QTL with its identifier, its name, its position on the chromosome and its estimated standard deviation.
CL	Indicates the beginning of a clustering result. It is followed by the number of QTL involved in the clustering, the number of clusters, the log-likelihood and the complete log-likelihood of this clustering.
CC	The name of a model choice criterion followed by its value.
CP	This tag recovers four kinds of entry: • PI : the weights of each cluster (i.e the mixing proportions in the mixture model). • MU : the QTL location estimates (i.e the centroids of each cluster). • CI : the 95% confidence intervals of the QTL location estimates. • Z : the QTL cluster membership probabilities: first comes the identifier of the QTL and then the probabilities.

For example,

```
CR 3
TR FloweringTime 10
QT 0 Lubberstedt_1997_HT_7 106.35 7.9
QT 1 Cardinal_2001_HT_5 90.76 7.91
QT 2 qplht107 150.02 5.28
QT 3 Cardinal_2001_HT_6 51.03 7.26
QT 4 qplht106 107.46 1.3
QT 5 Groh_1998_HT_2 61.03 17.15
QT 6 Bohn_1996_HT_2 66.81 4.26
QT 7 Lubberstedt_1997_HT_6 80.67 3.04
QT 8 qplht105 75.45 4.61
QT 9 Blanc_SDflofch3 148.15 15.05
QT 10 Blanc_FXflofch3 135.27 21.68
CL 10 2 -462.46 -445.55
CC AIC 930.91
CC BIC 935.11
CP MU 88.87 148.91
CP PI 0.73 0.27
CP CI 3.82 3.76
CP Z 0 1 0
CP Z 1 1 0
CP Z 2 0 1
CP Z 3 1 0
CP Z 4 1 0
CP Z 5 1 0
CP Z 6 1 0
CP Z 7 1 0
CP Z 8 1 0
CP Z 9 0 1
CP Z 10 0.1 0.9
...
```

- '<output_stem>_crit.txt' : this file summarizes the values of the model choice criteria. For example,

```
Chromosome          Trait     K         Criterion Value     Delta     Weight
3         FT        1         AIC       1969.57   1654.71   0
3         FT        2         AIC       930.91    616.05    0
3         FT        3         AIC       445.55    130.69    0
3         FT        4         AIC       364.46    49.6      0
3         FT        5         AIC       314.86    0         0.51
3         FT        6         AIC       315.54    0.68      0.36
3         FT        7         AIC       317.92    3.06      0.11
3         FT        8         AIC       322.44    7.58      0.01
3         FT        9         AIC       326.44    11.58     0
3         FT        10        AIC       330.44    15.58     0
3         FT        30        AIC       361.77    46.92     0
3         FT        1         BIC       1970.97   1643.5    0
3         FT        2         BIC       935.11    607.64    0
3         FT        3         BIC       452.55    125.08    0
3         FT        4         BIC       374.27    46.8      0
3         FT        5         BIC       327.47    0         0.84
3         FT        6         BIC       330.95    3.48      0.15
3         FT        7         BIC       336.14    8.67      0.01
3         FT        8         BIC       343.46    15.99     0
3         FT        9         BIC       350.26    22.79     0
3         FT        10        BIC       357.06    29.59     0
3         FT        30        BIC       403.81    76.34     0
```

The first column indicates the name of the chromosome, the second one the name of the trait, the third the number of clusters, the fourth the name of the criterion and the three last ones give respectively the criterion value, its rescaled value and the “weight of evidence”.

- ‘<output_stem>_model.txt’: This file gives the optimal number of QTL location according to the model choice criteria. The file is organized as a table with 4 columns. The first column indicates the name of the criterion, the second one the name of the chromosome, the third one the name of the trait and the last one the optimal number of QTL. For example,

```
Criterion Chromosome          Trait     Model
AIC       3         FT        2
AIC       10        FT        4
AIC       5         FT        4
AIC       7         FT        5
AIC       2         FT        4
AIC       9         FT        3
AIC       4         FT        3
AIC       8         FT        5
AIC       6         FT        3
AIC       1         FT        5
BIC       3         FT        2
BIC       10        FT        3
BIC       5         FT        4
BIC       7         FT        5
BIC       2         FT        4
BIC       9         FT        3
BIC       4         FT        3
BIC       8         FT        5
BIC       6         FT        3
BIC       1         FT        5
```

3.3.2 QTLTree

3.3.2.1 Method

Another way to clusterize the observed QTL is to use standard hierarchical clustering procedures. **QTLTree** implements two kinds of hierarchical clustering algorithm :

- Average group linkage : once cluster of QTL are formed, they are represented by their mean values, that is, their mean location, and inter-cluster distance is defined as the distance

between two mean values. In the average group linkage method, the two clusters Q1 and Q2 are merged such that, after merging, the average pairwise distance within the newly formed cluster, is minimum. Suppose we label the new cluster formed by merging clusters Q1 and Q2, as Q3. Then D(Q1,Q2) , the distance between clusters Q1 and Q2 is computed as D(Q1,Q2) = Average {d(QTLi,QTLj) : where QTL i and j are in cluster Q3, the cluster formed by merging clusters Q1 and Q2}. At each stage of hierarchical clustering, the clusters Q1 and Q2 , for which D(Q1,Q2) is minimum, are merged. The distance used here is the mahalanobis distance.

- Ward's method : Ward (1963) proposed a clustering procedure seeking to form the partitions Qn, Qn-1,........,Q1 in a manner that minimizes the loss of information associated with each grouping, and to quantify that loss in a form that is readily interpretable. At each step in the analysis, the union of every possible cluster pair is considered and the two clusters whose fusion results in minimum increase in 'information loss' are combined. Usually, information loss is defined in terms of a error sum-of-squares like criterion, called the target function. Here the target function is defined as the loglikelihood of being one "actual" QTL underlying the distribution of the observed QTL locations within the cluster.

3.3.2.2 Command Line Options

**Option**	**Usage**	**Type**	**Explanation**
`-q,--qtlmap`	required	STRING	The map with the QTL to clusterize (XML format).
`-o,--output`	required	STRING	The output file.
`-m,--mode`	optional	INTEGER	The clustering mode (default is 2).
`-t,--tonto`	optional	STRING	The trait ontology.
`--cimode`	optional	INTEGER	The variance computation mode.
`--cimiss`	optional	INTEGER	The imputation mode for missing variances.

The option `-m` (or `--mode`) allows user to switch between the two possible clustering algorithms:

- 1 : Average group linkage.
- 2 : Ward's metod.

The options `--cimode` and `--cimiss` works as for **QTLClust**.

3.3.2.3 Output

The output of **QTLTree** consists in one plain text file. The file is organized as follows:

**Identifier**	**Value**
CR	The name of the linkage group.
TR	The name of the trait followed by the number of related QTL on the chromosome.
QT	A QTL involved in the clustering with its identifier, its name, its most probable position on the chromosome and its estimated standard deviation.
HC	The tree obtained by the clustering algorithm in Newick's format.

For example,

```
CR 10
TR FT 16
QT 0 Ribaut_1996_DPS_6 8.02 7
QT 1 Bohn_2000_DPS_12 51.68 4.87
QT 2 Poupard_2001_DPS_13 40.02 3.65
QT 3 Mechin_2001_HT_5 71 4.26
QT 4 Lubberstedt_1997_HT_20 59.14 3.65
QT 5 Groh_1998_HT_7 100.01 12.2
QT 6 qplht127 52.39 5.25
QT 7 Rebai_1997_SD_5 66.51 5.17
QT 8 Blanc_DFflofch10 61.57 2.55
QT 9 Rebai_1997_SD_25 54.5 12.46
QT 10 Rebai_1997_SD_19 62.14 10.64
QT 11 Blanc_FXflofch10 58.17 3.57
QT 12 Ribaut_1996_SD_6 6.78 10.83
QT 13 Rebai_1997_SD_33 59.96 9.73
QT 14 Rebai_1997_SD_12 49.04 14.59
QT 15 Blanc_SFflofch10 53.13 3.32
HC ((0:0.16,12:0.16):87.85,((((((1:0.24,((6:0.06,15:0.06):0.11,9:0.11):0.24):0.4,14:0.4)
HC :7.43,(((4:0.04,13:0.04):0.16,11:0.16):1.11,(8:0.01,10:0.01):1.11):7.43):15.64,
HC (3:2.43,7:2.43):15.64):24.56,5:24.56):40.9,2:40.9):87.85);
```

4 Visualization

MetaQTL provides some utilities that read the input or the output of the analysis programs and reformat it as images. These images can then be visualized using standard image viewer or editor.

4.1 MapView

MapView is designed to draw markers and QTL for one or several linkage groups, depending on drawing parameters.

4.1.1 Command Line Options

Option	Usage	Type	Explanation
`-m,--mapfile`	required	STRING	The XML file for the genetic map.
`-c,--chrom`	required	STRING	The name of the chromosome(s) to display.
`-o,--output`	required	STRING	The output file stem.
`-q,--withqtl`	optional	INTEGER	Draw also QTL.
`-t,--tonto`	optional	STRING	The XML file for the trait ontology
`-p,--parfile`	optional	STRING	The drawing parameter file.
`--qmode`	optional	INTEGER	QTL representation mode (0=bar,1=arrow)
`--tree`	optional	STRING	a QTL tree (obtained with QTLTree);
`--img`	optional	STRING	The format of the image (use –help for possible values).

For example,

```
%java org.metaqtl.main.MapView \
>     -c 1,2 -m map.xml -q -o figure --img jpeg
```

draws the chromosome 1 and 2 with both markers and QTL of the given map '`map.xml`' and outputs the result in '`figure.jpeg`'. This also creates a file named '`figure.par`' which gives the values of the drawing paremeters. This file can be modified and reused to create another image by using the `-p` or `--par` option

```
%java org.metatqtl.main.MapView \
>     -c 1,2 -m map.xml -q -o figure -p figure.par --img jpeg
```

The next section gives more details on how to deal with the drawing parameters.

4.1.2 Drawing Parameters

Several parameters allow user to put in form the figure. The next figure illustrates the role of each parameter.

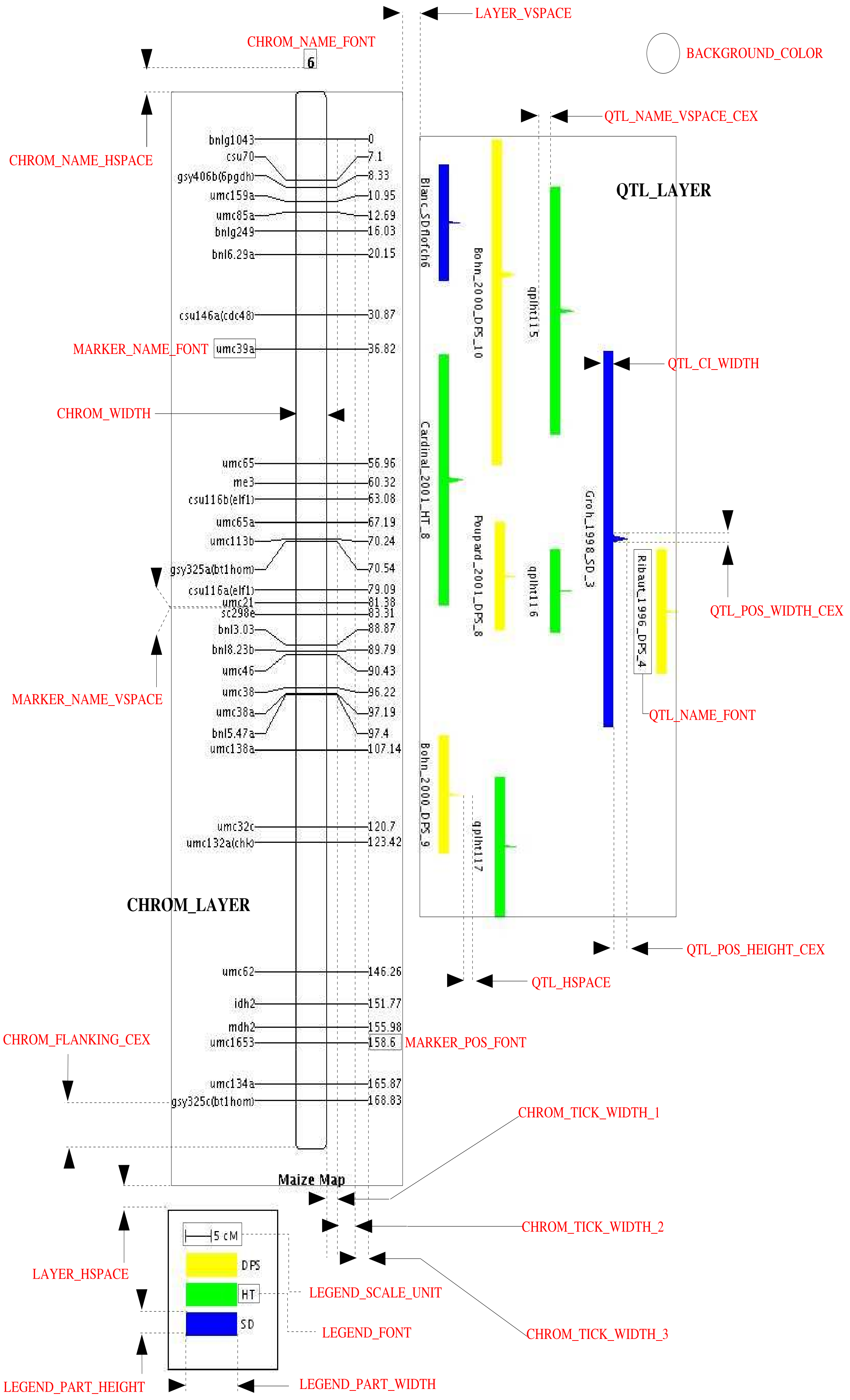

LAYER_VSPACE
CHROM_NAME_FONT
BACKGROUND_COLOR
6
QTL_NAME_VSPACE_CEX
CHROM_NAME_HSPACE
QTL_LAYER
bnlg1043 0
csu70 7.1
gsy406b(6pgdh) 8.33
umc159a 10.95
umc85a 12.69
bnlg249 16.03
bnl6.29a 20.15
csu146a(cdc48) 30.87
MARKER_NAME_FONT
umc39a 36.82
QTL_CI_WIDTH
CHROM_WIDTH
umc65 56.96
me3 60.32
csu116b(elf1) 63.08
umc65a 67.19
umc113b 70.24
gsy325a(bt1hom) 70.54
csu116a(elf1) 79.09
umc21 81.38
sc298e 83.31
bnl3.03 88.87
bnl8.23b 89.79
umc46 90.43
umc38 96.22
umc38a 97.19
bnl5.47a 97.4
umc138a 107.14
umc32c 120.7
umc132a(chk) 123.42
QTL_POS_WIDTH_CEX
MARKER_NAME_VSPACE
QTL_NAME_FONT
Blanc_SDflofch6
Bohn_2000_DPS_10
qplht115
Cardinal_2001_HT_8
Groh_1998_SD_3
Poupard_2001_DPS_8
qplht116
Ribaut_1996_DPS_4
Bohn_2000_DPS_9
qplht117
CHROM_LAYER
QTL_POS_HEIGHT_CEX
QTL_HSPACE
umc62 146.26
idh2 151.77
mdh2 155.98
umc1653 158.6
CHROM_FLANKING_CEX
MARKER_POS_FONT
umc134a 165.87
gsy325c(bt1hom) 168.83
CHROM_TICK_WIDTH_1
Maize Map
CHROM_TICK_WIDTH_2
5 cM
DPS
HT
SD
LAYER_HSPACE
LEGEND_SCALE_UNIT
LEGEND_FONT
CHROM_TICK_WIDTH_3
LEGEND_PART_HEIGHT
LEGEND_PART_WIDTH

The paremeter file is organized as follows:

```
#
# Graphical Parameters
#
# CHROMOSOME
#
CHROM_DISTANCE_SCALE=5.0
CHROM_FLANKING_CEX=0.1
CHROM_NAME_FONT=Utopia Regular:BOLD:12
CHROM_NAME_HSPACE=20.0
CHROM_TICK_WIDTH_1=10.0
CHROM_TICK_WIDTH_2=20.0
CHROM_TICK_WIDTH_3=10.0
CHROM_WIDTH=30.0
MAKER_NAME_VSPACE=1.0
MARKER_NAME_FONT=Utopia Regular:PLAIN:10
MARKER_POSITION_FONT=Utopia Regular:PLAIN:10
#
# QTL
#
QTL_CI_WIDTH=10.0
QTL_CI_WIDTH_CEX=0.25
QTL_HSPACE=10.0
QTL_NAME_FONT=Courier:PLAIN:10
QTL_NAME_VSPACE_CEX=1.0
QTL_POS_HEIGHT_CEX=0.01
QTL_POS_WIDTH_CEX=1.5
QTL_VSPACE=20.0
#
# LEGEND
#
LEGEND_BOX_CEX=0.5
LEGEND_FONT=Verdana:PLAIN:10
LEGEND_HSPACE=5.0
LEGEND_PART_HEIGHT=20.0
LEGEND_PART_WIDTH=50.0
LEGEND_SCALE_UNIT=5
#
# OTHER
#
BACKGROUND_COLOR=ffffff
LAYER_HSPACE=20.0
LAYER_VSPACE=20.0
WITH_CHROM_NAME=true
WITH_LEGEND=true
WITH_MAP_NAME=true
WITH_MARKER_NAME=true
WITH_QTL_NAME=true
WITH_MARKER_POSITION=true
```

A line which begins by the character '#' is ignored by the program. Each drawing parameter is specified by its own name directly followed by a sign '=' and its value. For the parameters which specify a color (those which end by '_COLOR') the value must be a RGB code in hexadecimal (e.g red is ff0000, green 00ff00, blue 0000ff, white ffffff, black 000000, and etc...). For the parameters which specify a font (those which end by '_FONT') the value must be a string composed by 3 tokens separated by a ':'. The first token gives the name of the font, the second one the style of the font (among 'PLAIN,BOLD' and 'ITALIC') and the last one indicates the size of the font. The parameter file generated by **MapView** ends by a list of the available fonts on your system. If you do not use a font name in this list, then **MapView** will use the default font. The parameters which start by 'WITH_' allow the user to specify if the corresponding elements of the figure must

be drawn. The possible values for these parameters are either '`true`' (draw) and '`false`' (not to draw). For example if you set '`WITH_MARKER_POSITION=false`' the marker positions along the chromosome wont' be drawn. When the QTL are added to the figure (option `-q` with **MapView**) they are colored according to their trait group. The colors used to display the QTL are also specified in the parameter file generated by the program. For example, let's assume that the QTL belongs to 3 trait groups. The parameter file will then contain the following lines:

```
#
# COLOR PALETTE
#
QTL_COLOR_1=ffff00
QTL_COLOR_2=00ff00
QTL_COLOR_3=0000ff
```

where `QTL_COLOR_i` is the color of the group `i`. You can use these lines to modify the default colors.

4.2 MMapView

MMapView is designed to draw markers and QTL for one linkage group for several genetic maps. It helps to visualize common markers between maps but also to display marker interval distance heterogeneities.

4.2.1 Command Line Options

**Option**	**Usage**	**Type**	**Explanation**
`-m,--mapdir`	required	STRING	The XML file/directory for the genetic map(s).
`-c,--chrom`	required	STRING	The name of the chromosome to display.
`-o,--output`	required	STRING	The output file stem.
`-r,--refmap`	optional	STRING	The XML file for the reference map.
`-q,--withqtl`	optional	INTEGER	The threshold on the occurence of the markers.
`-t,--tonto`	optional	STRING	The XML file for the trait ontology
`--htest`	optional	BOOLEAN	Test distance homogeneity between the maps and the reference (only with -r).
`--hth`	optional	DOUBLE	The threshold of the p-value of the homogeneity test between the maps and the reference (only with -r and -h). The value must be between]0,1[. A value of 0 or 1 codes for a gradient view.
`--mrkt`	optional	INTEGER	Threshold on the occurence of the markers.
`-p,--parfile`	optional	STRING	The drawing parameter file.
`--img`	optional	STRING	The format of the image (use –help for possible values).

Suppose we have a set of genetic maps in XML format in the directory '`xml`' and that all the maps have a chromosome called 1. Then we can use **MMapView** to display all the chromosomes in a single figure as follows:

```
%java org.metaqtl.main.MMapView \
>     -c 1 -m xml -o figure
```

This dumps the image into a file 'figure_1.jpeg' and the parameter file into 'figure_1.par'. We can also represent the same chromosomes but with only the markers which have observed at least in two distinct chromosomes by using the option --mrkt:

```
%java org.metaqtl.main.MMapView \
>     -c 1 -m xml -o figure --mrkt 2
```

Now, suppose we want to draw the same chromosomes but also to represent the eventual interval marker distance heterogenities relatively to a reference chromosome defined in file 'reference.xml'. Then we use the command,

```
%java org.metaqtl.main.MMapView \
>     -c 1 -m xml -r reference.xml --htest -o figure
```

4.2.2 Drawing parameters

MMapView use the same drawing parameters than **MapView** plus some extra parameters. If you want to display common marker links between adjacent chromosomes set 'WITH_COMMON_

MARKER' to 'true'. The width of the lines which connect the common markers can be modified by using the parameter 'COMMON_STROKE_WIDTH'. This is illustrated in the next figure.

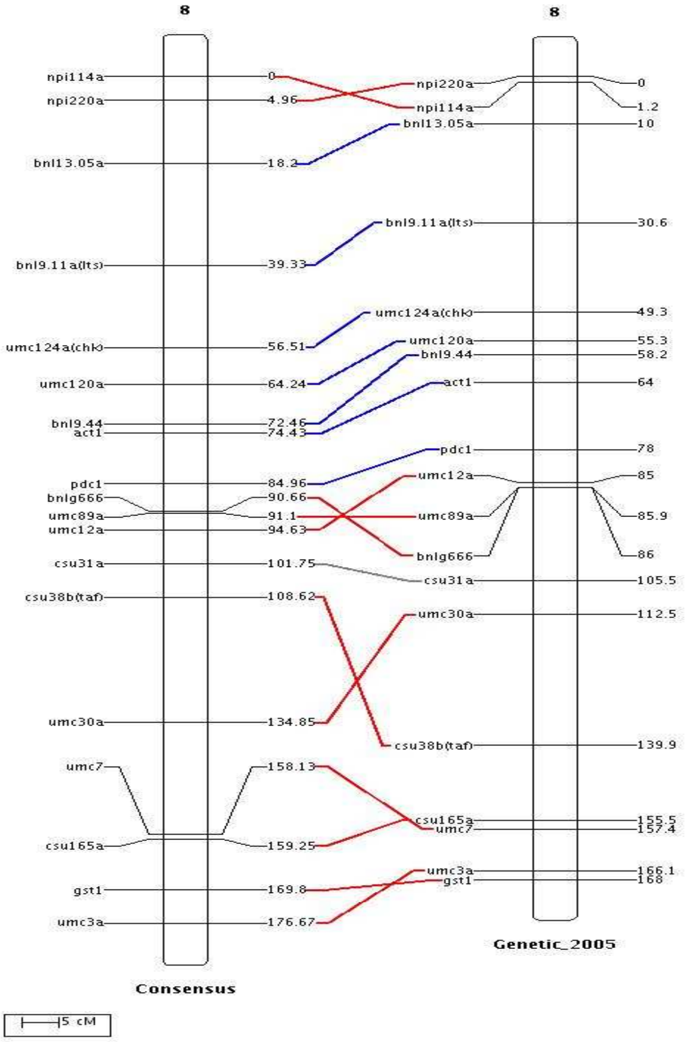

The link between common markers can also be painted depending on the way of the common sequences in which they are involved. For example, in the above figure the common markers which are involved in common sequence correctly ordered between the two chromosomes have their links painted in blue, otherwise their links are painted in red. Note that one marker is not involved in a common sequence and its link is painted in gray. These colors can be changed by using the parameters 'SINGLE_COMMON_COLOR', 'POS_COMMON_COLOR' and 'NEG_COMMON_COLOR'. For example, to create the previous figure we added the following lines to the parameter file:

```
#
# DRAW COMMON MARKER LINKS
#
WITH_COMMON_MARKER=true
COMMON_STROKE_WIDTH=2.0
SINGLE_COMMON_COLOR=808080
POS_COMMON_COLOR=0000ff
NEG_COMMON_COLOR=ff0000
```

When **MMapView** is used to visualize the test of homogeneity between a reference chromosome and several other chromosomes, you can parametrize both the number of bin colors to represent the probabilities and the type of gradient. For example, to create the following figure

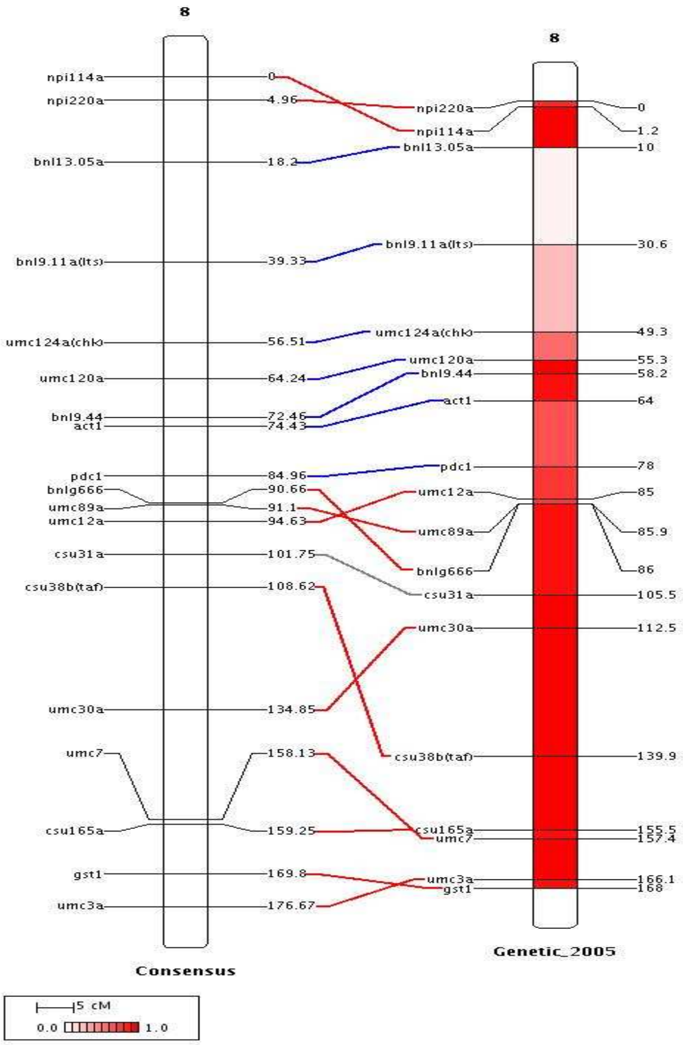

we added the extra parameters:

```
#
# PROBABILITY GRADIENT
#
PROBA_BIN=20
PROBA_FROM_COLOR=ffffff
PROBA_TO_COLOR=ff0000
```

This means that the interval between 0 and 1 is divided into 20 intervals and the gradient starts in white (ffffff) and ends in red (ff0000). Note that the probability gradient is represented into the legend box.

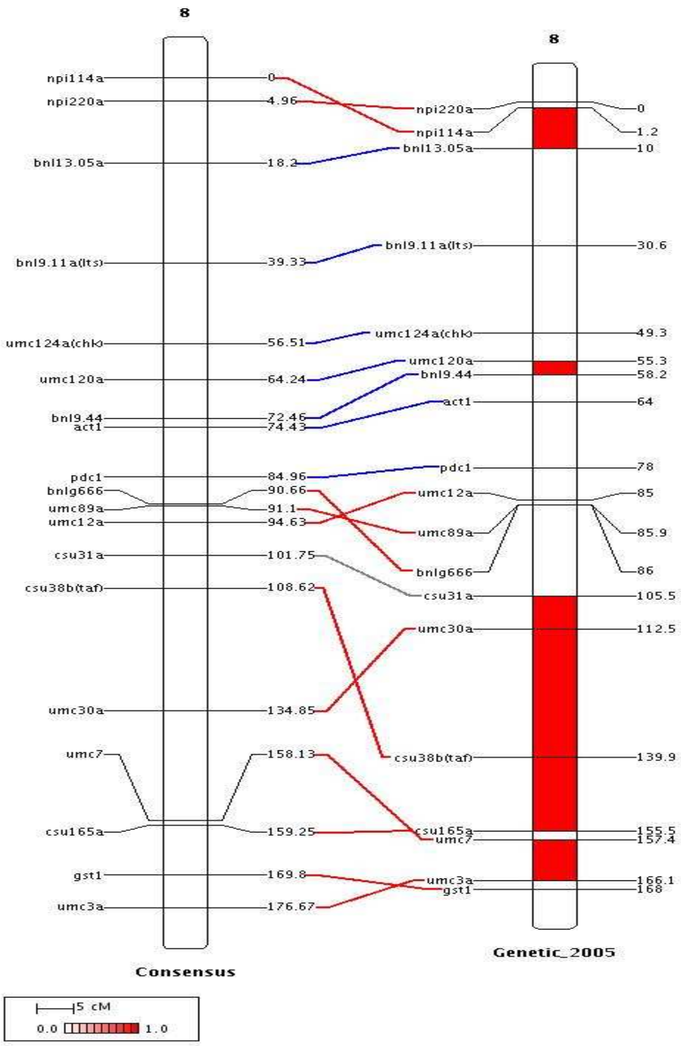

Finally the chromosomes displayed in the figure can be aligned either relatively to the first marker of each chromosome, `CHROM_ALIGN_MODE=1`, or relatively to the first common marker between the first chromosome (the left one) and the other ones, `CHROM_ALIGN_MODE=0`.

4.3 MQTLView

MQTLView is designed to depict the result of the QTL clustering for one or several chromosomes on a given map.

4.3.1 Command Line Options

Option	Usage	Type	Explanation
`-m,--map`	required	STRING	The XML file for the genetic map.
`-c,--chrom`	required	STRING	The name of the chromosome (or a list separated by comma) to display.

`-o,--output`	required	STRING	The output file stem.
`-r,--clust`	optional	STRING	The file of the clustering result.
`-b,--best`	optional	STRING	The file of the best clustering (only with -c).
`-t,--tree`	optional	STRING	The file of the hierarchical clustering result.
`--mode`	optional	INTEGER	The mode of representation (0 or 1 and only with -c and -b)
`--trait`	optional	STRING	The name of the trait.
`-p,--parfile`	optional	STRING	The drawing parameter file.
`--img`	optional	STRING	The format of the image (use –help for possible values).

MQTLView provides several ways to represent the results of the QTL clustering depending on the algorithm used to perform it. Suppose we have first done a clustering using **QTLClust** See Section 3.3 [QTL Clustering], page 13, which produced the result file '`clust_res.txt`'. From the output file '`clust_model.txt`' generated by **QTLClust** we have derived the file '`clust_best.txt`' which gives for a given trait the best clustering model for each chromosome (according to a given model choice criterion):

```
Chromosome      Trait   Model
3       FT      5
10      FT      4
5       FT      4
7       FT      5
2       FT      4
9       FT      2
4       FT      3
8       FT      5
6       FT      3
1       FT      2
```

Then, we can run **MQTLView** to visualize the result, for example on chromosome 8 and 9 together, by invoking the command

```
%java org.metaqtl.main.MQTLView \
>     -c 8,9 -m map.xml -r clust_res.txt -b clust_best.txt -o figure --img jpeg
```

By default the figure is created with the mode `--mode` 1 which leads to something like that

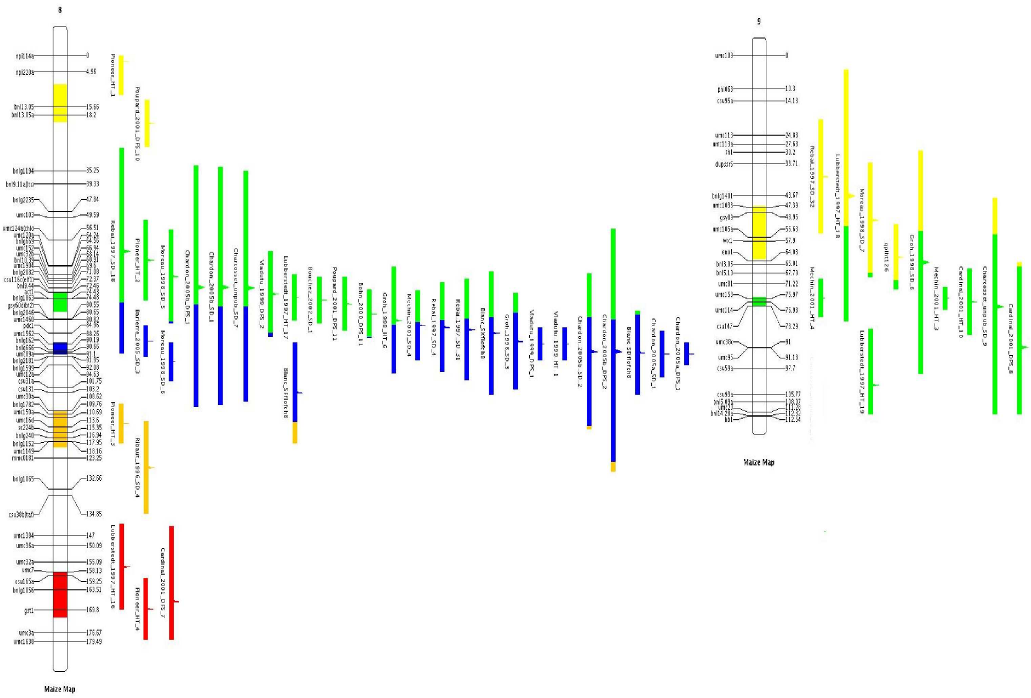

Now, if you set the option `--mode` to ‘0’, i.e

```
%java org.metaqtl.main.MQTLView \
>     -c 8,9 -m map.xml -r clust_res.txt -b clust_best.txt -o figure --img jpeg --mode 0
```

you will obtain the following representation

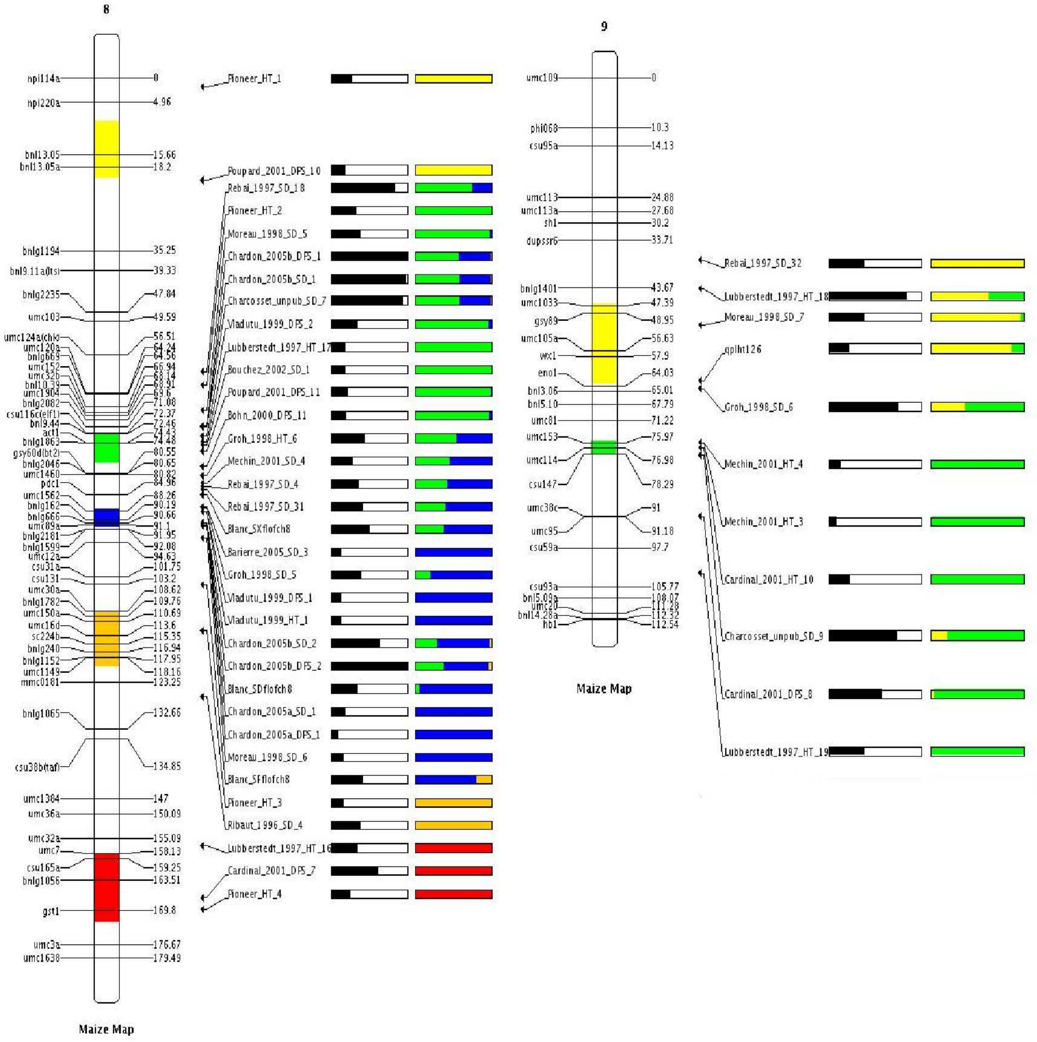

where the first bar which follows the name of the QTL corresponds to the relative CI of the QTL (i.e. that the more the bar is filled, the larger the CI is). The second bar gives the membership probabilities of the QTL according to the meta-QTL model.

If the clustering have been done via **QTLTree**, the result file obtained '`tree.txt`' can be passed to **MQTLView** as follows,

```
%java org.metaqtl.main.MQTLView \
>     -c 3 -m map.xml -t tree.txt -o figure --img jpeg
```

In this case **MQTLView** draws the tree obtained by the hierarchical clustering procedure as illustrated in the next figure.

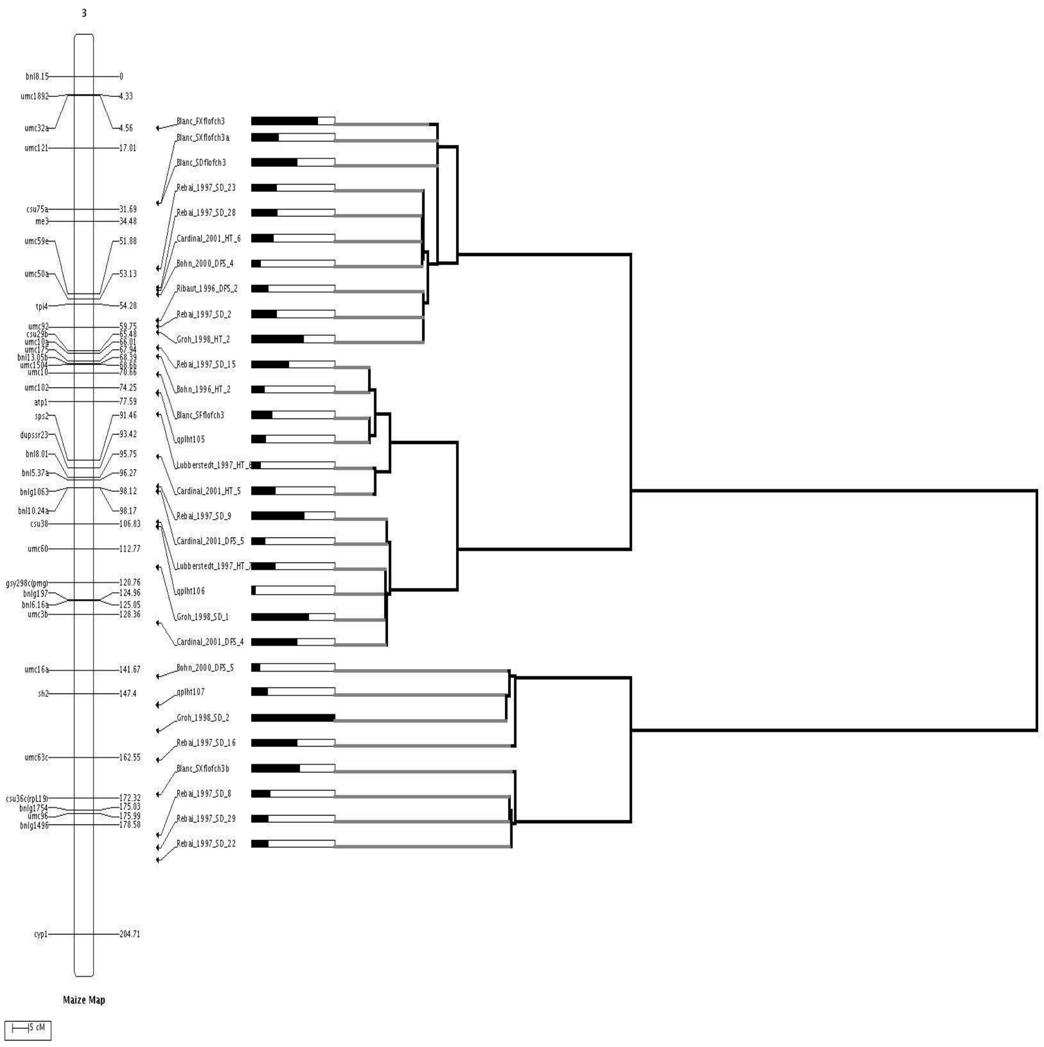

Finally the results from **QTLClust** and **QTLTree** can be visualized together as shown bellow.

```
%java org.metaqtl.main.MQTLView \
>     -c 3 -m map.xml -r clust_res.txt -b clust_best.txt -t tree.txt -o figure --img jpeg
```

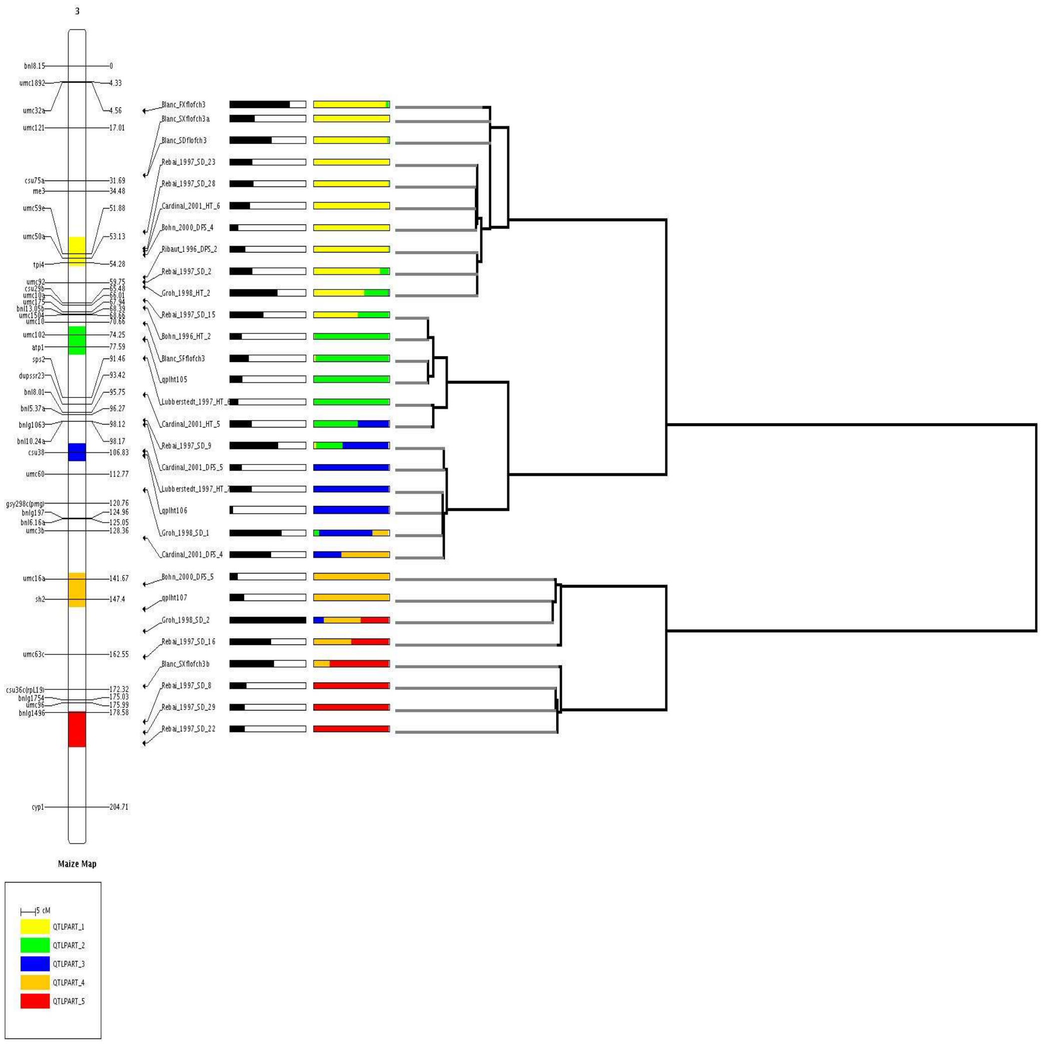

5 Utilities

MetaQTL provides some other programs to deal with file formatting or to extract usefull information from the result files.

5.1 A2Xml

A2Xml is dedicated to convert plaint text files into XML files compliant with the analysis programs of **MetaQTL**.

5.1.1 Command Line Options

**Option**	**Usage**	**Type**	**Explanation**
`-i,--ifile`	required	STRING	The file to convert in XML.
`-x,--xmlfile`	required	STRING	The output XML file.
`-t,--type`	required	STRING	The type of the input file.
`-f,--format`	optional	STRING	The format of the input file.

Presently, the command `--type` have two possible values:

- `map` : the input file is a marker and/or QTL map.
- `onto` : the input file is an ontology.

```
%java org.metatqtl.main.A2Xml \
>     -i map.txt -x map.xml -t map -f chart
```

The command `-f,--format` allows user to convert into XML different input file formats. This option works only when `-t,--type` is set to `map`. In this case 3 different input file formats can be used:

- `tab` : the map is in tabulated format as for the GLMdb tables See Section 2.1.3 [GLMdb], page 5.
- `mch` : the map is formatted like a MapChart (Voorrips 2002) input file.
- `mmk` : the map is formatted like a MapMaker (Lander 1987) output.

For example, suppose we have a map file '`map.txt`' in tabulated format as follows,

```
group   marker  position
1       umc11   0.0
1       gsy27   18.0
1       umc67   54.0
1       umc58   75.0
1       umc83a  115.0
1       umc66b  140.0
1       gsy56   153.0
1       umc161  168.0
1       umc84   189.0
1       bnl6.32 198.0
```

Then we can convert it into a XML file using the command

```
%java org.metatqtl.main.A2Xml \
>     -i map.txt -x map.xml -t map -f tab
```

This creates a file named '`map.xml`' which looks like this

```
<?xml version="1.0" encoding="UTF-8"?>
<genome-map name="">
 <linkage-group name="1">
  <locus name="umc83a" type="M" position="115.0"/>
  <locus name="bnl6.32" type="M" position="198.0"/>
  <locus name="gsy27" type="M" position="18.0"/>
  <locus name="umc58" type="M" position="75.0"/>
  <locus name="umc161" type="M" position="168.0"/>
  <locus name="umc66b" type="M" position="140.0"/>
  <locus name="umc11" type="M" position="0.0"/>
  <locus name="gsy56" type="M" position="153.0"/>
  <locus name="umc67" type="M" position="54.0"/>
  <locus name="umc84" type="M" position="189.0"/>
 </linkage-group>
</genome-map>
```

The XML file will be the same if instead using a tabulated format we have used a MapChart format as input

```
group 1
umc11 0
gsy27 18
umc67 54
umc58 75
umc83a 115
umc66b 140
gsy56 153
umc161 168
umc84 189
bnl6.32 198
```

and the command

```
%java org.metatqtl.main.A2Xml \
>     -i map.txt -x map.xml -t map -f chart
```

or if we have used a MapMaker like format

```
*1
>map
===========================================================================
Map:
  Markers         Distance
   1  umc11         18 cM
   2  gsy27         36 cM
   3  umc67         21 cM
   4  umc58         40 cM
   5  umc83a        25 cM
   6  umc66b        13 cM
   7  gsy56         15 cM
   8  umc161        21 cM
   9  umc84          9 cM
   10  bnl6.32     ----------
                198 cM 10 markers   log-likelihood=
===========================================================================
```

and the command

```
%java org.metatqtl.main.A2Xml \
>     -i map.txt -x map.xml -t map -f mm
```

5.2 Xml2A

Xml2A does exactly the reverse job of **A2Xml**: it takes as input a XML file and convert it into a plain text output file according to the format specified by the user.

5.2.1 Command Line Options

Option	Usage	Type	Explanation
`-o,--outfile`	required	STRING	The output file.
`-x,--xmlfile`	required	STRING	The input XML file.
`-t,--type`	required	STRING	The type of the input file.
`-f,--format`	optional	STRING	The format of the input file.

5.3 QTLClustInfo

When a QTL clustering has been done using **QTLClust** See Section 3.3 [QTL Clustering], page 13, the result can be formatted using **QTLClustInfo**. This program allows user to get for a given trait, a given chromosome and a given clustering model, tables in which the results are summarized. **QTLClustInfo** computes also extra statistics as mahalanobis distances between the QTL of the clustering model, the unconditional confidence intervals (UCI) of these QTL and the predicted values of the observed QTL according to the clustering model.

5.3.1 Command Line Options

Option	Usage	Type	Explanation
`-o,--output`	required	STRING	The output file
`-c,--chr`	required	STRING	The name of the chromosome.
`-t,--trait`	required	STRING	The name of the trait.
`-r,--clust`	required	STRING	The clustering result file.
`-b,--best`	required	INTEGER	The number of QTL for the best model.
`--kmin`	required	INTEGER	The minimal value to compute the UCI.
`--kmax`	required	INTEGER	The maximal value to compute the UCI.

For example, we focus on the chromosome '8' for the trait called 'FT' for which we have previously performed a QTL clustering via **QTLClust**. We want to display the result of the clustering for the best model supposed to be the model with 5 QTL. We also want to compute the UCI by averaging over the clustering models from 1 QTL to 10 QTL. Thus we use the command

```
%java org.metatqtl.main.QTLClustInfo \
>     -c 8 -t FT -r clust_res.txt -b 5 --kmin 1 --kmax 10 -o table.txt
```

The result of this command is dumped into the file '`table.txt`' which looks like this

```
##
# Meta-QTL Table
##
--
QTL     Position        Weight  Distance        CI(95%) UCI(95%)
--
1       14.64   0.06    6.21    11.72   11.72
2       75.39   0.37    1.26    6.25    8.97
3       89.68   0.42    2.64    3.79    4.24
4       114.47  0.07    5.2     11.14   11.62
5       165.25  0.09    -       13.86   13.86
--
##
# Observed QTL Table
##
--
QTL   Position   CI(95%)   Predicted   Memberships 1 --> 5
--
Pioneer_HT_1    1.91    20.46   14.64   1       0       0       0       0
Poupard_2001_DPS_10     20.85   14.31   14.64   1       0       0       0       0
Pioneer_HT_2    62.7    24.62   75.39   0       1       0       0       0
Lubberstedt_1997_HT_17  74.15   14.31   75.39   0       1       0       0       0
Bouchez_2002_SD_1       74.86   14.31   75.39   0       1       0       0       0
Poupard_2001_DPS_11     76.1    16.7    75.53   0       0.99    0.01    0       0
Moreau_1998_SD_5        67.72   28.62   75.68   0       0.98    0.02    0       0
Bohn_2000_DPS_11        79.23   14.9    75.96   0       0.96    0.04    0       0
Vladutu_1999_DPS_2      73.04   26.26   76.1    0       0.95    0.05    0       0
Rebai_1997_SD_18        59.86   63.15   78.96   0       0.75    0.25    0       0
Chardon_2005b_SD_1      70.96   73.74   81.64   0       0.58    0.41    0.01    0
Charcosset_unpub_SD_7   71.13   71.42   81.64   0       0.58    0.41    0.01    0
Chardon_2005b_DPS_1     70.96   74.75   81.78   0       0.57    0.42    0.01    0
Groh_1998_HT_6  81.08   32.61   81.96   0       0.54    0.46    0       0
Mechin_2001_SD_4        82.66   21.44   83.25   0       0.45    0.55    0       0
Rebai_1997_SD_4 83.22   27.4    83.68   0       0.42    0.58    0       0
Rebai_1997_SD_31        83.96   30.97   84.11   0       0.39    0.61    0       0
Blanc_SXflofch8 85.04   37.98   84.39   0       0.37    0.63    0       0
Chardon_2005b_DPS_2     90.64   75.22   84.49   0       0.37    0.58    0.04    0
Chardon_2005b_SD_2      90.64   47.75   86.17   0       0.28    0.7     0.02    0
Groh_1998_SD_5  87.6    29.56   86.82   0       0.2     0.8     0       0
Blanc_SDflofch8 91.05   25.99   88.97   0       0.05    0.95    0       0
Moreau_1998_SD_6        93.8    11.92   89.68   0       0       1       0       0
Vladutu_1999_DPS_1      88.39   10.15   89.68   0       0       1       0       0
Chardon_2005a_SD_1      91.39   14.31   89.68   0       0       1       0       0
Chardon_2005a_DPS_1     91.39   7.13    89.68   0       0       1       0       0
Barierre_2005_SD_3      87.52   9.53    89.68   0       0       1       0       0
Vladutu_1999_HT_1       88.39   10.15   89.68   0       0       1       0       0
Blanc_SFflofch8 103.31  31.01   95.64   0       0.01    0.79    0.21    0
Pioneer_HT_3    112.74  12.31   114.47  0       0       0       1       0
Ribaut_1996_SD_4        126.15  28.46   114.47  0       0       0       1       0
Pioneer_HT_4    169.52  19.01   165.25  0       0       0       0       1
Cardinal_2001_DPS_7     167.11  45.98   165.25  0       0       0       0       1
Lubberstedt_1997_HT_16  156.52  26.22   165.25  0       0       0       0       1
--
```

5.4 QTLModel

Once the best clustering models have been identified, **QTLModel** can be used to format the clustering results into a single XML file where both the markers and the best QTL positions are given. This files can then be re-formatted using **Xml2A**.

5.4.1 Command Line Options

Option	Usage	Type	Explanation
`-m,--map`	required	STRING	The XML map name.

`-r,--clust`	required	STRING	The clustering result file.
`-b,--best`	required	STRING	The best clustering file.
`-o,--outfile`	required	STRING	The output file.

5.5 References

- Lander, E. S. and Green, P. and Abrahamson, J. and Barlow, A. and Daly M.J., 1987, *MapMaker: an integrative computer package for constructing genetic linkage maps of experimental and natural populations*, Genomics 1: 174-181. The software is available at `http://www.broad.mit.edu/ftp/distribution/software/`.
- Voorrips, R.E., 2002. *MapChart: Software for the graphical presentation of linkage maps and QTLs*, The Journal of Heredity 93 (1): 77-78. The software is available at `http://www.biometris.nl/uk/Software/MapChart/`

6 Tutorial

6.1 Tutorial Introduction

The input files of the tutorial can be downloaded from the **MetaQTL** home page. In the following sections we assume that you have correctly installed the **MetaQTL** jar archive and that you unzip the tutorial archive into the directory '`data`' into the current directory. Thus, in the '`data`' directory there are the following files:

- experiments.csv : the mapping experiment table
- trait_ontology.csv : the trait ontology table
- marker_dictionary.csv : the marker dictionary table
- genetic-map : a folder which contains the raw genetic maps of the mapping experiments
- qtl-map : a folder which contains the result of the QTL detection for each mapping experiment
- ref-map : a folder which contains the reference map Genetic_2005 from MaizeGDB

6.2 Step 1 - Creating the XML Database

To create the XML database from the raw database stored into the folder '`data`', use the command `MetaDB` as follows:

```
$java org.metaqtl.main.MetaDB -e data/experiments.csv -m data/genetic-map \
                              -q data/qtl-map -t data/trait_ontology.csv \
      -d data/marker_dictionary.csv -o xml
```

Note that the command is run into the current directoy (not in the directoy '`data`') and that the ouput XML database is created into the directoy '`xml`' (it will be created by the program). Now, if we have a look to '`xml`' we can see 19 xml files, 18 summarizing the information of the 18 input mapping experiments and one file '`ontology.xml`' which gives the XML representation of the input trait ontology. Then, we will no longer use the file in the folder '`data`': only the XML files in '`xml`' must be used to carry out further studies with **MetaQTL**.

6.3 Step 2 - Building the consensus map

For clarity, and to avoid to put all the output files into the current directory, create a directory called '`meta-map`' into which files related to the consensus map construction will be stored.

6.3.1 Checking chromosome connection and marker order consistency

Before creating the consensus map using the program `ConsMap`, it is important to check if the input maps are consistent between them. Consistency means here that: i) it must exist a path of common markers which connects the same chromosomes in the different input maps, ii) the marker order must be globally conserved between input maps which share more than 1 common marker.

To get a first diagnostic of the nature of the marker connection bewteen input maps run the command `InfoMap` as follows:

```
$java org.metaqtl.main.InfoMap -m xml -o meta-map/infomap -t 2
```

Note that we set the option `-t` to 2, in order to discard the markers which are not common between maps. Then go into the directory '`meta-map`'. There are now two files:

- infomap_cmp.txt: this file gives chromosome by chromosome the nature of the marker connection between input maps
- infomap_mrk.txt: this files gives chromosome by chromosome the occurence of the common markers between input maps.

Let's focus on the chromosome 1. If we look at the file '`infomap_cmp.txt`' we get:

```
>CR 1 Connected=true
#
# Table of the chromosomes
#
1       Barierre_2005   3       71.4333333333333
2       Blanc_2003      11      20.563636363636366
3       Bohn_1996       10      16.9
4       Bohn_2000       12      25.166666666666597
5       Cardinal_2001   12      18.908333333333342
6       Charcosset_unpub        11      23.181818181818194
7       Groh_1998       13      17.415384615384614
8       Lubberstedt_1997        6       44.666666666666764
9       Mechin_2001     20      12.900000000000006
10      Moreau_1998     8       24.74999999999999
11      Pioneer_1995    9       30.455555555555556
12      Poupard_2001    26      10.600000000000025
13      Rebai_1997      18      15.833333333333364
14      Ribaut_1996     11      24.18181818181821
```

The fact that the attribute `Connected` is equal to `true` means that it exists a path between common markers which connects all the chromosome together (in other words, a consensus chromosome one can be built using `ConsMap`). The follows the average marker interval distance in the 14 input mapping experiments - not 18 because for 4 of them there is no information about chromosome 1.

Now, have a look to the table called "Table of the number of common marker sequences" below:

#
Table of the number of common sequences
#

Chrom	Index	1	2	3	4	5	6	7	8	9	10	11	12	13	14
Barierre_2005	1		1.0										1.0		
Blanc_2003	2					1.0							3.0		
Bohn_1996	3				1.0	1.0	1.0	1.0	1.0	1.0	1.0	1.0	1.0	2.0	1.0
Bohn_2000	4					1.0	1.0	1.0	1.0	1.0	1.0	1.0	1.0	3.0	1.0
Cardinal_2001	5						1.0	1.0	1.0	1.0	1.0	1.0	1.0	1.0	1.0
Charcosset_unpub	6							1.0	1.0	1.0	1.0	1.0	1.0	2.0	1.0
Groh_1998	7								1.0	1.0	1.0	1.0	1.0	4.0	1.0
Lubberstedt_1997	8									1.0	1.0	1.0	1.0	2.0	
Mechin_2001	9										1.0	1.0	5.0	2.0	1.0
Moreau_1998	10											1.0	1.0	2.0	1.0
Pioneer_1995	11												1.0	1.0	1.0
Poupard_2001	12													2.0	1.0
Rebai_1997	13														2.0
Ribaut_1996	14														

The red cells indicate pairwise map comparisons for which we observed more than one common marker sequence. This means that there might be marker inversions between these maps. To see which markers are involved in these inversions, we propose to use the program `MMapView`.

Let's start with the pairwise comparison between the genetic maps of experiments Poupard_2001 and Mechin_2001. First create the directory 'images' into 'meta-map' and run the following command line:

```
$java org.metaqtl.main.MMapView -r xml/Poupard_2001.xml -m xml/Mechin_2001.xml \
                                -c 1 --mrkt 2 -o meta-map/images/PoupardVSMechin
```

This creates two files: 'PoupardVSMechin_1.jpeg' and 'PoupardVSMechin_1.par'. The first one is the image which represents the comparison between the chromosomes 1 of the two experiments and the second one the plot parameter used to generate the image. By default, the common marker sequences are not drawn so that we must modify the plot parameter file by setting the parameter WITH_COMMON_MARKER to true.

```
WITH_COMMON_MARKER=true
```

Other parameters can be aslo modified to obtain a better display of the chromosomes. For example:

```
CHROM_DISTANCE_SCALE=3.0 #default is 5
COMMON_STROKE_WIDTH=1.0  #default is 5  (the stroke used to show the common sequences)
LAYER_VSPACE=50.0        #default is 50 (this increases the space between the chromoso
```

Now move the plot parameter file to 'meta-map/images/comparison.par' (this because we will use the same parameter file for all the comparisons to do) and run the command:

```
$java org.metaqtl.main.MMapView -r xml/Poupard_2001.xml -m xml/Mechin_2001.xml \
                                -c 1 --mrkt 2 -o meta-map/images/PoupardVSMechin \
-p meta-map/images/comparison.par
```

Now, let's have a look to the file 'meta-map/images/PoupardVSMechin.jpeg' (use your file/web browser or a standard image viewer)

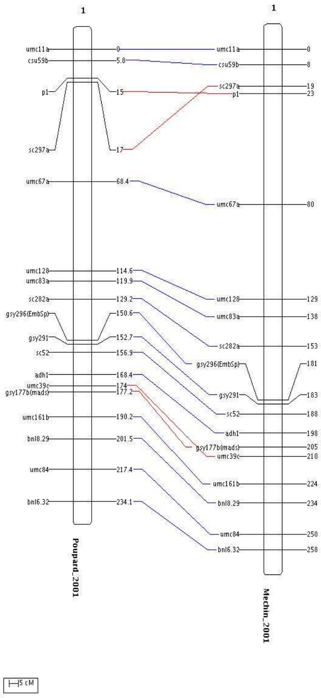

Indeed, there are 3 common sequences in the same order (the blue ones) and two common marker sequences in reverse order (the red ones). We can see that these inversions involve very close markers in both chromosome maps. Besides, if we have a look to the file '`infomap_mrk.txt`' we can see that:

>CR 1 NM=49															
#															
# Table of the chromosomes															
#															
1	Barierre_2005														
2	Blanc_2003														
3	Bohn_1996														
4	Bohn_2000														
5	Cardinal_2001														
6	Charcosset_unpub														
7	Groh_1998														
8	Lubberstedt_1997														
9	Mechin_2001														
10	Moreau_1998														
11	Pioneer_1995														
12	Poupard_2001														
13	Rebai_1997														
14	Ribaut_1996														
#															
# Table of markers															
#															
Marker	Occurence	1	2	3	4	5	6	7	8	9	10	11	12	13	14
...	...														
csu92a	3			x				x						x	
gsy177b(mads)	2									x			x		
gsy291	2									x			x		
gsy296(EmbSp)	2									x			x		
npi97a	2							x							x
p1	2									x			x		
sc282a	2									x			x		
sc297a	2									x			x		
sc52	2									x			x		
...	...														
umc33a	3			x	x										x
umc39c	3						x			x			x		
umc49c	3				x			x						x	
...	...														

only the marker `umc39c` is observed in another mapping experiment, `Charcosset_unpub`. So removing a marker per inversion will not degrade the consensus map construction for the chromosome 1.

Then, we create the file '`mrkrm.txt`' in the folder `meta-map` in order to record the two markers to remove

```
Mechin_2001 1 p1
Mechin_2001 1 gsy177b(mads)
Poupard_2001 1 p1
Poupard_2001 1 gsy177b(mads)
```

If we repeat this operation for the other pairwise comparison showing a number of commom marker sequences greater than 1, we can establish a full list of dubious markers to remove (note that in some cases you can update the name of the marker instead of removing it using the option `--mrkup` in `MetaDB`):

```
Mechin_2001     1       p1
Mechin_2001     1       gsy177b(mads)
Poupard_2001    1       p1
Poupard_2001    1       gsy177b(mads)
Blanc_2003      1       umc1278
Poupard_2001    1       umc1278
Rebai_1997      1       umc11a
Rebai_1997      1       umc58
Rebai_1997      1       umc49c
Ribaut_1996     1       npi97a
Groh_1998       1       npi97a
```

Then, we have to run back the command `MetaDB` with the option `--mrkrm` to remove these markers from the XML database:

```
$java org.metaqtl.main.MetaDB -e data/experiments.csv -m data/genetic-map \
                              -q data/qtl-map -t data/trait_ontology.csv \
      -d data/marker_dictionary.csv -o xml \
      --mrkm meta-map/mrkrm.txt
```

You can run back the command `InfoMap` to see that now there are no longer marker inversions between the chromosome 1 of the different input maps. Of course the operations described in this section must be repeated for all the chromosomes.

6.3.2 Building the consensus map

When all the marker inversions have been resolved, we can build the consensus map using the command `ConsMap`.

```
$java org.metaqtl.main.ConsMap -m xml -o meta-map/consensus
```

This creates two files into the directory ‘`meta-map`’:

- ‘`consensus_map.xml`’: the XML representation of the consensus map.
- ‘`consensus_fit.txt`’: the standardized residuals between the marker intervals in the input maps and the corresponding ones in the consensus map.

Then, if we want to display the consensus map in tabulated text format, we can use the command `Xml2A` as follows:

```
$java org.metaqtl.main.Xml2A -x meta-map/consensus_map.xml -t map -f tab \
                             -o meta-map/consensus_map.txt
```

Open the file ‘`consensus_map.txt`’. For the chromosome 1 you should have something like that:

```
map group locus type position meta.occurence
1 ph1056 M 0 1
1 bnlg1124 M 4.71 1
1 bnl5.62a M 6.7 6
1 umc164 M 9.98 2
1 umc94a M 11.7 4
1 umc1177 M 12.51 1
...  ...  ...  ...  ...
1 umc86a M 248.82 1
1 phi064 M 250.13 1
1 phi22756 M 253.6 1
1 bnl6.32 M 254.1 9
1 umc1797 M 268.5 1
```

The first column `map` is empty since by default the consensus map has no name. To add a name to the consensus map you must edit by hand the file `consensus_map.xml` and to put the suited name of the consensus map into double quoted after the attribute `name` in the tag `genome`:

```
<genome-map name="my_consensus_map">
```

The last column `meta.occurence` gives the number of times the marker have been seen into the input mapping experiments on the same chromosome (it should be the same than in the file ‘`infomap_mrk.txt`’ previously generated).

Let’s plot the histogram of these values.

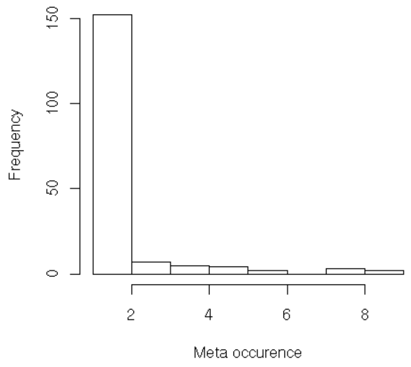

We can see that we have an excess of singleton markers, i.e. of markers which have observed only on a single mapping experiments. So the consensus map must be considered with care. In order to see how this excess of singleton markers can affect the construction of the consensus map, it could be interesting to compare the consensus map with a reference map.

For example, we downloaded from the **MaizeGDB** web site the reference map called **Genetic 2005**. This raw text file of this reference map is in the directory '`data/ref-map`' and it is called '`genetic_2005.txt`'. So, before making the comparison with our consensus map, we have to convert this file into a XML representation. To do so, use the command `A2Xml` as follows:

```
$java org.metaqtl.main.A2Xml -i data/ref-map/genetic_2005.txt -t map -f tab
                             -o ref-map/genetic_2005.xml
```

Remark: DO NOT CREATE the XML reference map into the directory '`xml`'. If not, the reference map will be used in the subsequent analyses as a mapping experiment.

To compare our consensus map with the reference map we then use the command `MMapView` as follows:

```
$java org.metaqtl.main.MMapView -m meta-map/consensus_map.xml
                                -r ref-map/genetic_2005.xml \
                                -c 1 --mrkt 2 -o meta-map/images/RefVsCons \
-p meta-map/images/comparison.par
```

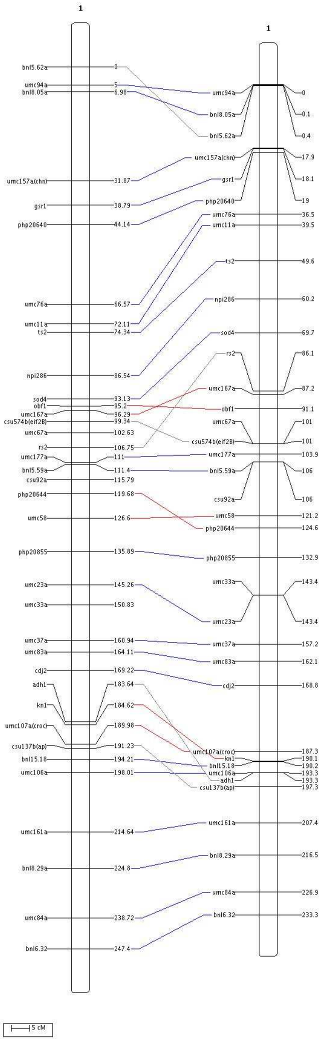

We can see that there are 8 inversions and that some of these inversions are not common sequences (in gray). Now, let's have a look to the occurence of the 11 markers involved in these inversions (into the file '`consensus_map.txt`'):

```
...  ...  ...  ...  ...
1 bnl5.62a M 6.7 6
...  ...  ...  ...  ...
1 obf1 M 101.9 1
1 umc167a M 102.99 1
...  ...  ...  ...  ...
1 csu574b(eif2B) M 106.04 1
...  ...  ...  ...  ...
1 rs2 M 113.45 1
...  ...  ...  ...  ...
1 php20644 M 126.38 1
...  ...  ...  ...  ...
1 umc58 M 133.3 5
```

```
...   ...   ...   ...   ...
1 adh1 M 190.34 3
1 kn1 M 191.32 1
...   ...   ...   ...   ...
1 umc107a(croc) M 196.68 3
1 csu137b(ap) M 197.93 1
...   ...   ...   ...   ...
```

Now, let's have a look to the way these markers are distributed among the input mapping experiments using the file '`infomap_mrk.txt`' (note that if you have kept the option `-t` set to 2 as in the previous example of the use of `InfoMap` you need to run again the command by discarding this option in order to get the information for the singleton markers).

Marker	Occurence	1	2	3	4	5	6	7	8	9	10	11	12	13	14
umc167a	1			x											
obf1	1						x								
kn1	1						x								
rs2	1		x												
csu137b(ap)	1						x								
php20644	1											x			
csu574b(eif2B)	1											x			
adh1	3									x			x	x	
umc107a(croc)	3				x									x	x
umc58	5			x	x		x		x		x				
bnl5.62a	6				x	x	x					x		x	x

Then, let's remove the 7 singleton markers (add the following list to the file '`mrkrm.txt`' previously edited):

```
Bohn_1996       1       umc167a
Blanc_2003      1       rs2
Charcosset_unpub        1       obf1
Charcosset_unpub        1       kn1
Charcosset_unpub        1       csu137b(ap)
Pioneer_1995    1       php20644
Pioneer_1995    1       csu574b(eif2B)
```

Finally, recreate the XML database and rebuild the consensus map

```
$java org.metaqtl.main.MetaDB -e data/experiments.csv -m data/genetic-map \
                              -q data/qtl-map -t data/trait_ontology.csv \
      -d data/marker_dictionary.csv -o xml \
      --mrkm meta-map/mrkrm.txt
$java org.metaqtl.main.ConsMap -m xml -o meta-map/consensus
```

Now, if we want to visualize if there are some standarized residuals (the ones stored in the file '`consensus_fit.txt`') which are significatively outside the expected distribution, we can use the command `MMapView` by setting the options `--htest` and `--hth`. The last option `--hth` allows the user to define the level of the test. For example, if we want to get the marker intervals which standarized residuals are significatively outside the expected distribution at a level of 5\%, we will use the following command:

```
$java org.metaqtl.main.MMapView -r meta-map/consensus_map.xml -m xml -c 1 \
                                -o meta-map/images/ConsensusVSAll \
-p meta-map/images/consensus.par \
--hth 9.5E-2 --htest
```

Note that the value of the option `--hth` must be equal to one minus the level of the test. Normaly, you will obtain something like that:

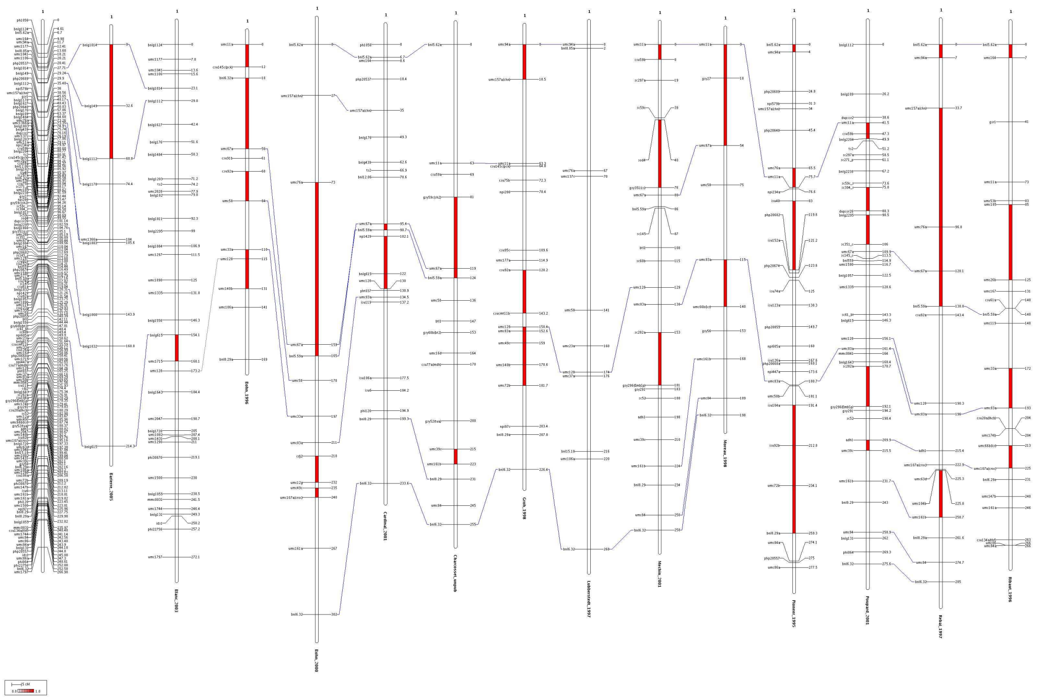

6.4 Step 3 - Projection of the QTL

Once the consensus map have been established, we can project all the observed QTL from the initial mapping experiments onto this consensus map. This is a compulsory step to carry out the meta-analysis.

To do so, use the program `QTLProj` as follows:

```
$java org.metaqtl.main.QTLProj -m meta-map/consensus_map.xml \
                               -q xml -o meta-qtl/consensus --verbose
[ WARNING ] : Unable to project QTL Rebai_1997_SD_14 on chromosome 2
[ WARNING ] : Unable to project QTL Rebai_1997_SD_27 on chromosome 2
[ WARNING ] : Unable to project QTL Rebai_1997_SD_21 on chromosome 2
[ WARNING ] : Unable to project QTL Rebai_1997_SD_7 on chromosome 2
```

Note that setting the option `--verbose` displays the warnings. In this case, it seems that there is a problem with the mapping experiment `Rebai_1997` for the chromosome 2. Since here we focus on chromosome 1, we will ignore these warnings for the rest of the tutorial.

This command generates two files into the directory '`meta-qtl`':

- '`consensus_map.xml`': the consensus map plus the projected QTL.
- '`consensus_qtl.xml`': the consensus map with only the projected QTL.

Now, if we want to see how the QTL have been projected and are distributed along the chromosome 1, we can use the command `MapView` as follows:

```
java org.metaqtl.main.MapView -m meta-qtl/consensus_map.xml -c 1 \
                              -o meta-qtl/images/consensus \
      -p meta-qtl/images/consensus.par -q
```

This should give the following picture:

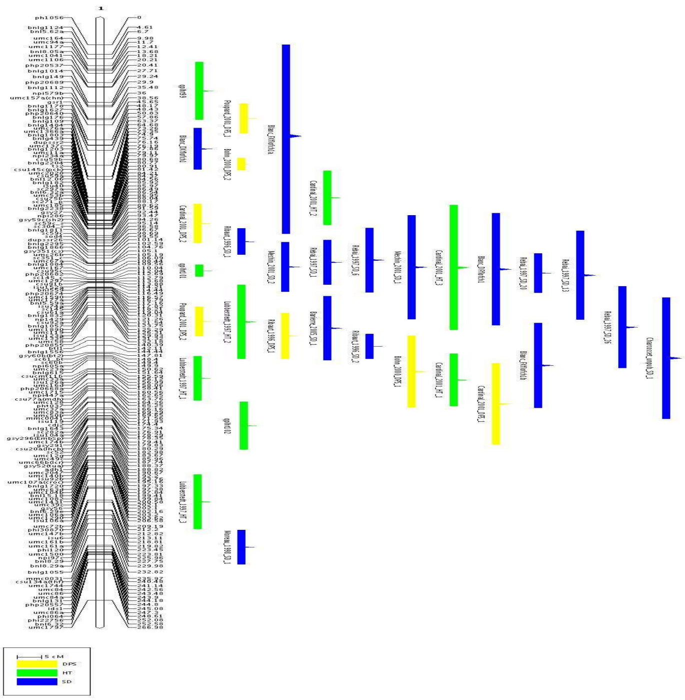

Now, if we want to see the properties of the QTL projection for each QTL we can export the file '`consensus_qtl.xml`' into a flat text file using the command `Xml2A`:

```
java org.metaqtl.main.Xml2A -x meta-qtl/consensus_qtl.xml\
                            -o meta-qtl/consensus_qtl.txt \
    -t map -f tab
```

Thus we obtain the following list for the chromosome 1:

group	locus	type	position	qtl.trait.name	qtl.ci.from	qtl.ci.to	qtl.proj.swapFlanking	qtl.proj.shareFlanking	qtl.proj.mapScale	qtl.proj.intScale
1	qplht99	Q	22.2	HT			false	true	0,92	0,87
1	Poupard_2001_DPS_1	Q	35.48	DPS	35,48	47,48	false	true	0,78	1,06
1	Blanc_FXflofch1a	Q	43.91	SD	5,91	99,91	false	true	1,02	1,03
1	Blanc_DXflofch1	Q	50.07	SD	40,07	60,07	false	true	1,03	1,02
1	Bohn_2000_DPS_2	Q	57.42	DPS			false	true	0,53	0,75
1	Cardinal_2001_HT_2	Q	73.59	HT			false	true	1,21	1,34
1	Cardinal_2001_DPS_2	Q	85.91	DPS			false	true	1,01	0,96
1	Ribaut_1996_SD_1	Q	94.61	SD			false	true	0,6	0,6
1	Rebai_1997_SD_6	Q	103.51	SD	87,51	113,51	false	true	1,02	1,04
1	Rebai_1997_SD_1	Q	104.55	SD	94,55	112,55	false	true	1,03	1,04
1	Mechin_2001_SD_2	Q	106.77	SD	90,77	110,77	false	true	0,68	0,84
1	Mechin_2001_SD_1	Q	106.94	SD	86,94	128,94	false	true	0,51	0,39
1	Cardinal_2001_HT_3	Q	107.09	HT			false	true	1,34	0,96
1	Blanc_DFflofch1	Q	107.88	SD	68,88	122,88	false	true	0,88	0,88
1	qplht101	Q	108.6	HT			false	true	0,74	0,73
1	Rebai_1997_SD_20	Q	109.73	SD	103,73	119,73	false	true	1,01	1,04
1	Rebai_1997_SD_13	Q	110.77	SD	104,77	140,77	false	true	0,98	1,04
1	Poupard_2001_DPS_2	Q	132.94	DPS	126,94	138,94	false	true	0,9	1,16
1	Lubberstedt_1997_HT_2	Q	133.15	HT	115,15	145,15	false	true	0,83	0,97
1	Rebai_1997_SD_26	Q	135.75	SD	117,75	150,75	false	true	0,86	0,91
1	Barierre_2005_SD_1	Q	136.24	SD	122,24	148,24	false	true	0,62	0,59
1	Ribaut_1996_DPS_1	Q	139.97	DPS			false	true	1,08	1,24
1	Ribaut_1996_SD_2	Q	144.94	SD			false	true	0,96	1,24
1	Charcosset_unpub_SD_1	Q	150.6	SD			false	true	0,92	0,93
1	Blanc_FXflofch1b	Q	154.13	SD	130,13	171,13	false	true	0,79	0,64
1	Bohn_2000_DPS_1	Q	157.06	DPS			false	true	1,08	0,94
1	Lubberstedt_1997_HT_1	Q	160.36	HT	150,36	168,36	false	true	0,9	0,97
1	Cardinal_2001_HT_1	Q	161.11	HT			false	true	1,32	1,58
1	Cardinal_2001_DPS_1	Q	172.62	DPS			false	true	1,03	0,86
1	qplht102	Q	183.06	HT			false	true	0,76	0,72
1	Lubberstedt_1997_HT_3	Q	219.66	HT	207,66	229,66	false	true	1	1,03
1	Moreau_1998_SD_1	Q	241.43	SD	233,43	247,43	false	true	1,12	1,13

The attributes `qtl.ci.from` and `qtl.ci.to` gives the new confidence interval of the QTL when a confidence interval exists in the intial mapping experiment. This new confidence interval is computed according to the projection scale given by the homothetic ratio gvien by the attribute `qtl.proj.mapScale`, while the position of the QTL is computed relatively to the flanking markers using the homothetic ratio given by the attribute `qtl.proj.intScale`. The boolean attributes `qtl.proj.shareFlanking` and `qtl.proj.swapFlanking` indicate for the former that the QTL flanking markers are observed into both the intial and the consensus map, and the later will be `true` if these markers are observed in a reverse order between the two maps.

Note that for half of the QTL in chromosome 1 there is no information about the CI. Nevertheless, if you look at the file '`consensus_qtl.txt`' you will see another field called `qtl.rsquare` which gives the percentage of variance explained by the QTL (this field was not included in the above table) and from which a theoretical confidence interval could be computed according to the mapping population properties (size and type of cross: these two parameters are given by the attributes `qtl.cross.size` and `qtl.cross.type`).

6.5 Step 4 - QTL Clustering

6.5.1 QTL Clustering

Now, we are ready to start the QTL meta-analysis itself using the program `QTLClust`.

```
$java org.metaqtl.main.QTLClust -q meta-qtl/consensus_map.xml -t xml/ontology.xml \
                                -k 10 -c 1 -o meta-qtl/meta-analysis_1 --verbose
```

We use here the trait ontology '`xml/ontology.xml`' so that all the observed traits are clusterized into a single meta-trait called "FT" (for Flowering Time): this means that all the observed

QTL are taken into account during the meta-analysis for the meta-trait FT. Note that by setting the option `-k` to 10 we avoid to explore models with more than 10 meta-QTL on the chromosome.

This command generates three files into the directory ‘`meta-qtl`’:

- ‘`meta-analysis_1_res.txt`’: this file contains the details of all the QTL clustering results from k=1 to 10.
- ‘`meta-analysis_1_crit.txt`’: this file gives for each model (k=1 to 10) the loglikelihood and the corresponding model choice criteria.
- ‘`meta-analysis_1_model.txt`’: this file gives for each kind of model choice criterion the best model (i.e the model which generally minimizes the criterion value).

So, let’s have a look to the file ‘`meta-analysis_1_model.txt`’:

```
Criterion       Chromosome      Trait   Model
AIC     1       FT      7
Criterion       Chromosome      Trait   Model
AICc    1       FT      7
Criterion       Chromosome      Trait   Model
AIC3    1       FT      7
Criterion       Chromosome      Trait   Model
BIC     1       FT      7
Criterion       Chromosome      Trait   Model
AWE     1       FT      7
```

We can see that for each model choice criterion (AIC,AICc,AIC3,BIC,AWE) the best model is the model with 7 QTL clusters, i.e 7 meta-QTL. To see how the observed QTL are distributed into these 7 QTL clusters we can use the program `MQTLView`. In order to run `MQTLView` we have to create a file which tells to the program which model it has to represent. To do so, simply open the file `meta-analysis_1_model.txt`, keep only one line per chromosome, remove the column called `Criterion`, and save it as ‘`meta-analysis_1_best.txt`’. Here this file must look like that

```
Chromosome      Trait   Model
1       FT      7
```

Then, we are ready to use `MQTLView`:

```
$java org.metaqtl.main.MQTLView -m meta-qtl/consensus_map.xml -c 1 \
                                -r meta-qtl/meta-analysis_1_res.txt \
-b meta-qtl/meta-analysis_1_best.txt \
-p meta-qtl/images/consensus.par \
-o meta-qtl/images/meta-analysis_1
```

This should give the following picture:

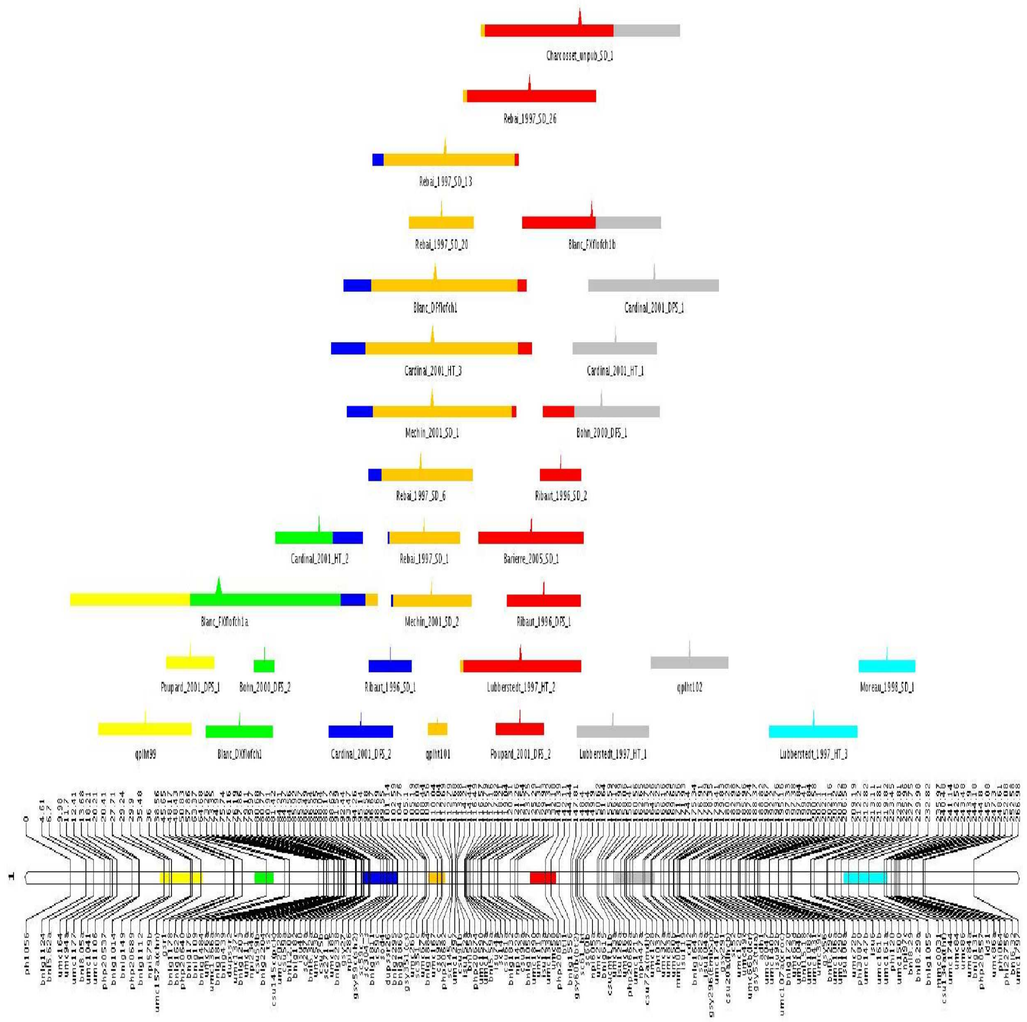

Finally, we can extract for the best model with 7 meta-QTL some useful statistics such like the confidence intervals of the meta-QTL and the *a posterori* membership probabilities of each observed QTL together with their predicted positions. To do so, use the command `QTLClustInfo` as follows:

```
$java org.metaqtl.main.QTLClustInfo -r meta-qtl/meta-analysis_1_res.txt -c 1 \
                                    -o meta-qtl/meta-analysis_1_info.txt \
   -b 7 --kmin 1 --kmax 10 -t FT
```

Let's have a look to the file '`meta-analysis_1_info.txt`':

```
##
# Meta-QTL Table
##
--
QTL     Position        Weight  Distance        CI(95%) UCI(95%)
--
```

```
1       32.82   0.07    2.21    12.65   12.65
2       57.31   0.1     3.47    5.68    22.48
3       91.52   0.1     1.56    10.07   15.95
4       108.29  0.26    2.74    5.2     5.13
5       139.76  0.24    2.43    7.68    16.89
6       166.86  0.17    7.06    11.75   16.79
7       235.16  0.06    -       12.89   12.89
--
##
# Observed QTL Table
##
--
QTL   Position   CI(95%)   Predicted   Memberships 1 --> 7
--
qplht99 22.2    27.75   32.82   1       0       0       0       0       0       0
Poupard_2001_DPS_1      35.48   14.31   32.82   1       0       0       0       0
Blanc_FXflofch1a        43.91   94      52.53   0.39    0.49    0.08    0.04    0
Blanc_DXflofch1 50.07   19.99   57.07   0.01    0.99    0       0       0       0
Bohn_2000_DPS_2 57.42   6.04    57.31   0       1       0       0       0       0
Cardinal_2001_HT_2      73.59   26.11   69.28   0       0.65    0.35    0       0
Ribaut_1996_SD_1        94.61   12.78   91.52   0       0       1       0       0
Cardinal_2001_DPS_2     85.91   18.97   91.52   0       0       1       0       0
Rebai_1997_SD_6 103.51  30.97   106.11  0       0       0.13    0.87    0       0
Cardinal_2001_HT_3      107.09  60.09   106.56  0       0       0.17    0.75    0.07
Mechin_2001_SD_1        106.94  50.06   106.72  0       0       0.15    0.82    0.03
Blanc_DFflofch1 107.88  54.02   107.35  0       0       0.15    0.8     0.05    0
Rebai_1997_SD_1 104.55  21.44   107.79  0       0       0.03    0.97    0       0
Mechin_2001_SD_2        106.77  23.83   107.95  0       0       0.02    0.98    0
Rebai_1997_SD_20        109.73  19.05   108.29  0       0       0       1       0
qplht101        108.6   5.76    108.29  0       0       0       1       0       0
Rebai_1997_SD_13        110.77  42.88   108.98  0       0       0.08    0.9     0.03
Lubberstedt_1997_HT_2   133.15  35.75   138.82  0       0       0       0.03    0.97
Barierre_2005_SD_1      136.24  30.97   139.76  0       0       0       0       1
Poupard_2001_DPS_2      132.94  14.31   139.76  0       0       0       0       1
Ribaut_1996_SD_2        144.94  12.15   139.76  0       0       0       0       1
Ribaut_1996_DPS_1       139.97  21.99   139.76  0       0       0       0       1
Rebai_1997_SD_26        135.75  39.32   140.48  0       0       0       0.03    0.97
Charcosset_unpub_SD_1   150.6   58.33   149.74  0       0       0       0.02    0.65
Blanc_FXflofch1b        154.13  41      152.5   0       0       0       0       0.53
Bohn_2000_DPS_1 157.06  34.69   159.54  0       0       0       0       0.27    0.73
Cardinal_2001_DPS_1     172.62  39.16   166.59  0       0       0       0       0.01
Cardinal_2001_HT_1      161.11  25.24   166.59  0       0       0       0       0.01
qplht102        183.06  23.01   166.86  0       0       0       0       0       1
Lubberstedt_1997_HT_1   160.36  21.44   166.86  0       0       0       0       0
Lubberstedt_1997_HT_3   219.66  26.22   235.16  0       0       0       0       0
Moreau_1998_SD_1        241.43  16.7    235.16  0       0       0       0       0
--
```

6.5.2 QTL Tree

Another techniques to group the observed QTL into clusters is to use standard agglomerative clustering technics, although this method does not make it possible to infer the best number of

clusters. Nevertheless, it can help to visualize how the observed QTL are distributed along the chromosome.

Let's run the program `QTLTree` as follows:

```
$java org.metaqtl.main.QTLTree -q meta-qtl/consensus_map.xml \
                               -o meta-qtl/meta-tree.txt \
       -t xml/ontology.xml --verbose
```

This generates one file, '`meta-tree.txt`' into the directory '`meta-qtl`'. This file contains for each chromosome a summary of the observed QTL positions and then the clustering tree computed by the algorithm in Newick format. For example, for the chromosome 1 this gives something like that:

```
CR 1
TR FT 32
QT 0 Bohn_2000_DPS_2 57.42 1.54
QT 1 Bohn_2000_DPS_1 157.06 8.85
...
QT 30 Rebai_1997_SD_1 104.55 5.47
QT 31 Mechin_2001_SD_1 106.94 12.77
HC (((((0:0.37,19:0.37):2.37,29:2.37):8.51,8:8.51):51.78,(6:14.63,10:14.63):51.78):104[illegible]
.02):2.68,26:2.68):48.32,(((2:0,11:0):0.85,(20:0.03,27:0.03):0.85):15.12,(5:2.87,(21:0[illegible]
,(4:10.41,9:10.41):106):252.78,((3:8.11,22:8.11):22.26,(((((12:0,18:0):0.04,25:0.04):0[illegible]
0):0.01):0.19):0.9,(17:0.12,30:0.12):0.9):22.26):252.78):646.19,(14:48.63,23:48.63):64[illegible]
--
```

At first sight, it is not really easy to imagine from this file the tree that connects the QTL. To display the tree, we can use the command `MQTLView`. A good point is that we can visualize together the result of the previous clustering and the obtained tree.

```
$java org.metaqtl.main.MQTLView -m meta-qtl/consensus_map.xml -c 1 \
                                 -r meta-qtl/meta-analysis_1_res.txt \
-b meta-qtl/meta-analysis_1_best.txt \
-p meta-qtl/images/consensus.par \
-o meta-qtl/images/meta-analysis_1
--tree meta-qtl/meta-tree.txt
```

Then you should get the following picture:

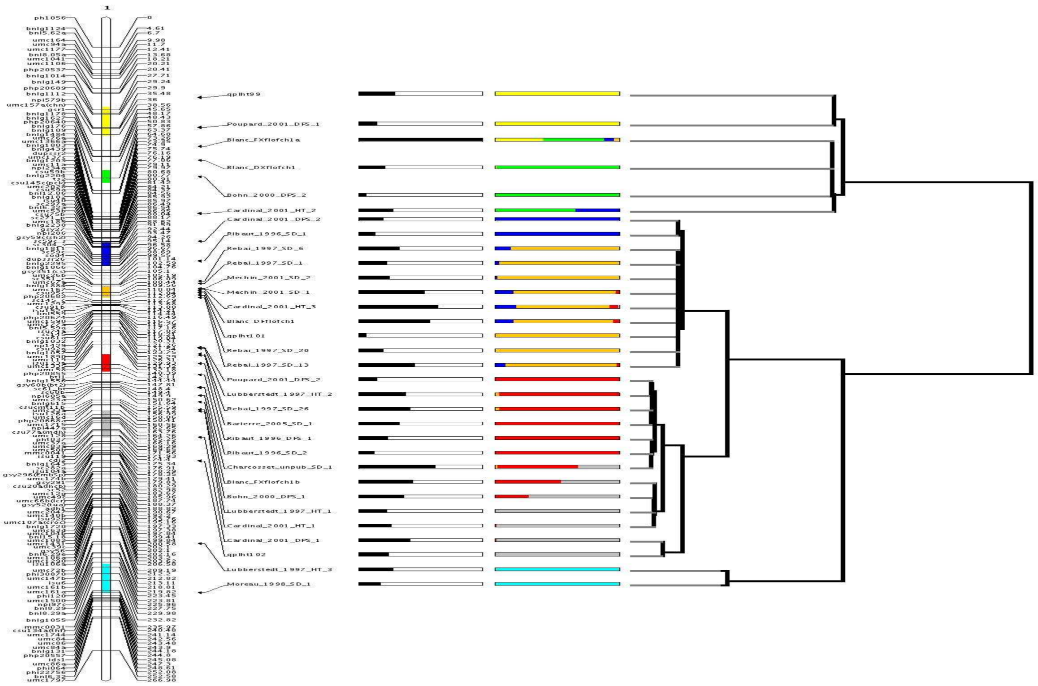

6.6 Step 4 - Post processing

6.6.1 Extracting the meta-QTL

Now, suppose we want to get the 7 meta-QTL of the chromosome 1 separetly on the consensus map. To do so, we can use the command `QTLModel` as follows:

```
$java org.metaqtl.main.QTLModel -m meta-map/consensus_map.xml \
                                -r meta-qtl/meta-analysis_1_res.txt \
-b meta-qtl/meta-analysis_1_best.txt \
-o meta-post/metamap.xml
```

Then, if we use `MapView` to display the map '`metamap.xml`' we obtain something like that:

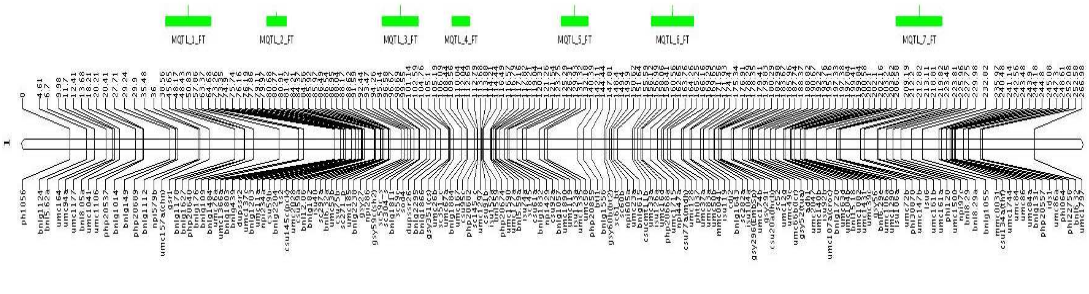

6.6.2 Projection of the meta-QTL

Finally, it could be useful to project the meta-QTL onto a reference map which provides candidate genes in order to study the congruency between these candidate genes and the meta-QTL.

Then, suppose we want to project the meta-QTL onto the reference map `genetic_2005` which we have previously used. So, we can use the program `QTLProj` as follows:

```
$java org.metaqtl.main.QTLProj -m ref-map/genetic_2005.xml \
                               -q meta-post/metamap.xml \
       -o meta-post/genetic_2005 \
       --verbose
```

Then, if we use `MMapView` to display the result of the projection we obtain something like that:

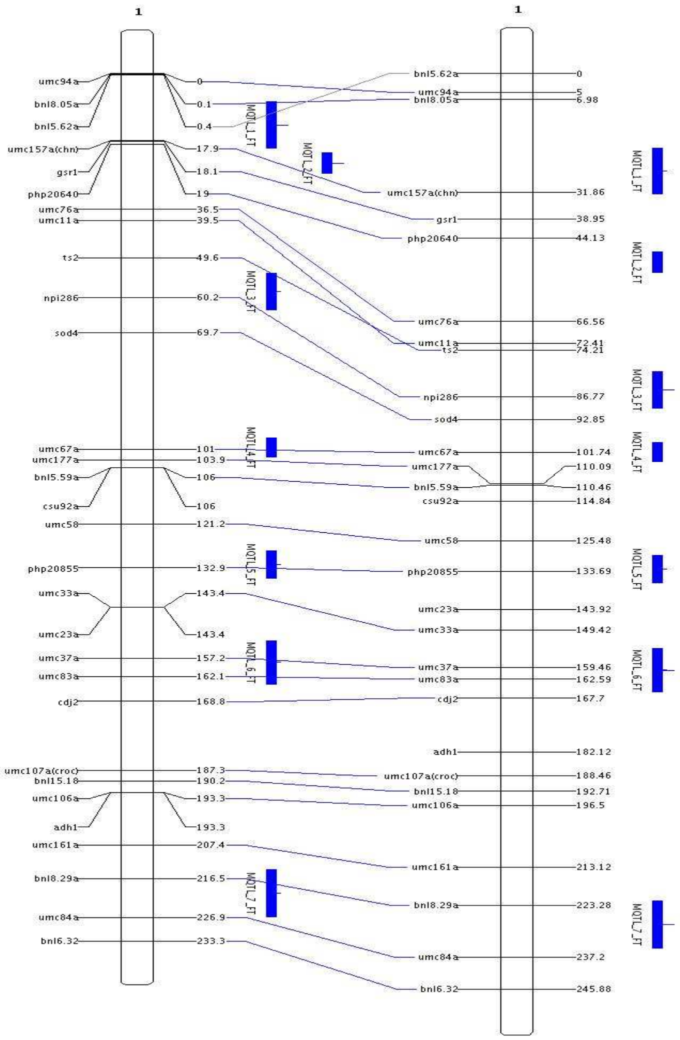

Appendix A Copying This Manual

Version 1.2, November 2002

Copyright © 2000,2001,2002 Free Software Foundation, Inc.
59 Temple Place, Suite 330, Boston, MA 02111-1307, USA

Everyone is permitted to copy and distribute verbatim copies
of this license document, but changing it is not allowed.

0. PREAMBLE

   The purpose of this License is to make a manual, textbook, or other functional and useful document *free* in the sense of freedom: to assure everyone the effective freedom to copy and redistribute it, with or without modifying it, either commercially or noncommercially. Secondarily, this License preserves for the author and publisher a way to get credit for their work, while not being considered responsible for modifications made by others.

   This License is a kind of "copyleft", which means that derivative works of the document must themselves be free in the same sense. It complements the GNU General Public License, which is a copyleft license designed for free software.

   We have designed this License in order to use it for manuals for free software, because free software needs free documentation: a free program should come with manuals providing the same freedoms that the software does. But this License is not limited to software manuals; it can be used for any textual work, regardless of subject matter or whether it is published as a printed book. We recommend this License principally for works whose purpose is instruction or reference.

1. APPLICABILITY AND DEFINITIONS

   This License applies to any manual or other work, in any medium, that contains a notice placed by the copyright holder saying it can be distributed under the terms of this License. Such a notice grants a world-wide, royalty-free license, unlimited in duration, to use that work under the conditions stated herein. The "Document", below, refers to any such manual or work. Any member of the public is a licensee, and is addressed as "you". You accept the license if you copy, modify or distribute the work in a way requiring permission under copyright law.

   A "Modified Version" of the Document means any work containing the Document or a portion of it, either copied verbatim, or with modifications and/or translated into another language.

   A "Secondary Section" is a named appendix or a front-matter section of the Document that deals exclusively with the relationship of the publishers or authors of the Document to the Document's overall subject (or to related matters) and contains nothing that could fall directly within that overall subject. (Thus, if the Document is in part a textbook of mathematics, a Secondary Section may not explain any mathematics.) The relationship could be a matter of historical connection with the subject or with related matters, or of legal, commercial, philosophical, ethical or political position regarding them.

   The "Invariant Sections" are certain Secondary Sections whose titles are designated, as being those of Invariant Sections, in the notice that says that the Document is released under this License. If a section does not fit the above definition of Secondary then it is not allowed to be designated as Invariant. The Document may contain zero Invariant Sections. If the Document does not identify any Invariant Sections then there are none.

   The "Cover Texts" are certain short passages of text that are listed, as Front-Cover Texts or Back-Cover Texts, in the notice that says that the Document is released under this License. A Front-Cover Text may be at most 5 words, and a Back-Cover Text may be at most 25 words.

A “Transparent” copy of the Document means a machine-readable copy, represented in a format whose specification is available to the general public, that is suitable for revising the document straightforwardly with generic text editors or (for images composed of pixels) generic paint programs or (for drawings) some widely available drawing editor, and that is suitable for input to text formatters or for automatic translation to a variety of formats suitable for input to text formatters. A copy made in an otherwise Transparent file format whose markup, or absence of markup, has been arranged to thwart or discourage subsequent modification by readers is not Transparent. An image format is not Transparent if used for any substantial amount of text. A copy that is not “Transparent” is called “Opaque”.

Examples of suitable formats for Transparent copies include plain ASCII without markup, Texinfo input format, LaTeX input format, SGML or XML using a publicly available DTD, and standard-conforming simple HTML, PostScript or PDF designed for human modification. Examples of transparent image formats include PNG, XCF and JPG. Opaque formats include proprietary formats that can be read and edited only by proprietary word processors, SGML or XML for which the DTD and/or processing tools are not generally available, and the machine-generated HTML, PostScript or PDF produced by some word processors for output purposes only.

The “Title Page” means, for a printed book, the title page itself, plus such following pages as are needed to hold, legibly, the material this License requires to appear in the title page. For works in formats which do not have any title page as such, “Title Page” means the text near the most prominent appearance of the work’s title, preceding the beginning of the body of the text.

A section “Entitled XYZ” means a named subunit of the Document whose title either is precisely XYZ or contains XYZ in parentheses following text that translates XYZ in another language. (Here XYZ stands for a specific section name mentioned below, such as “Acknowledgements”, “Dedications”, “Endorsements”, or “History”.) To “Preserve the Title” of such a section when you modify the Document means that it remains a section “Entitled XYZ” according to this definition.

The Document may include Warranty Disclaimers next to the notice which states that this License applies to the Document. These Warranty Disclaimers are considered to be included by reference in this License, but only as regards disclaiming warranties: any other implication that these Warranty Disclaimers may have is void and has no effect on the meaning of this License.

2. VERBATIM COPYING

You may copy and distribute the Document in any medium, either commercially or noncommercially, provided that this License, the copyright notices, and the license notice saying this License applies to the Document are reproduced in all copies, and that you add no other conditions whatsoever to those of this License. You may not use technical measures to obstruct or control the reading or further copying of the copies you make or distribute. However, you may accept compensation in exchange for copies. If you distribute a large enough number of copies you must also follow the conditions in section 3.

You may also lend copies, under the same conditions stated above, and you may publicly display copies.

3. COPYING IN QUANTITY

If you publish printed copies (or copies in media that commonly have printed covers) of the Document, numbering more than 100, and the Document’s license notice requires Cover Texts, you must enclose the copies in covers that carry, clearly and legibly, all these Cover Texts: Front-Cover Texts on the front cover, and Back-Cover Texts on the back cover. Both covers must also clearly and legibly identify you as the publisher of these copies. The front cover must present the full title with all words of the title equally prominent and visible.

You may add other material on the covers in addition. Copying with changes limited to the covers, as long as they preserve the title of the Document and satisfy these conditions, can be treated as verbatim copying in other respects.

If the required texts for either cover are too voluminous to fit legibly, you should put the first ones listed (as many as fit reasonably) on the actual cover, and continue the rest onto adjacent pages.

If you publish or distribute Opaque copies of the Document numbering more than 100, you must either include a machine-readable Transparent copy along with each Opaque copy, or state in or with each Opaque copy a computer-network location from which the general network-using public has access to download using public-standard network protocols a complete Transparent copy of the Document, free of added material. If you use the latter option, you must take reasonably prudent steps, when you begin distribution of Opaque copies in quantity, to ensure that this Transparent copy will remain thus accessible at the stated location until at least one year after the last time you distribute an Opaque copy (directly or through your agents or retailers) of that edition to the public.

It is requested, but not required, that you contact the authors of the Document well before redistributing any large number of copies, to give them a chance to provide you with an updated version of the Document.

4. MODIFICATIONS

   You may copy and distribute a Modified Version of the Document under the conditions of sections 2 and 3 above, provided that you release the Modified Version under precisely this License, with the Modified Version filling the role of the Document, thus licensing distribution and modification of the Modified Version to whoever possesses a copy of it. In addition, you must do these things in the Modified Version:

   A. Use in the Title Page (and on the covers, if any) a title distinct from that of the Document, and from those of previous versions (which should, if there were any, be listed in the History section of the Document). You may use the same title as a previous version if the original publisher of that version gives permission.
   B. List on the Title Page, as authors, one or more persons or entities responsible for authorship of the modifications in the Modified Version, together with at least five of the principal authors of the Document (all of its principal authors, if it has fewer than five), unless they release you from this requirement.
   C. State on the Title page the name of the publisher of the Modified Version, as the publisher.
   D. Preserve all the copyright notices of the Document.
   E. Add an appropriate copyright notice for your modifications adjacent to the other copyright notices.
   F. Include, immediately after the copyright notices, a license notice giving the public permission to use the Modified Version under the terms of this License, in the form shown in the Addendum below.
   G. Preserve in that license notice the full lists of Invariant Sections and required Cover Texts given in the Document's license notice.
   H. Include an unaltered copy of this License.
   I. Preserve the section Entitled "History", Preserve its Title, and add to it an item stating at least the title, year, new authors, and publisher of the Modified Version as given on the Title Page. If there is no section Entitled "History" in the Document, create one stating the title, year, authors, and publisher of the Document as given on its Title Page, then add an item describing the Modified Version as stated in the previous sentence.

J. Preserve the network location, if any, given in the Document for public access to a Transparent copy of the Document, and likewise the network locations given in the Document for previous versions it was based on. These may be placed in the "History" section. You may omit a network location for a work that was published at least four years before the Document itself, or if the original publisher of the version it refers to gives permission.
K. For any section Entitled "Acknowledgements" or "Dedications", Preserve the Title of the section, and preserve in the section all the substance and tone of each of the contributor acknowledgements and/or dedications given therein.
L. Preserve all the Invariant Sections of the Document, unaltered in their text and in their titles. Section numbers or the equivalent are not considered part of the section titles.
M. Delete any section Entitled "Endorsements". Such a section may not be included in the Modified Version.
N. Do not retitle any existing section to be Entitled "Endorsements" or to conflict in title with any Invariant Section.
O. Preserve any Warranty Disclaimers.

If the Modified Version includes new front-matter sections or appendices that qualify as Secondary Sections and contain no material copied from the Document, you may at your option designate some or all of these sections as invariant. To do this, add their titles to the list of Invariant Sections in the Modified Version's license notice. These titles must be distinct from any other section titles.

You may add a section Entitled "Endorsements", provided it contains nothing but endorsements of your Modified Version by various parties—for example, statements of peer review or that the text has been approved by an organization as the authoritative definition of a standard.

You may add a passage of up to five words as a Front-Cover Text, and a passage of up to 25 words as a Back-Cover Text, to the end of the list of Cover Texts in the Modified Version. Only one passage of Front-Cover Text and one of Back-Cover Text may be added by (or through arrangements made by) any one entity. If the Document already includes a cover text for the same cover, previously added by you or by arrangement made by the same entity you are acting on behalf of, you may not add another; but you may replace the old one, on explicit permission from the previous publisher that added the old one.

The author(s) and publisher(s) of the Document do not by this License give permission to use their names for publicity for or to assert or imply endorsement of any Modified Version.

5. COMBINING DOCUMENTS

You may combine the Document with other documents released under this License, under the terms defined in section 4 above for modified versions, provided that you include in the combination all of the Invariant Sections of all of the original documents, unmodified, and list them all as Invariant Sections of your combined work in its license notice, and that you preserve all their Warranty Disclaimers.

The combined work need only contain one copy of this License, and multiple identical Invariant Sections may be replaced with a single copy. If there are multiple Invariant Sections with the same name but different contents, make the title of each such section unique by adding at the end of it, in parentheses, the name of the original author or publisher of that section if known, or else a unique number. Make the same adjustment to the section titles in the list of Invariant Sections in the license notice of the combined work.

In the combination, you must combine any sections Entitled "History" in the various original documents, forming one section Entitled "History"; likewise combine any sections Entitled

"Acknowledgements", and any sections Entitled "Dedications". You must delete all sections Entitled "Endorsements."

6. COLLECTIONS OF DOCUMENTS

   You may make a collection consisting of the Document and other documents released under this License, and replace the individual copies of this License in the various documents with a single copy that is included in the collection, provided that you follow the rules of this License for verbatim copying of each of the documents in all other respects.

   You may extract a single document from such a collection, and distribute it individually under this License, provided you insert a copy of this License into the extracted document, and follow this License in all other respects regarding verbatim copying of that document.

7. AGGREGATION WITH INDEPENDENT WORKS

   A compilation of the Document or its derivatives with other separate and independent documents or works, in or on a volume of a storage or distribution medium, is called an "aggregate" if the copyright resulting from the compilation is not used to limit the legal rights of the compilation's users beyond what the individual works permit. When the Document is included in an aggregate, this License does not apply to the other works in the aggregate which are not themselves derivative works of the Document.

   If the Cover Text requirement of section 3 is applicable to these copies of the Document, then if the Document is less than one half of the entire aggregate, the Document's Cover Texts may be placed on covers that bracket the Document within the aggregate, or the electronic equivalent of covers if the Document is in electronic form. Otherwise they must appear on printed covers that bracket the whole aggregate.

8. TRANSLATION

   Translation is considered a kind of modification, so you may distribute translations of the Document under the terms of section 4. Replacing Invariant Sections with translations requires special permission from their copyright holders, but you may include translations of some or all Invariant Sections in addition to the original versions of these Invariant Sections. You may include a translation of this License, and all the license notices in the Document, and any Warranty Disclaimers, provided that you also include the original English version of this License and the original versions of those notices and disclaimers. In case of a disagreement between the translation and the original version of this License or a notice or disclaimer, the original version will prevail.

   If a section in the Document is Entitled "Acknowledgements", "Dedications", or "History", the requirement (section 4) to Preserve its Title (section 1) will typically require changing the actual title.

9. TERMINATION

   You may not copy, modify, sublicense, or distribute the Document except as expressly provided for under this License. Any other attempt to copy, modify, sublicense or distribute the Document is void, and will automatically terminate your rights under this License. However, parties who have received copies, or rights, from you under this License will not have their licenses terminated so long as such parties remain in full compliance.

10. FUTURE REVISIONS OF THIS LICENSE

    The Free Software Foundation may publish new, revised versions of the GNU Free Documentation License from time to time. Such new versions will be similar in spirit to the present version, but may differ in detail to address new problems or concerns. See `http://www.gnu.org/copyleft/`.

    Each version of the License is given a distinguishing version number. If the Document specifies that a particular numbered version of this License "or any later version" applies to it, you have the option of following the terms and conditions either of that specified

version or of any later version that has been published (not as a draft) by the Free Software Foundation. If the Document does not specify a version number of this License, you may choose any version ever published (not as a draft) by the Free Software Foundation.

ADDENDUM: How to use this License for your documents

To use this License in a document you have written, include a copy of the License in the document and put the following copyright and license notices just after the title page:

```
Copyright (C)  year  your name.
Permission is granted to copy, distribute and/or modify this document
under the terms of the GNU Free Documentation License, Version 1.2
or any later version published by the Free Software Foundation;
with no Invariant Sections, no Front-Cover Texts, and no Back-Cover
Texts.  A copy of the license is included in the section entitled ``GNU
Free Documentation License''.
```

If you have Invariant Sections, Front-Cover Texts and Back-Cover Texts, replace the "with...Texts." line with this:

```
with the Invariant Sections being list their titles, with
the Front-Cover Texts being list, and with the Back-Cover Texts
being list.
```

If you have Invariant Sections without Cover Texts, or some other combination of the three, merge those two alternatives to suit the situation.

If your document contains nontrivial examples of program code, we recommend releasing these examples in parallel under your choice of free software license, such as the GNU General Public License, to permit their use in free software.
