# Supplementary material for: MetaQTL: a package of new computational methods for the meta-analysis of QTL mapping experiments
Source: BMC Bioinformatics. 2007 Feb 8;8:49. doi: 10.1186/1471-2105-8-49 (PMC1808479; doi:10.1186/1471-2105-8-49)
Supplement: Additional File 6 — Summary of the QTL mapping experiments used in the application. This PDF file gives details on the mapping experiments used in our application. [file 1471-2105-8-49-S6.pdf]

QTL experiments	Parents	Type of population	Population size	Traits	Reference
Barrière	F838 × F286	RIL	242	SD	[1]
Bohn1	CML131 × CML67	F_2	215	HT	[3]
Bohn2	B73 × Mo17	F_2	226	DPS	[4]
Bouchez	F2 × MBS847	BC_3S_1	217	SD	[5]
Cardinal	B73 × B52	RIL	200	DPS,HT	[6]
Charcosset	F2 × F252	F_5	129	SD	[7]
Chardon1	F7p × F2	$F_{2:3}$	150	DPS,SD	[8]
Chardon2	F7p × Gaspé	$F_{2:3}$	150	DPS,SD	[8]
Groh	CML131 × CML67	RIL	166	HT	[9]
Lubberstedt	KW1265 × D146	$F_{2:3}$	380	HT	[10]
Mechin	F2 × MBS847	F_5	100	SD,HT	[11]
Moreau	F2 × F252	F_3	300	SD	[12]
Blanc	DE,F283,F810,F9005	F_2	150 (per population)	SD,DPS	[2]
Pioneer	Unknown	$F_{4:5}$	976	HT	http://www.maizegdb.org
Poupard	F2 × MBS847	RIL	86	SD	[13]
Rebai	Unknown	$F_{2:3}$	1200	SD	[14]
Ribaut	Tropical	F_2	272	DPS,SD	[15]
Vladutu	E20 × N28	F_2	88	DPS,HT,LN	[16]

Table 1: 18 QTL mapping experiments related to flowering time in maize.

Bibliography

[1] Y. Barriere, G. Aurel, M. Briand, D. Denoue, and A. Gueu. QTL mapping for cell wall constituents and cell wall digestibility in maize recombinant inbred line progeny F838 X F286 harvested at an early forage stage of maturity. Technical report, INRA, 2005.

[2] G. Blanc, L. Moreau, B. Mangin, and A. Charcosset. QTL detection in connected populations of maize. In A. Caruna, editor, *12th Meeting of the EUCARPIA Section of Biometrics in Plant Breeding*, Spain, 2003.

[3] M. Bohn, M. M. Khairallah, D. Gonzalez-de Leon, D.A Hoisington, H. F. Utz, J. A. Deutsch, D. C. Jewel, J. A. Mihm, and A. E. Melchinger. QTL Mapping in Tropical Maize: I. Genomic Regions Affecting Leaf Feeding Resistance to Sugarcane Borer and Other Traits. *Crop. Sci.*, 36:1352–1361, 1996.

[4] M. Bohn, B. Schulz, R. Kreps, D. Klein, and A. E. Melchinger. QTL Mapping of resistance agaisnt the European corn borer (Ostrinia nubilalis H.) in early maturing European dent germplasm. *Theor. Appl. Genet.*, 1001:907–917, 2000.

[5] Agnes Bouchez, Frederic Hospital, Mathilde Causse, Andre Gallais, and Alain Charcosset. Marker-assisted introgression of favorable alleles at quantitative trait loci between maize elite lines. *Genetics*, 162(4):1945–1959, Dec 2002.

[6] A. J. Cardinal, M. Lee, N. Sharopova, W. L. Woodman-Clikeman, and M. J. Long. Genetics mapping and analysis of quantitative trait loci for resistance to stalk tunneling by the European corn borer in maize. *Crop. Sci.*, 41:835–845, 2001.

[7] A. Charcosset, M. Causse, L. Moreau, D. Vienne, and A. Gallais. Epistatic effect of genetic background on QTL expression in connected populations. Epistatis in Connected Population, 2000.

[8] Fabien Chardon, Delphine Hourcade, Valerie Combes, and Alain Charcosset. Mapping of a spontaneous mutation for early flowering time in maize highlights contrasting allelic series at two-linked QTL on chromosome 8. *Theor Appl Genet*, 112(1):1–11, Dec 2005.

[9] S. Groh, M. M. Khairallah, D. Gonzales-de Leon, M. Willcox, C. Jiang, D. A. Hoisington, and A. E. Melchinger. Comparison of QTLs mapped in RILs and their test-cross progenies of tropical maize for insect resistance and agronomic traits. *Plant. Breed.*, 117:193–202, 1998.

[10] T. Lubberstedt, A. Melchinger, C. Schon, H. F. Utz, and D. Klein. QTL mapping in testcrosses of European flint lines of maize: I. Comparison of different testers for forage yield traits. *Crop. Sci.*, 37:921–931, 1997.

[11] V. Mechin, O. Argillier, Y. Hebert, E. Guingo, L. Moreau, A. Charcosset, and Y. Barriere. Genetic analysis and QTL mapping of cell wall degistibility and lignification in silage maize. *Crop. Sci.*, 41:690–697, 2001.

[12] L. Moreau, A. Charcosset, and A. Gallais. Use of trail clustering to study QTL x environment effects for grain yield and related traits in maize. *Theor. Appl. Genet.*, 110:92–105, 2004.

[13] B. Poupard, L. Moreau, and A. Charcosset. Analyse de l'épistatsie entre QTL pour 3 caractères agronomiques chez le maïs. Technical report, INRA, 2001.

[14] A. Rebai, P. Blanchard, D. Perret, and P. Vincourt. Mapping quantitative trait loci controlling silking date in a diallel cross among four lines in maize. *Theor. Appl. Genet.*, 95:451–459, 1997.

[15] J.-M. Ribaut, D. Hoisington, J. A. Deutsch, C. Jiang, and D. Gonzalez-de Leon. Indentification of quantitative trait loci under drought conditions in tropical maize. I. Flowering parameters and the anthesis-silking interval. *Theor. Appl. Genet.*, 92:905–914, 1996.

[16] C. Vladutu, J. McLaughlin, and R. L. Phillips. Fine mapping and characterization of linked quantitative trait loci involved in the transition of the maize apical meristem from vegetative to generative structures. *Genetics*, 153(2):993–1007, Oct 1999.
