# Supplementary material for: MetaQTL: a package of new computational methods for the meta-analysis of QTL mapping experiments
Source: BMC Bioinformatics. 2007 Feb 8;8:49. doi: 10.1186/1471-2105-8-49 (PMC1808479; doi:10.1186/1471-2105-8-49)
Supplement: Additional File 7 — MetaQTL Package : jar file and tutorial. This Zip archive contains both the MetaQTL JAR file and the files of the tutorial. [file 1471-2105-8-49-S7.zip › org.inra.metaqtl/doc/org/thalia/bio/entity/AlleleContainer.html]

AlleleContainer


|  |  |  |  |  |  |  |  |  |  |  |
| --- | --- | --- | --- | --- | --- | --- | --- | --- | --- | --- |
| |  |  |  |  |  |  |  |  | | --- | --- | --- | --- | --- | --- | --- | --- | | **Overview** | **Package** | **Class** | **Use** | **Tree** | **Deprecated** | **Index** | **Help** | | |  |
| PREV CLASS   **NEXT CLASS** | **FRAMES**    **NO FRAMES**     **All Classes** |
| SUMMARY: NESTED | FIELD | CONSTR | METHOD | DETAIL: FIELD | CONSTR | METHOD |


---


## org.thalia.bio.entity Class AlleleContainer

```
java.lang.Object
  org.thalia.bio.entity.BioEntity
      org.thalia.bio.entity.BioEntityContainer
          org.thalia.bio.entity.AlleleContainer
```

**All Implemented Interfaces:**: IBioAdaptable, IBioEntity, IBioLocus

**Direct Known Subclasses:**: Locus

---

``` public abstract class AlleleContainer extends BioEntityContainer implements IBioLocus ```

Class Description Here

**Author:**
:   Jean-Baptiste Veyrieras

---

| **Field Summary** | |
| --- | --- |

| **Fields inherited from class org.thalia.bio.entity.BioEntityContainer** |
| --- |
| `entities` |

| **Fields inherited from class org.thalia.bio.entity.BioEntity** |
| --- |
| `name, parent, properties` |

| **Fields inherited from interface org.thalia.bio.IBioLocus** |
| --- |
| `MARKER, QTL, TypesString` |


| **Constructor Summary** | |
| --- | --- |
| `AlleleContainer()` |
| `AlleleContainer(java.lang.String name, IBioEntity parent)` |


| **Method Summary** | |
| --- | --- |
| `void` | `addAllele(IBioAllele allele)` |
| `IBioAllele[]` | `alleles()` |
| `IBioAllele` | `getAllele(java.lang.String name)` |
| `int` | `getAlleleNumber()` |
| `IBioLGroup` | `getGroup()` |
| `abstract  ILGroupPosition` | `getPosition()` |
| `abstract  int` | `getType()`             There are 2 main class of entities. |
| `void` | `removeAllele(java.lang.String name)` |
| `void` | `setGroup(IBioLGroup group)` |
| `abstract  void` | `setPosition(ILGroupPosition position)` |

| **Methods inherited from class org.thalia.bio.entity.BioEntityContainer** |
| --- |
| `addEntity, entities, entityNumber, getEntity, removeEntity` |

| **Methods inherited from class org.thalia.bio.entity.BioEntity** |
| --- |
| `getName, getParent, getProperties, newBioEntity, setName, setProperties` |

| **Methods inherited from class java.lang.Object** |
| --- |
| `clone, equals, finalize, getClass, hashCode, notify, notifyAll, toString, wait, wait, wait` |

| **Methods inherited from interface org.thalia.bio.IBioLocus** |
| --- |
| `getLocusType, setPosition, setPosition` |

| **Methods inherited from interface org.thalia.bio.IBioEntity** |
| --- |
| `getName, getParent, getProperties, setName, setProperties` |

| **Methods inherited from interface org.thalia.bio.IBioAdaptable** |
| --- |
| `getBioAdapter` |

| **Constructor Detail** |
| --- |

### AlleleContainer

```
public AlleleContainer()
```

---


### AlleleContainer

```
public AlleleContainer(java.lang.String name,
                       IBioEntity parent)
```

**Parameters:**: `name` -: `parent` -


| **Method Detail** |
| --- |

### getType

```
public abstract int getType()
```

:   **Description copied from interface: `IBioEntity`**
:   There are 2 main class of entities. The first one deals with population
    biological entity, i.e population itself and individuals. The second class
    is a representation of microscopic biological entity from genome container
    to alleles.

    :   **Specified by:**: `getType` in interface `IBioEntity` **Specified by:**: `getType` in interface `IBioLocus` **Specified by:**: `getType` in class `BioEntity`

---


### alleles

```
public IBioAllele[] alleles()
```

:   **Specified by:**: `alleles` in interface `IBioLocus`

---


### addAllele

```
public void addAllele(IBioAllele allele)
```

:   **Specified by:**: `addAllele` in interface `IBioLocus`

---


### removeAllele

```
public void removeAllele(java.lang.String name)
```

:   **Specified by:**: `removeAllele` in interface `IBioLocus`

---


### getAllele

```
public IBioAllele getAllele(java.lang.String name)
```

:   **Specified by:**: `getAllele` in interface `IBioLocus`

---


### getGroup

```
public IBioLGroup getGroup()
```

:   **Specified by:**: `getGroup` in interface `IBioLocus`

---


### setGroup

```
public void setGroup(IBioLGroup group)
```

:   **Specified by:**: `setGroup` in interface `IBioLocus`

---


### getAlleleNumber

```
public int getAlleleNumber()
```

:   **Specified by:**: `getAlleleNumber` in interface `IBioLocus`

---


### getPosition

```
public abstract ILGroupPosition getPosition()
```

:   **Specified by:**: `getPosition` in interface `IBioLocus`

---


### setPosition

```
public abstract void setPosition(ILGroupPosition position)
```

:   **Specified by:**: `setPosition` in interface `IBioLocus`


---


|  |  |  |  |  |  |  |  |  |  |  |
| --- | --- | --- | --- | --- | --- | --- | --- | --- | --- | --- |
| |  |  |  |  |  |  |  |  | | --- | --- | --- | --- | --- | --- | --- | --- | | **Overview** | **Package** | **Class** | **Use** | **Tree** | **Deprecated** | **Index** | **Help** | | |  |
| PREV CLASS   **NEXT CLASS** | **FRAMES**    **NO FRAMES**     **All Classes** |
| SUMMARY: NESTED | FIELD | CONSTR | METHOD | DETAIL: FIELD | CONSTR | METHOD |


---
