# Supplementary material for: MetaQTL: a package of new computational methods for the meta-analysis of QTL mapping experiments
Source: BMC Bioinformatics. 2007 Feb 8;8:49. doi: 10.1186/1471-2105-8-49 (PMC1808479; doi:10.1186/1471-2105-8-49)
Supplement: Additional File 7 — MetaQTL Package : jar file and tutorial. This Zip archive contains both the MetaQTL JAR file and the files of the tutorial. [file 1471-2105-8-49-S7.zip › org.inra.metaqtl/doc/org/thalia/bio/entity/BioEntityContainer.html]

BioEntityContainer


|  |  |  |  |  |  |  |  |  |  |  |
| --- | --- | --- | --- | --- | --- | --- | --- | --- | --- | --- |
| |  |  |  |  |  |  |  |  | | --- | --- | --- | --- | --- | --- | --- | --- | | **Overview** | **Package** | **Class** | **Use** | **Tree** | **Deprecated** | **Index** | **Help** | | |  |
| **PREV CLASS**   **NEXT CLASS** | **FRAMES**    **NO FRAMES**     **All Classes** |
| SUMMARY: NESTED | FIELD | CONSTR | METHOD | DETAIL: FIELD | CONSTR | METHOD |


---


## org.thalia.bio.entity Class BioEntityContainer

```
java.lang.Object
  org.thalia.bio.entity.BioEntity
      org.thalia.bio.entity.BioEntityContainer
```

**All Implemented Interfaces:**: IBioAdaptable, IBioEntity

**Direct Known Subclasses:**: AlleleContainer, GroupContainer, Individual, IndividualContainer, LocusContainer

---

``` public abstract class BioEntityContainer extends BioEntity implements IBioEntity ```

Class Description Here

**Author:**
:   Jean-Baptiste Veyrieras

---

| **Field Summary** | |
| --- | --- |
| `protected  java.util.HashMap` | `entities` |

| **Fields inherited from class org.thalia.bio.entity.BioEntity** |
| --- |
| `name, parent, properties` |


| **Constructor Summary** | |
| --- | --- |
| `BioEntityContainer()` |
| `BioEntityContainer(java.lang.String name, IBioEntity parent)` |


| **Method Summary** | |
| --- | --- |
| `protected  void` | `addEntity(IBioEntity entity)` |
| `protected  IBioEntity[]` | `entities()` |
| `protected  int` | `entityNumber()` |
| `protected  IBioEntity` | `getEntity(java.lang.String name)` |
| `protected  void` | `removeEntity(java.lang.String name)` |

| **Methods inherited from class org.thalia.bio.entity.BioEntity** |
| --- |
| `getName, getParent, getProperties, getType, newBioEntity, setName, setProperties` |

| **Methods inherited from class java.lang.Object** |
| --- |
| `clone, equals, finalize, getClass, hashCode, notify, notifyAll, toString, wait, wait, wait` |

| **Methods inherited from interface org.thalia.bio.IBioEntity** |
| --- |
| `getName, getParent, getProperties, getType, setName, setProperties` |

| **Methods inherited from interface org.thalia.bio.IBioAdaptable** |
| --- |
| `getBioAdapter` |

| **Field Detail** |
| --- |

### entities

```
protected java.util.HashMap entities
```


| **Constructor Detail** |
| --- |

### BioEntityContainer

```
public BioEntityContainer()
```

---


### BioEntityContainer

```
public BioEntityContainer(java.lang.String name,
                          IBioEntity parent)
```

**Parameters:**: `name` -: `parent` -


| **Method Detail** |
| --- |

### addEntity

```
protected void addEntity(IBioEntity entity)
```

---


### getEntity

```
protected IBioEntity getEntity(java.lang.String name)
```

---


### entityNumber

```
protected int entityNumber()
```

---


### entities

```
protected IBioEntity[] entities()
```

---


### removeEntity

```
protected void removeEntity(java.lang.String name)
```


---


|  |  |  |  |  |  |  |  |  |  |  |
| --- | --- | --- | --- | --- | --- | --- | --- | --- | --- | --- |
| |  |  |  |  |  |  |  |  | | --- | --- | --- | --- | --- | --- | --- | --- | | **Overview** | **Package** | **Class** | **Use** | **Tree** | **Deprecated** | **Index** | **Help** | | |  |
| **PREV CLASS**   **NEXT CLASS** | **FRAMES**    **NO FRAMES**     **All Classes** |
| SUMMARY: NESTED | FIELD | CONSTR | METHOD | DETAIL: FIELD | CONSTR | METHOD |


---
