# Supplementary material for: MetaQTL: a package of new computational methods for the meta-analysis of QTL mapping experiments
Source: BMC Bioinformatics. 2007 Feb 8;8:49. doi: 10.1186/1471-2105-8-49 (PMC1808479; doi:10.1186/1471-2105-8-49)
Supplement: Additional File 7 — MetaQTL Package : jar file and tutorial. This Zip archive contains both the MetaQTL JAR file and the files of the tutorial. [file 1471-2105-8-49-S7.zip › org.inra.metaqtl/doc/org/thalia/bio/entity/IGeneticMapProperties.html]

IGeneticMapProperties


|  |  |  |  |  |  |  |  |  |  |  |
| --- | --- | --- | --- | --- | --- | --- | --- | --- | --- | --- |
| |  |  |  |  |  |  |  |  | | --- | --- | --- | --- | --- | --- | --- | --- | | **Overview** | **Package** | **Class** | **Use** | **Tree** | **Deprecated** | **Index** | **Help** | | |  |
| **PREV CLASS**   **NEXT CLASS** | **FRAMES**    **NO FRAMES**     **All Classes** |
| SUMMARY: NESTED | FIELD | CONSTR | METHOD | DETAIL: FIELD | CONSTR | METHOD |


---


## org.thalia.bio.entity Interface IGeneticMapProperties

---

``` public interface IGeneticMapProperties ```

Class Description Here

**Author:**
:   Jean-Baptiste Veyrieras

---

| **Field Summary** | |
| --- | --- |
| `static java.lang.String[]` | `cartoFunction` |
| `static java.lang.String` | `cartoFunctionHaldane` |
| `static java.lang.String` | `cartoFunctionKey` |
| `static java.lang.String` | `cartoFunctionKosambi` |
| `static java.lang.String` | `distanceUnitCM` |
| `static java.lang.String` | `distanceUnitKey` |

| **Field Detail** |
| --- |

### cartoFunctionKey

```
static final java.lang.String cartoFunctionKey
```

**See Also:**: Constant Field Values

---


### cartoFunctionHaldane

```
static final java.lang.String cartoFunctionHaldane
```

**See Also:**: Constant Field Values

---


### cartoFunctionKosambi

```
static final java.lang.String cartoFunctionKosambi
```

**See Also:**: Constant Field Values

---


### cartoFunction

```
static final java.lang.String[] cartoFunction
```

---


### distanceUnitKey

```
static final java.lang.String distanceUnitKey
```

**See Also:**: Constant Field Values

---


### distanceUnitCM

```
static final java.lang.String distanceUnitCM
```

**See Also:**: Constant Field Values


---


|  |  |  |  |  |  |  |  |  |  |  |
| --- | --- | --- | --- | --- | --- | --- | --- | --- | --- | --- |
| |  |  |  |  |  |  |  |  | | --- | --- | --- | --- | --- | --- | --- | --- | | **Overview** | **Package** | **Class** | **Use** | **Tree** | **Deprecated** | **Index** | **Help** | | |  |
| **PREV CLASS**   **NEXT CLASS** | **FRAMES**    **NO FRAMES**     **All Classes** |
| SUMMARY: NESTED | FIELD | CONSTR | METHOD | DETAIL: FIELD | CONSTR | METHOD |


---
