# Supplementary material for: MetaQTL: a package of new computational methods for the meta-analysis of QTL mapping experiments
Source: BMC Bioinformatics. 2007 Feb 8;8:49. doi: 10.1186/1471-2105-8-49 (PMC1808479; doi:10.1186/1471-2105-8-49)
Supplement: Additional File 7 — MetaQTL Package : jar file and tutorial. This Zip archive contains both the MetaQTL JAR file and the files of the tutorial. [file 1471-2105-8-49-S7.zip › org.inra.metaqtl/doc/org/thalia/bio/entity/IndividualContainer.html]

IndividualContainer


|  |  |  |  |  |  |  |  |  |  |  |
| --- | --- | --- | --- | --- | --- | --- | --- | --- | --- | --- |
| |  |  |  |  |  |  |  |  | | --- | --- | --- | --- | --- | --- | --- | --- | | **Overview** | **Package** | **Class** | **Use** | **Tree** | **Deprecated** | **Index** | **Help** | | |  |
| **PREV CLASS**   **NEXT CLASS** | **FRAMES**    **NO FRAMES**     **All Classes** |
| SUMMARY: NESTED | FIELD | CONSTR | METHOD | DETAIL: FIELD | CONSTR | METHOD |


---


## org.thalia.bio.entity Class IndividualContainer

```
java.lang.Object
  org.thalia.bio.entity.BioEntity
      org.thalia.bio.entity.BioEntityContainer
          org.thalia.bio.entity.IndividualContainer
```

**All Implemented Interfaces:**: IBioAdaptable, IBioEntity, IBioPopulation

**Direct Known Subclasses:**: Population

---

``` public abstract class IndividualContainer extends BioEntityContainer implements IBioPopulation ```

Class Description Here

**Author:**
:   Jean-Baptiste Veyrieras

---

| **Field Summary** | |
| --- | --- |

| **Fields inherited from class org.thalia.bio.entity.BioEntityContainer** |
| --- |
| `entities` |

| **Fields inherited from class org.thalia.bio.entity.BioEntity** |
| --- |
| `name, parent, properties` |


| **Constructor Summary** | |
| --- | --- |
| `IndividualContainer()` |
| `IndividualContainer(java.lang.String name, IBioEntity parent)` |


| **Method Summary** | |
| --- | --- |
| `void` | `addIndividual(IBioIndividual individual)` |
| `IBioIndividual` | `getIndividual(java.lang.String name)` |
| `abstract  int` | `getIndividualNumber()` |
| `abstract  int` | `getType()`             There are 2 main class of entities. |
| `IBioIndividual[]` | `individuals()` |
| `void` | `removeIndividual(java.lang.String name)` |

| **Methods inherited from class org.thalia.bio.entity.BioEntityContainer** |
| --- |
| `addEntity, entities, entityNumber, getEntity, removeEntity` |

| **Methods inherited from class org.thalia.bio.entity.BioEntity** |
| --- |
| `getName, getParent, getProperties, newBioEntity, setName, setProperties` |

| **Methods inherited from class java.lang.Object** |
| --- |
| `clone, equals, finalize, getClass, hashCode, notify, notifyAll, toString, wait, wait, wait` |

| **Methods inherited from interface org.thalia.bio.IBioEntity** |
| --- |
| `getName, getParent, getProperties, setName, setProperties` |

| **Methods inherited from interface org.thalia.bio.IBioAdaptable** |
| --- |
| `getBioAdapter` |

| **Constructor Detail** |
| --- |

### IndividualContainer

```
public IndividualContainer()
```

---


### IndividualContainer

```
public IndividualContainer(java.lang.String name,
                           IBioEntity parent)
```

**Parameters:**: `name` -: `parent` -


| **Method Detail** |
| --- |

### getType

```
public abstract int getType()
```

:   **Description copied from interface: `IBioEntity`**
:   There are 2 main class of entities. The first one deals with population
    biological entity, i.e population itself and individuals. The second class
    is a representation of microscopic biological entity from genome container
    to alleles.

    :   **Specified by:**: `getType` in interface `IBioEntity` **Specified by:**: `getType` in class `BioEntity`

---


### getIndividual

```
public IBioIndividual getIndividual(java.lang.String name)
```

:   **Specified by:**: `getIndividual` in interface `IBioPopulation`

---


### addIndividual

```
public void addIndividual(IBioIndividual individual)
```

:   **Specified by:**: `addIndividual` in interface `IBioPopulation`

---


### removeIndividual

```
public void removeIndividual(java.lang.String name)
```

:   **Specified by:**: `removeIndividual` in interface `IBioPopulation`

---


### individuals

```
public IBioIndividual[] individuals()
```

:   **Specified by:**: `individuals` in interface `IBioPopulation`

---


### getIndividualNumber

```
public abstract int getIndividualNumber()
```

:   **Specified by:**: `getIndividualNumber` in interface `IBioPopulation`


---


|  |  |  |  |  |  |  |  |  |  |  |
| --- | --- | --- | --- | --- | --- | --- | --- | --- | --- | --- |
| |  |  |  |  |  |  |  |  | | --- | --- | --- | --- | --- | --- | --- | --- | | **Overview** | **Package** | **Class** | **Use** | **Tree** | **Deprecated** | **Index** | **Help** | | |  |
| **PREV CLASS**   **NEXT CLASS** | **FRAMES**    **NO FRAMES**     **All Classes** |
| SUMMARY: NESTED | FIELD | CONSTR | METHOD | DETAIL: FIELD | CONSTR | METHOD |


---
