# Supplementary material for: MetaQTL: a package of new computational methods for the meta-analysis of QTL mapping experiments
Source: BMC Bioinformatics. 2007 Feb 8;8:49. doi: 10.1186/1471-2105-8-49 (PMC1808479; doi:10.1186/1471-2105-8-49)
Supplement: Additional File 7 — MetaQTL Package : jar file and tutorial. This Zip archive contains both the MetaQTL JAR file and the files of the tutorial. [file 1471-2105-8-49-S7.zip › org.inra.metaqtl/doc/org/thalia/bio/entity/LGroup.html]

LGroup


|  |  |  |  |  |  |  |  |  |  |  |
| --- | --- | --- | --- | --- | --- | --- | --- | --- | --- | --- |
| |  |  |  |  |  |  |  |  | | --- | --- | --- | --- | --- | --- | --- | --- | | **Overview** | **Package** | **Class** | **Use** | **Tree** | **Deprecated** | **Index** | **Help** | | |  |
| **PREV CLASS**   **NEXT CLASS** | **FRAMES**    **NO FRAMES**     **All Classes** |
| SUMMARY: NESTED | FIELD | CONSTR | METHOD | DETAIL: FIELD | CONSTR | METHOD |


---


## org.thalia.bio.entity Class LGroup

```
java.lang.Object
  org.thalia.bio.entity.BioEntity
      org.thalia.bio.entity.BioEntityContainer
          org.thalia.bio.entity.LocusContainer
              org.thalia.bio.entity.LGroup
```

**All Implemented Interfaces:**: IBioAdaptable, IBioEntity, IBioLGroup

---

``` public class LGroup extends LocusContainer implements IBioLGroup ```

Class Description Here

**Author:**
:   Jean-Baptiste Veyrieras

---

| **Field Summary** | |
| --- | --- |

| **Fields inherited from class org.thalia.bio.entity.BioEntityContainer** |
| --- |
| `entities` |

| **Fields inherited from class org.thalia.bio.entity.BioEntity** |
| --- |
| `name, parent, properties` |


| **Constructor Summary** | |
| --- | --- |
| `LGroup()` |
| `LGroup(java.lang.String name, IBioEntity parent)` |


| **Method Summary** | |
| --- | --- |
| `IBioAdapter` | `getBioAdapter()` |
| `int` | `getType()`             There are 2 main class of entities. |
| `java.lang.String` | `toString()` |

| **Methods inherited from class org.thalia.bio.entity.LocusContainer** |
| --- |
| `addLocus, getGenome, getLocus, getLocusNumber, loci, removeLocus, setGenome, setLoci` |

| **Methods inherited from class org.thalia.bio.entity.BioEntityContainer** |
| --- |
| `addEntity, entities, entityNumber, getEntity, removeEntity` |

| **Methods inherited from class org.thalia.bio.entity.BioEntity** |
| --- |
| `getName, getParent, getProperties, newBioEntity, setName, setProperties` |

| **Methods inherited from class java.lang.Object** |
| --- |
| `clone, equals, finalize, getClass, hashCode, notify, notifyAll, wait, wait, wait` |

| **Methods inherited from interface org.thalia.bio.IBioLGroup** |
| --- |
| `addLocus, getGenome, getLocus, getLocusNumber, loci, removeLocus, setGenome, setLoci` |

| **Methods inherited from interface org.thalia.bio.IBioEntity** |
| --- |
| `getName, getParent, getProperties, setName, setProperties` |

| **Constructor Detail** |
| --- |

### LGroup

```
public LGroup()
```

---


### LGroup

```
public LGroup(java.lang.String name,
              IBioEntity parent)
```

**Parameters:**: `name` -: `parent` -


| **Method Detail** |
| --- |

### getType

```
public int getType()
```

:   **Description copied from interface: `IBioEntity`**
:   There are 2 main class of entities. The first one deals with population
    biological entity, i.e population itself and individuals. The second class
    is a representation of microscopic biological entity from genome container
    to alleles.

    :   **Specified by:**: `getType` in interface `IBioEntity` **Specified by:**: `getType` in class `LocusContainer`

---


### getBioAdapter

```
public IBioAdapter getBioAdapter()
```

:   **Specified by:**: `getBioAdapter` in interface `IBioAdaptable`

---


### toString

```
public java.lang.String toString()
```

:   **Overrides:**: `toString` in class `java.lang.Object`


---


|  |  |  |  |  |  |  |  |  |  |  |
| --- | --- | --- | --- | --- | --- | --- | --- | --- | --- | --- |
| |  |  |  |  |  |  |  |  | | --- | --- | --- | --- | --- | --- | --- | --- | | **Overview** | **Package** | **Class** | **Use** | **Tree** | **Deprecated** | **Index** | **Help** | | |  |
| **PREV CLASS**   **NEXT CLASS** | **FRAMES**    **NO FRAMES**     **All Classes** |
| SUMMARY: NESTED | FIELD | CONSTR | METHOD | DETAIL: FIELD | CONSTR | METHOD |


---
